# AURKA-mediated destabilization of SAPS3 drives ferroptosis evasion via 7-dehydrocholesterol biosynthesis in colorectal cancer

**DOI:** 10.1038/s41419-026-08549-9

**Published:** 2026-03-16

**Authors:** Jialing Gao, Weijing Zhang, Lulu Chen, Ruihan Pu, Shaoqing Huang, Xiaoxue Wu, Zhenshuang Du, Weiling He, Mei Song

**Affiliations:** 1 Department of Gastrointestinal Surgery, The First Affiliated Hospital, Sun Yat-Sen University, Guangzhou, Guangdong China; 2 Institute of Precision Medicine, The First Affiliated Hospital, Sun Yat-Sen University, Guangzhou, Guangdong China; 3 Department of Radiology, State Key Laboratory of Oncology in South China, Guangdong Provincial Clinical Research Center for Cancer, Sun Yat-sen University Cancer Center, Guangzhou, Guangdong China; 4 School of Public Health, Sun Yat-sen University, Guangzhou, Guangdong China; 5 Center of Hepato-Pancreatico-Biliary Surgery, The First Affiliated Hospital of Sun Yat-sen University, Guangzhou, Guangdong China; 6 Department of Gastrointestinal Surgery, Xiang’an Hospital of Xiamen University, School of Medicine, Xiamen University, Xiamen, Fujian China

**Keywords:** Cell death, Chemotherapy

## Abstract

While ferroptosis induction offers promising avenue for cancer therapeutics, its clinical utility in colorectal cancer (CRC) is limited by pervasive intrinsic resistance mechanisms. Here, we identify Aurora kinase A (AURKA) as a central suppressor of ferroptosis by rewiring cholesterol metabolism. Mechanistically, AURKA phosphorylates and destabilizes its negative regulator SAPS3 at Ser523/524, relieving AMPK suppression. Activated AMPK subsequently inhibits SREBP2 nuclear translocation and DHCR7 transcription, resulting in the accumulation of 7-dehydrocholesterol (7-DHC), a lipid antioxidant that confers ferroptosis resistance. Both genetic and pharmacologic inhibition of AURKA restore ferroptosis sensitivity and enhance chemotherapy efficacy in vitro and in patient-derived xenograft models. Clinically, elevated AURKA expression correlates with poor prognosis and reduced chemotherapy response in CRC patients. These findings delineate a novel AURKA-SAPS3-AMPK-SREBP2 axis that bridges cholesterol homeostasis and ferroptosis evasion, positioning AURKA as a promising therapeutic target for chemosensitization in CRC.

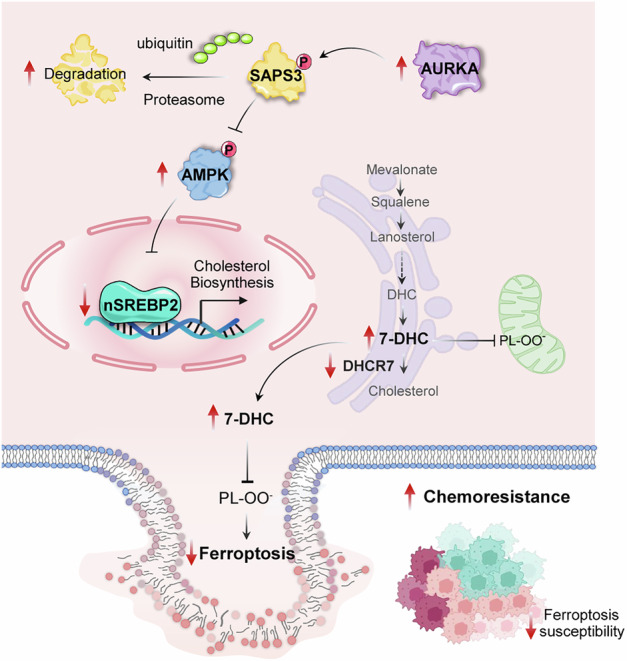

## Introduction

Ferroptosis is a nonapoptotic type of regulated cell death characterized by iron-dependent accumulation of lipid hydroperoxides [1, 2]. Emerging evidence suggests that inducing ferroptosis holds considerable promise for improving cancer treatment [3, 4], particularly to overcome chemoresistance [5–7]. This cellular process is orchestrated by intricate metabolic pathways, including lipid peroxidation, iron homeostasis, mitochondrial respiration, fatty acid metabolism, and glutathione (GSH) production [8, 9]. Recently, 7-dehydrocholesterol (7-DHC) has been identified as an anti-ferroptotic metabolite that reduces phospholipid peroxidation, pinpointing cholesterol biosynthesis in the modulation of ferroptotic sensitivity [10–12]. However, the upstream metabolic triggers of ferroptosis and the determinants of ferroptotic sensitivity remain elusive.

Colorectal cancer (CRC) is the third most prevalent malignancy globally, with high rates of recurrence and mortality [13]. Despite therapeutic advances, chemotherapy remains the cornerstone of treatment, particularly for unresectable cases. However, chemoresistance continues to be an intractable issue limiting its effectiveness [14]. Although growing evidence supports ferroptosis induction as a viable strategy to circumvent chemoresistance [14–17], the inherent insensitivity of CRC cells to ferroptosis constrains its chemosensitization potential [18, 19].

Aurora kinase A (AURKA) is a mitotic serine/threonine kinase essential for centrosome maturation during the G2 phase and mitotic spindle formation [20, 21]. Its crucial role in cell division is underscored by frequent overexpression or amplification in various cancers, a trait associated with poor clinical outcomes [22–26]. Moreover, AURKA possesses multiple non-mitotic oncogenic attributes, including inhibition of apoptosis [27, 28], promotion of metastasis [29, 30], and maintenance of stemness [31, 32], to foster tumor progression and drug resistance [33]. More recently, AURKA has been implicated in tumor metabolic regulation, involving amino acid synthesis, glucose metabolism, glycolysis, and fatty acid oxidation [34–37]. However, the mechanistic roles of AURKA in metabolic reprogramming-driven tumorigenesis remain poorly understood.

In this study, we demonstrate that AURKA functions as a ferroptosis suppressor in CRC by promoting 7-DHC biosynthesis. We show for the first time that AURKA directly phosphorylates SAPS3 at Ser523/524, destabilizing it through a kinase-dependent mechanism and subsequently modulating the AMPK-SREBP2-DHCR7 axis. Loss of AURKA enhances CRC chemosensitivity by transcriptionally upregulating DHCR7 via SREBP2, thereby converting the ferroptosis-protective metabolite 7-DHC to cholesterol. Our findings unveil a critical role for AURKA in orchestrating ferroptosis-cholesterol metabolic nexus and highlight AURKA inhibition as a promising therapeutic strategy for overcoming CRC chemoresistance.

## Materials and Methods

### Cell culture

The human colorectal cancer cell lines SW480 (RRID: CVCL_0546), RKO (RRID: CVCL_0504), HCT116 (RRID: CVCL_0291), DLD1 (RRID: CVCL_0248), SW1116 (RRID: CVCL_1724), SW620 (RRID: CVCL_0547) and human embryonic kidney (HEK) 293 T (RRID: CVCL_0063) were obtained from the American Type Culture Collection (ATCC). Cells were cultured at 37 °C with 5% CO_2_ in DMEM (Gibco) or RPMI 1640 (Gibco) medium supplemented with 10% fetal bovine serum (FBS), 1% penicillin/streptomycin (Gibco, 15140122) and 1% sodium pyruvate (Gibco, 11360070). All cell lines were authenticated using the Short Tandem Repeat (STR) method and tested negative for mycoplasma.

### Patient samples

The study was approved by the Ethics Committee of the First Affiliated Hospital, Sun Yat-sen University and conducted in accordance with recognized the ethical guidelines of the Declaration of Helsinki. All human tissue specimens, including TMAs containing paired CRC and adjacent normal tissues, RNA and protein samples from matched CRC tissues and adjacent normal tissues, CRC tissues for the PDX model and 52 CRC tissues with XELOX chemotherapy for IHC staining were obtained from the First Affiliated Hospital, Sun Yat-sen University with the consent of the patients. The clinicopathological characteristics of the CRC patients whose samples were included in this study are summarized in Supplementary Tables S1 and S2.

### Plasmid construction and transfection

pCMV-3×Flag-AURKA and pCMV-HA-SAPS3 were obtained from MiaolingBio (Wuhan, China). Mutant constructs of AURKA (D274N, T288D) and SAPS3 (S523A, S524A, S525A, S523A&S524A) were generated by site-directed mutagenesis and cloned into the pCMV-3×Flag and pcDNA4/myc-His A vectors, respectively. The primers used in the site-directed mutagenesis are listed in Supplementary Table S3. For plasmid transfection, experiments were conducted with Lipofectamine™ 3000 Transfection Reagent (Invitrogen, L3000008) according to the manufacturer’s instructions. To establish stable AURKA knockdown cell lines, shRNAs targeting the genomic sequences of AURKA were inserted into pLKO.1-puro (RRID: Addgene_8453) empty vector. Lentiviral particles were produced by transfecting HEK293T cells with pMD2.G (RRID: Addgene_12259), psPAX2 (RRID: Addgene_12260), and pLKO.1-shRNA plasmids at a 1:2:2 ratio using Lipo8000^TM^ reagent (Beyotime, C0533) for 48 h. Parental cells were then infected with shRNA-containing lentivirus for 24 h and selected with puromycin for 1–2 weeks. For siRNA-mediated AURKA, SCD, ETV4, DPEP1, DHCR7, SREBP2, SAPS3 silencing, cells were transfected with siRNA duplex and Lipofectamine RNAiMAX (Invitrogen, 13778075) according to the manufacturer’s instructions. A nonspecific siRNA oligo (Sigma, SIC002) was used as a negative control. The sequences of shRNA and siRNA are listed in Supplementary Table S4.

### Reagents

RSL3 (S8155), Erastin (S7242), FIN56 (S8254), 5-Fluorouracil (S1209), Oxaliplatin (S1224), Alisertib (S1133), AICAR (S1802), and Bafilomycin A1 (S1413) were obtained from Selleck (Houston, USA). AY9944 (HY-107420), 7-Dehydrocholesterol (HY-113279), and Cholesterol (HY-N0322) were obtained from MedChemExpress (Shanghai, China). Cycloheximide (01810), MG132 (M7449), and puromycin (P8833) were obtained from Sigma-Aldrich (Burlington, USA).

### Animal experiments

Female BALB/c nude mice (RRID: IMSR_CRL:194) and NOD/SCID mice (RRID: IMSR_JAX:001303) aged 4–6 weeks were purchased from the Guangdong GemPharmatech (Guangzhou, China). All animal experiments were conducted in accordance with the National Institute of Health Guide for the Care and Use of Laboratory Animals. The animals were maintained under specific pathogen-free conditions. The experimental procedures were approved by the Institutional Animal Care and Use Committee of the Sun Yat-sen University (approval number: SYSU-IACUC-2024001971).

For the subcutaneous xenograft model, 5 × 10^6^ SW480 cells were subcutaneously injected into the dorsal flank of randomized 4 to 6-week-old female nude mice.5-Fluorouracil monotherapy was started on day 7 post tumor inoculation, and intraperitoneal injections (25 mg/kg) were administered three times a week for 3 weeks. Tumor volume was measured every 3 days since day 7 of tumor implantation and calculated using the formula V = 0.5 × D × W^2^ (V, volume, D, diameter, and W, width). The mice were sacrificed on day 25, and the tumors were photographed and weighed.

For the PDX mouse model, fresh tumor tissues were subcutaneously implanted into the dorsal flank of mice as the first generation (F0). The patient’s profile is as follows: male, 55 years, Asian, with a diagnosis of moderately differentiated rectal adenocarcinoma, pathological stage pT3N1M0, clinical stage IIIB, microsatellite stable (MSS). Once reaching an appropriate volume, the tumors were excised, divided into equal pieces, and subcutaneously implanted into NOD/SCID mice as the second generation. 15 days post implantation, the tumor-bearing mice were randomly divided into four groups (vehicle, 5-Fluorouracil, alisertib, and combination). For single agent treatment 5-Fluorouracil was administered three times a week at 25 mg/kg by intraperitoneal injections. For single agent treatment alisertib was administered once daily at 30 mg/kg by oral gavage. For combination studies, 5-Fluorouracil was dosed at 25 mg/kg three times a week and alisertib was dosed at 30 mg/kg once daily.

### RNA extraction, reverse transcription and quantitative PCR

Total RNA was extracted using a Super FastPure Cell RNA Isolation Kit (Vazyme, RC102-01) according to the manufacturer’s instructions, and reversely transcripted into cDNA using the Evo M-MLV RT Kit (Accurate Biology, AG11706). Quantitative PCR was performed on a QuantStudio™ 5 F (Applied Biosystem™) using the SYBR Green Premix Pro Taq HS qPCR Kit (Accurate Biology, AG11701). The expression levels of the target genes were normalized with GAPDH abundance. The primers used for qRT-PCR are listed in Supplementary Table S5.

### RNA sequencing (RNA-seq) and analysis

Total RNA was extracted from SW480 cells (shNC vs sh*AURKA*) using TRIzol reagent (Invitrogen, 15596026). Subsequent RNA sequencing was performed on the MGISEQ2000 Platform (BGI, China). Differential gene analysis was performed using the DESeq2 R package under the conditions of Fold Change > 1 and adjusted *p*-value < 0.05. Using the pheatmap function on the differential gene set to draw a heatmap of differential gene clusters. The Kyoto Encyclopedia of Genes and Genomes (KEGG) pathway analysis was performed to annotate the biological functions of the DEGs using clusterProfiler R package.

### Cell proliferation, Cell viability and colony formation assay

For the cell proliferation assay, 2 × 10^4^ cells were seeded into a 96-well plate. Cell viability was measured at specific time points (1–4 days) using the Cell Counting Kit-8 (CCK-8) (MCE, HY-K0301) according to the manufacturer’s instructions. For the cell viability assay, 5 × 10^4^ cells were seeded into a 96-well plate and treated with the indicated chemicals for 48 h. Cell viability was measured using the CCK-8. For the colony forming assay, the indicated number of cells were seeded in a 6-well plate and treated continuously with chemo drugs. The plates were preincubated with 5% CO_2_ at 37 °C for 12–14 days. The colonies were fixed using 4% paraformaldehyde (PFA) (Biosharp, BL539A-1), stained with crystal violet staining solution (Beyotime, C0121), and counted with the Image J software (RRID: SCR_003070).

### Western blot

Cells were lysed using RIPA buffer (Beyotime, P0013B) supplemented with protease inhibitors and phosphatase inhibitors. Protein concentrations were measured with a BCA protein assay kit (Beyotime, P0011), and samples containing equal amounts of protein were used for immunoblotting analyses. Protein samples were separated on 10% SDS-PAGE and transferred to PVDF membrane (Merck Millipore, IPVH00010). Membranes were blocked in TBST containing 5% non-fat milk, incubated with primary antibodies according to the antibody manufacturer’s instructions, followed by incubation with horseradish peroxidase-conjugated goat anti-rabbit or anti-mouse IgG. The immunoblots were visualized to Immobilon Western Chemiluminescent HRP Substrate (Millipore, WBKLS0500) and scanned using an Amersham™ ImageQuant™ 800 system (Cytiva). To determine the half-life of protein, SW480 or RKO cells were exposed to CHX for varying durations (0, 2, 4, 8, 16, and 24 hours) and subsequently analyzed through Western blot. Quantification of immunoblotting signal intensities was performed using Image J software. The antibodies used for western blot analysis are listed in Supplementary Table S6.

### Immunoprecipitation (IP) assay

Cells were lysed using IP lysis buffer (Beyotime, P0013) supplemented with protease inhibitor and phosphatase inhibitors for 30 min on ice, centrifuged and quantified with BCA protein assay kit (Beyotime, P0011). Equal amounts of protein lysates for Co-IP were subjected to a preclearing step using 25 µl of Protein A/G PLUS-Agarose (Santa Cruz, sc-2002) for 1 h on ice, and then incubated with the primary antibodies and Protein A/G PLUS-Agarose on a rocker platform at 4 °C overnight. The beads were washed three times with IP lysis buffer and the precipitated proteins were eluted by boiling in 1 × SDS-PAGE loading buffer for 6 min and then subjected to western blot analysis. The antibodies used for IP assay are listed in Supplementary Table S6.

### Immunofluorescent staining

Cells were cultured on 15 mm Glass-bottomed cell culture dishes (Nest, 801002), fixed with 4% PFA for 10 min, permeabilized with 0.5% Triton X-100 (in PBS) for 10 min at room temperature, followed by blocking. Subsequently, the cells were incubated sequentially with primary antibodies, Alexa Fluor dye-conjugated secondary antibodies, and DAPI according to the manufacturer’s instructions. Confocal microscopy images were taken under Olympus FV3000 Laser Scanning Confocal Microscope.

### Hematoxylin and eosin (H&E) and immunohistochemistry (IHC) staining

IHC assays were conducted as previously described [14]. Paraffin-embedded tumor tissues were used to conduct H&E and IHC staining. Tissue sections were deparaffinized in xylene and rehydrated with graded ethanol (dilutions of 100%, 95%, 85% and 75%). Post rehydration, the sections were subjected to H&E staining. For IHC staining, the sections underwent rehydration, after which endogenous peroxidase activity was quenched with 3% H_2_O_2_ for 10 min, and heated in sodium citrate solution for antigen retrieval. After blocking with normal bovine serum albumin antigen for 1 h at room temperature, the tissue sections were incubated with the indicated primary antibody at 4 °C overnight, and then treated with secondary antibody and visualized with DAB Detection Kit (ZSGB-BIO, ZLI-9017). The tissue sections were counter stained with hematoxylin after DAB treatment, then dehydrated and stabilized with mounting medium and the images were acquired with a KF-PRO-020 scanner (Konfoong Tech). The antibodies used for IHC are listed in Supplementary Table S6. IHC analysis was performed by two independent observers who were blinded to the results of the other makers and clinical outcomes. Staining judging criteria: the gray density analysis method was used. Immunohistochemical images were identified and analyzed using Image J software to derive the integrated optical density (IOD) value. Average optical density (AOD) was calculated using the formula: AOD = IOD/Area. Subsequently, immunohistochemical images were statistically analyzed using GraphPad Prism 9.0 statistical software (RRID: SCR_002798).

### Ubiquitination assay

Cells were transfected with HA-ubiquitin constructs together with the indicated plasmids. The proteasome inhibitor MG132 (10 μM) was added 8 h before harvesting. At 36 h post-transfection, the cells were harvested and lysed in IP lysis buffer. Cell lysates were immunoprecipitated with anti-His as described in the IP assay. The bound proteins were eluted by boiling in 1 × SDS-PAGE loading buffer and subsequently analyzed by western blotting with the indicated antibodies.

### Purification of GST-tagged proteins from bacteria

Recombinant GST-tagged SAPS3 and its mutants were generated by transforming Escherichia coli BL21 with pGEX-4T-1-SAPS3-21aa-WT and pGEX-4T-1-SAPS3-21aa-DMut plasmids, respectively. Starter cultures grown overnight at 37 °C were inoculated at 1% into larger volumes of medium. Cultures were grown with vigorous shaking at 37°C to an OD600 of 0.8, followed by induction with 0.1 mM isopropyl-β-D-thiogalactopyranoside (IPTG) and incubation for 12–16 h at 16 °C. Pellets were re-suspended in EBC buffer and sonicated. The supernatant was incubated with glutathione sepharose beads for 3 h at 4 °C. The beads were washed three times with PBS and bound proteins were eluted using elution buffer. Proteins were analyzed by Coomassie blue staining and quantified using bovine serum albumin (BSA) as a standard.

### In vitro kinase assay

Bacterially purified GST-SAPS3-21AA-WT or GST-SAPS3-21AA-DMut protein (1 μg) was incubated with recombinant human AURKA (Cusabio, CSB-EP002454HU) in the presence of 200 μM adenosine triphosphate (ATP) and 100 ng adenosine 5′-thiotriphosphate in kinase reaction buffer (50 mM Tris-HCl, pH 7.5; 1 mM MgCl₂; 2 mM DTT; 1 mM EGTA) at 30 °C for 30 min. The phosphorylation of SAPS3 was subsequently analyzed by western blotting.

### LC-MS/MS analysis for 7-DHC and cholesterol

Culture cells were trypsinized and washed twice with pre-cooled PBS. Subsequently, 600 μl of hexane/Isopropyl alcohol (v/v, 3:2) was added and the sample was vortexed for 2 min to ensure homogeneity, followed by sonication for 60 s in an ice-water bath. The sample was centrifuged at 12,000 rpm for 10 min at 4 °C to pellet protein and other insoluble material. The supernatant was removed to a new tube, and another 400 μl of hexane/Isopropyl alcohol (v/v, 3:2) was added for the second extraction, and the supernatant was collected to the tube containing the same sample. Samples were centrifuged at 12,000 rpm for 10 min at 4 °C. The organic layer was collected to a new tube and was dried under N2 using a 12-port drying manifold.

Authentic standards of 7-DHC (Sigma, 30800) and cholesterol (Sigma, C8667) were used for calibration. LC–MS analysis was performed using an Agilent 1290 Infinity II Preparative LC System coupled to an Agilent 6495 A Triple Quadrupole LC/MS. Chromatography separation was performed under gradient conditions using the Agilent C18 column (2.1 × 100 mm, 2.7 μm) for separation. The mobile phase was (A) 10% methanol containing 0.05% formic acid and (B) methanol containing 0.05% formic acid. The flow rate was 0.60 ml min^−1^, and the column was maintained at 15 °C.

For 7-DHC and cholesterol, elution from the column was performed over 13.5 min with the following gradient: 92% B increased to 96% B in 10 min, held at 96% B for 1 min, then returned to 92% B in 0.5 min and equilibrated for 2 min.

### Flow cytometry analysis

For cellular lipid ROS detection, cells were seeded into a 6-well plates. After treatment, cells were washed and stained with 10 μM BODIPY™ 581/591C11 probe (Thermo Fisher, D3861) in the dark at 37 °C for 1 h. The cells were then disassociated and washed with PBS for three times. For apoptosis analysis, culture cells were washed, dissociated, and suspended in 1 × binding buffer. The cells were then stained with Annexin V and PI (4 A Biotech, FXP023) at room temperature for 15 min in the dark. Data acquisition was performed on the flow cytometer CytoFLEX (Beckman) and analyzed via FlowJo 10.8.1 (RRID: SCR_008520).

### MDA and GSH assay

The levels and activities of MDA and GSH were detected using the MDA assay kit (Nanjing, A003-1) and GSH assay kits (Beyotime, S0053) according to the manufacturer’s instructions. All measurements were subsequently normalized to protein levels, which were determined using the BCA Protein Assay Kit.

### Mass spectrometry analysis

SW480 and RKO cells stably expressing Flag-vector or Flag-AURKA were lysed in IP lysis buffer and immune-precipitated with anti-FLAG antibody (Sigma, F1804) and Protein A/G PLUS-Agarose (Santa Cruz, sc-2003). The precipitated complexes were then boiled at 95 °C for 6 min and separated via SDS–PAGE. After staining with Coomassie Brilliant Blue (Beyotime, P0017), we excised bands with significant differences after precipitation for LC–MS/MS identification, which was performed to analyze on the Orbitrap FUSION LUMOS (Thermo Fisher Scientific) by BGI Genomics. For the identification of SAPS3 phosphorylation sites, HA-SAPS3 proteins were immunoprecipitated from SW480 cells stably expressing HA-SAPS3 and Flag-AURKA, and the precipitated complexes were boiled at 95 °C for 6 min and separated via SDS-PAGE. After staining with Coomassie Brilliant Blue, we excised the SAPS3 bands for LC–MS/MS identification, which was performed to analyze the phosphorylation modification sites on timsTOF Pro (Bruker) and nanoElute (Bruker) by APTBIO.

### Bioinformatic analysis

The data of differentially expressed genes between colorectal cancer and normal tissues were extracted from GEO datasets GSE44076, GSE9348, and GSE23878, and ferroptosis-related genes were obtained from the FerrDb V2 database. TCGA database was used to compare the expression of AURKA between normal tissues and colorectal cancer tissues. Kaplan-Meier plotter was used to analyze the survival rate of patients according to AURKA expression. We utilized tissue chips from SYSU-FAH to analyze the correlation between AURKA expression and the survival of CRC patients.

### Statistical analysis

Experiments were independently repeated at least three times. For the in vivo experiments, mice were randomly assigned to different groups. Normality was tested using the Shapiro-Wilk test. All values shown in graphs are presented as individual data points and as the mean ± SD, unless stated otherwise. Comparisons between the two groups were performed using an unpaired two-sided Student’s t-test. The survival rates were compared by the log-rank test (*p* < 0.05 was considered statistically significant). Comparison of multiple conditions was done with One-way or two-way ANOVA test.

## Results

### Identification of AURKA as a ferroptosis suppressor in CRC

**Fig. 1:**
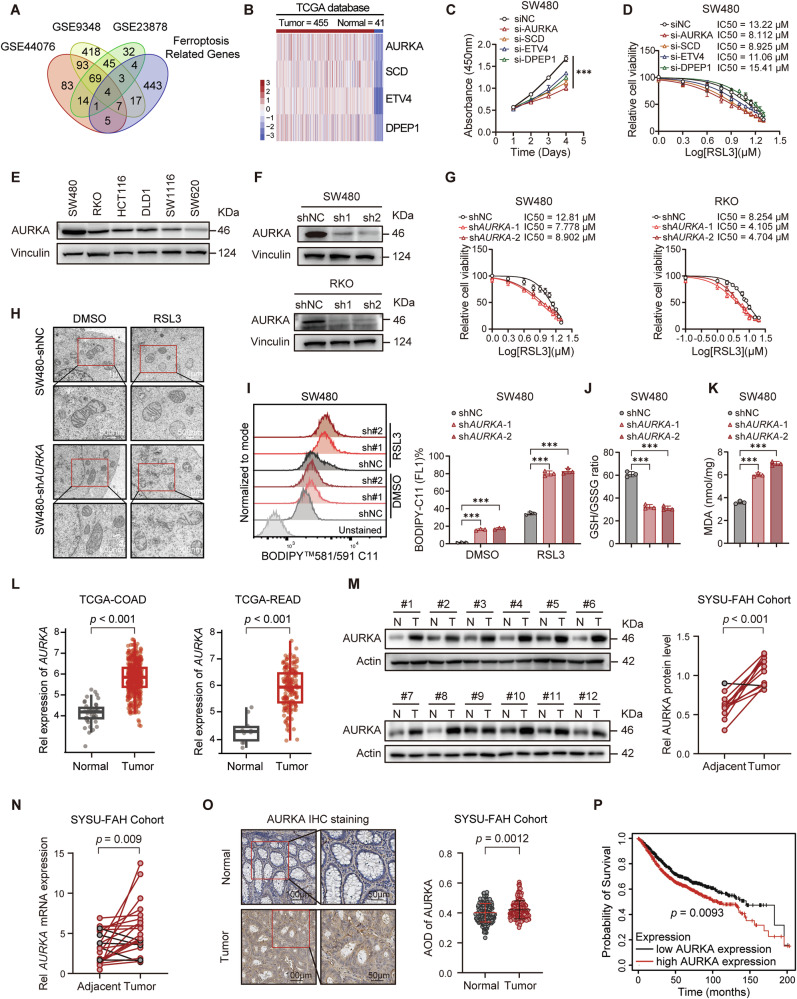
AURKA is overexpressed in CRC and suppresses ferroptosis. **A** Venn diagram showing overlapping differentially expressed genes (DEGs) between GSE44076, GSE9348, GSE23878 datasets and a curated ferroptosis-related gene set. **B** Heatmap demonstrating expression patterns of four overlapping genes in CRC versus normal tissues from the TCGA database. **C**, **D** CCK-8 assays measuring viability of SW480 cells after siRNA-mediated knockdown of AURKA, SCD, ETV4, and DPEP1 (**C**) and following RSL3 treatment for 48 h (**D**). **E**, **F** Western blot analysis of AURKA protein levels across various human CRC cell lines (**E**) or validation of shRNA-mediated stable AURKA knockdown efficiency in SW480 and RKO cells (**F**). **G** IC_50_ analysis of RSL3 in CRC (shNC and sh*AURKA*) cells treated with different concentrations of RSL3 for 48 h. **H** Representative TEM images of SW480 (shNC and sh*AURKA*) cells after the indicated treatment with RSL3 (5 μM) for 24 h. **I** Representative FACS images and quantified values of lipid ROS levels in SW480 (shNC and sh*AURKA*) cells treated with RSL3 (5 μM) for 24 h. **J**, **K** Intracellular GSH/GSSG ratios (**J**) and cellular concentrations of MDA (**K**) were evaluated in SW480 (shNC and sh*AURKA*) cells. **L** *AURKA* mRNA levels in normal versus tumor specimens from TCGA colon adenocarcinoma (COAD) and rectum adenocarcinoma (READ) database. **M** Western blot analysis of AURKA protein expression in 12 paired adjacent normal (N) and CRC (T) tissues from SYSU-FAH cohort, with quantitative normalization to β-actin. **N** qPCR analysis of *AURKA* mRNA expression in 22 paired CRC tissues from SYSU-FAH cohort. **O** IHC staining and quantitative analysis of AURKA expression in CRC tumor microarrays (TMAs). Scale bars: 50 µm. **P** Overall survival analysis based on AURKA expression in Kaplan-Meier Plotter CRC cohort (*n* = 1061). Data are presented as mean ± SD from at least three independent experiments. Statistical analyses were performed using unpaired Student’s *t* test [(**I**), (**J**), (**K**), (**L**), and (**O**)], paired Student’s *t* test [(**M**) and (**N**)], two-way ANOVA test (**C**), or log-rank test (**P**). ***p < 0.001.

To unravel the molecular determinants of ferroptosis sensitivity in CRC, we interrogated the FerrDB and COAD-GEO databases, which led to the identification of four candidate ferroptosis modulators: AURKA, SCD, ETV4, and DPEP1 (Fig. 1A, B). Among these, AURKA knockdown exerted the most profound inhibitory effect on CRC cell proliferation and markedly augmented the susceptibility of CRC cells to the ferroptosis inducer RSL3 (Fig. 1C, D; Supplementary Fig. S1A, B). Although AURKA is known to inhibit apoptosis, necroptosis and autophagy [38], its role as an anti-ferroptotic factor, particularly in CRC, remains poorly characterized. We further investigated its implications in ferroptosis resistance by silencing AURKA in the CRC cell lines SW480 and RKO, both of which exhibit high AURKA protein expression (Fig. 1E, F). This depletion induced a series of ferroptotic responses, including reduced IC_50_ values for RSL3, mitochondrial damage and shrinkage, heightened levels of lipid reactive oxygen species (ROS) and malondialdehyde (MDA), and reduced GSH/GSSG redox ratios (Fig. 1G–K; Supplementary Fig. S1C–F). Crucially, these ferroptotic features manifested even under basal conditions, and AURKA depletion sensitized cells to additional ferroptosis inducers, including erastin and FIN56, suggesting that AURKA sustains constitutive ferroptosis resistance in CRC cells (Supplementary Fig. S1G, H). Analysis of The Cancer Genome Atlas (TCGA) revealed that AURKA expression was upregulated in both colon adenocarcinoma (COAD) and rectal adenocarcinoma (READ) tissues (Fig. 1L). This upregulation was validated in paired CRC tissue samples collected from the First Affiliated Hospital of Sun Yat-Sen University (SYSU-FAH) and further confirmed by immunohistochemical (IHC) analysis of tissue microarrays (TMAs) consisting of 224 paired CRC specimens (Fig. 1M–O). High AURKA expression was associated with unfavorable outcomes in CRC patients (Fig. 1P). These findings suggest that AURKA may serve as a crucial ferroptotic suppressor in CRC.

### AURKA inhibits the transcription of the pro-ferroptotic gene DHCR7

**Fig. 2:**
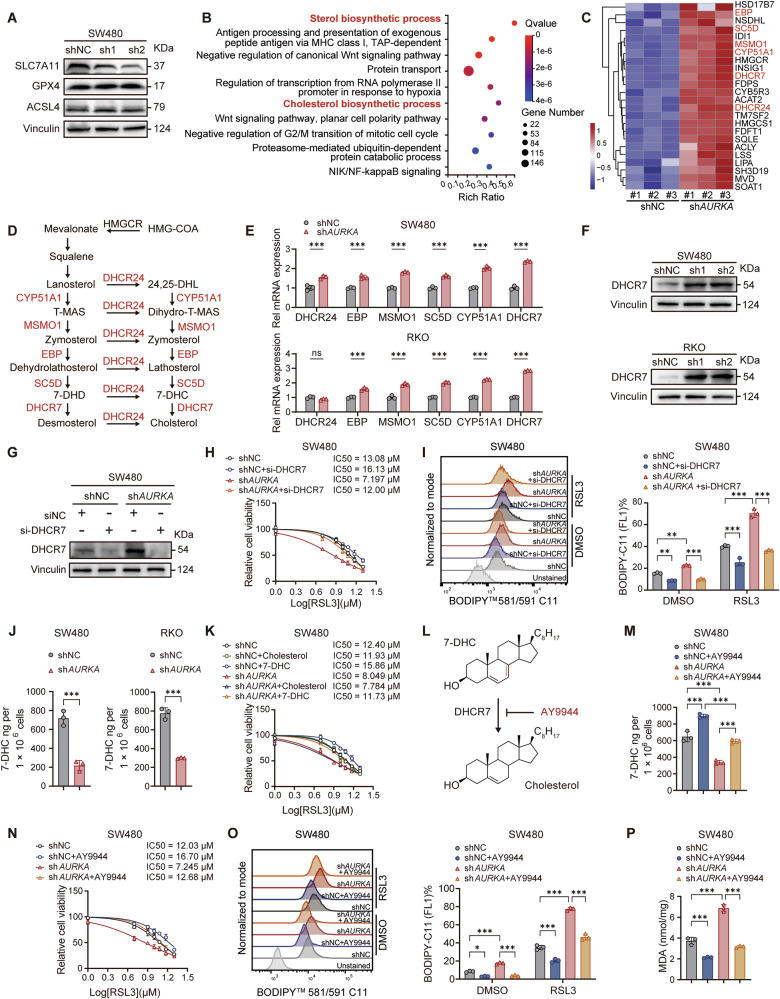
AURKA suppresses ferroptosis by blocking DHCR7-mediated 7-DHC catabolism. **A** Western blot analysis of SLC7A11, GPX4, and ACSL4 in SW480 (shNC and sh*AURKA*) cells. **B**, **C** RNA-seq with KEGG enrichment analysis in SW480 (shNC and sh*AURKA*) cells showing upregulated pathways in sh*AURKA* group (**B**), and a heatmap depicting DEGs in sterol/cholesterol biosynthesis pathways (**C**). **D** Schematic of the cholesterol biosynthesis pathway and its regulatory enzymes. **E** qPCR quantification of cholesterol biosynthesis enzyme mRNA levels in CRC (shNC and sh*AURKA*) cells. **F**, **G** Western blot analysis of DHCR7 protein expression in SW480 and RKO cells (**F**), and SW480 cells following siRNA knockdown (**G**). **H** IC_50_ analysis of RSL3 in SW480 (shNC and sh*AURKA*) cells treated with RSL3 (48 h) following DHCR7 silencing. **I** Representative FACS images and quantified values of lipid ROS levels in SW480 (shNC and sh*AURKA*) cells treated with RSL3 (5 μM, 24 h) following DHCR7 silencing. **J** LC-MS/MS quantification of 7-DHC levels in CRC (shNC and sh*AURKA*) cells. **K** Quantification of RSL3 IC_50_ values in SW480 (shNC and sh*AURKA*) cells after pretreatment with 7-DHC (25 μM), cholesterol (25 μM) for 24 h. **L** Diagram of cholesterol synthesis showing DHCR7 converting 7-DHC to cholesterol and its inhibition by AY9944. **M** Intracellular levels of 7-DHC in SW480 (shNC and sh*AURKA*) cells treated with AY9944 (100 nM) for 24 h. **N** Quantification of RSL3 IC_50_ values in SW480 (shNC and sh*AURKA*) cells after pretreatment with AY9944 (100 nM) for 24 h. **O,** **P** Quantification of intracellular lipid ROS (**O**) and MDA (**P**) levels in SW480 (shNC and sh*AURKA*) cells treated with AY9944 (100 nM) for 24 h. Data are presented as mean ± SD from at least three independent experiments. Statistical analyses were performed using unpaired Student’s *t* test [(**E**), (**I**), (**J**), (**M**, **O**, **P**)]. *p < 0.05, **p < 0.01, ***p < 0.001.

In CRC cells, AURKA depletion had minimal effects on canonical ferroptosis regulators such as GPX4, ACSL4, and SLC7A11, suggesting that its ferroptosis-modulating function operates independently of classical pathways (Fig. 2A; Supplementary Fig. S2A). To systematically elucidate the mechanisms underlying AURKA-conferred ferroptosis resistance, we performed RNA-seq transcriptome analysis, which revealed prominent upregulation of the sterol and cholesterol biosynthesis pathways in AURKA-depleted cells (Fig. 2B; Supplementary Fig. S2B). Key SREBP2-regulated enzymes involved in cholesterol biosynthesis, including EBP, SCD5, MSMO1, CYP51A1, and DHCR7, were transcriptionally upregulated (Fig. 2C, D). Strikingly, DHCR7, which encodes the terminal enzyme converting 7-dehydrocholesterol (7-DHC) to cholesterol, emerged as the most dynamically responsive gene (Fig. 2E, F), consistent with its reported pro-ferroptotic role via 7-DHC depletion [10, 11]. Genetic ablation of DHCR7 in sh*AURKA* CRC cells fully abolished their ferroptotic hypersensitivity and restored resistance to RSL3 (Fig. 2G–I; Supplementary Fig. S2C–E). Notably, quantitative LC-MS/MS analysis confirmed that AURKA silencing selectively reduced intracellular 7-DHC levels (Fig. 2J), with only a marginal decrease in cholesterol (Supplementary Fig. S2F). This selective 7-DHC depletion aligned with its established role in quenching phospholipid autoxidation, a critical driver of lipid peroxidation [10, 11]. In our system, exogenous 7-DHC supplementation, but not equimolar cholesterol, reversed AURKA deficiency-induced ferroptosis vulnerability, highlighting the specificity of 7-DHC in redox protection (Fig. 2K; Supplementary Fig. S2G). Furthermore, pharmacological inhibition of DHCR7 with the selective inhibitor AY9944 restored intracellular 7-DHC levels in sh*AURKA* cells to baseline levels comparable to those in controls, without affecting intracellular cholesterol concentrations (Fig. 2L, M; Supplementary Fig. S2H). Critically, AY9944 treatment effectively countered the AURKA knockdown-augmented ferroptosis (Fig. 2N–P; Supplementary Fig. S2I–L). Collectively, these data suggest that AURKA dictates ferroptosis sensitivity by modulating 7-DHC levels through DHCR7 transcriptional control.

### AURKA suppresses DHCR7 transcription through AMPK-SREBP2 signaling

**Fig. 3:**
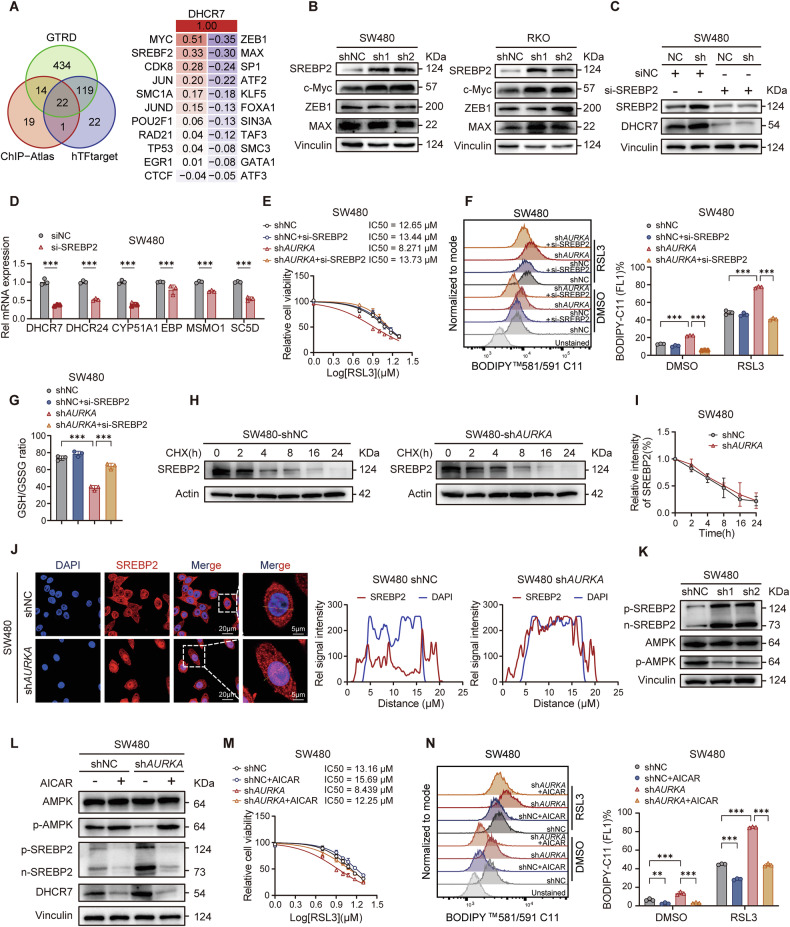
AURKA inhibits DHCR7 transcription via the AMPK-SREBP2 signaling axis. **A** Venn diagram analysis of transcription factors predicated to regulate *DHCR7* gene transcription based on ChIP-Atlas, GTRD, and hTFtarget databases. **B** Western blot analysis of SREBP2, c-Myc, ZEB1, and MAX in CRC (shNC and sh*AURKA*) cells. **C** Western blot analysis of SREBP2 and DHCR7 protein levels in SW480 cells after siRNA-mediated SREBP2 knockdown. **D** qPCR quantification of cholesterol biosynthesis enzyme mRNA levels in SREBP2-silenced SW480 cells. **E** IC_50_ analysis of RSL3 in SREBP2-silenced SW480 (shNC and sh*AURKA*) cells treated with RSL3 (48 h). **F**, **G** Representative FACS images and quantified values of lipid ROS levels (**F**) and GSH/GSSG ratios (**G**) in SREBP2-silenced SW480 (shNC and sh*AURKA*) cells treated with RSL3 (5 μM, 24 h). **H**, **I** Western blot analysis of SREBP2 at indicated timepoints after CHX (100 µg/ml) treatment (**H**), and quantified degradation kinetics (**I**) in SW480 cells. **J** Immunofluorescence (IF) analyses of subcellular distribution of SREBP2 in SW480 (shNC and sh*AURKA*) cells. **K**, **L** Western blot analysis of pSREBP2, nSREBP2, total AMPK, and pAMPK levels in SW480 (shNC and sh*AURKA*) cells (**K**) and post AICAR treatment (1 mM, 24 h, **L**). **M** IC_50_ analysis of RSL3 in SW480 (shNC and sh*AURKA*) cells after pretreatment with AICAR (1 mM, 24 h). **N** Representative FACS images and quantified values of lipid ROS levels in SW480 (shNC and sh*AURKA*) cells treated with AICAR (1 mM, 24 h). Data are presented as mean ± SD from at least three independent experiments. Statistical analyses were performed using unpaired Student’s *t* test [(**D**), (**F**), (**G**), and (**N**)]. **p < 0.01, ***p < 0.001.

To unbiasedly identify transcription factor(s) (TFs) mediating AURKA-dependent regulation of *DHCR7*, we integrated multi-omics TF prediction data (ChIP-Atlas, GTRD, hTF target databases) with TCGA co-expression analysis. Of the candidate TFs, c-Myc and SREBP2 (sterol regulatory element-binding protein 2) demonstrated a positive correlation with DHCR7, whereas ZEB1 and MAX exhibited an inverse correlation (Fig. 3A). Of note, AURKA depletion selectively upregulated SREBP2 expression in CRC cells, with minimal impact on c-Myc, ZEB1 and MAX (Fig. 3B). Knocking down SREBP2 significantly downregulated cholesterol biosynthesis genes, with the most pronounced effect on DHCR7 transcription (Fig. 3C, D; Supplementary Fig. S3A, B), confirming SREBP2 as the dominant transcriptional regulator of sterol metabolism. SREBP2 depletion in sh*AURKA* CRC cells robustly attenuated ferroptosis sensitivity, with reduced lipid ROS while heightened GSH/GSSG ratios (Fig. 3E–G; Supplementary Fig. S3C–E). Although AURKA did not alter the total SREBP2 protein stability (Fig. 3H, I; Supplementary Fig. S3F, G), its depletion enhanced nuclear translocation of the mature SREBP2 form (Fig. 3J, K; Supplementary Fig. S3H), indicative of augmented transcriptional activity. It is known that adenosine monophosphate (AMP)-activated protein kinase (AMPK) phosphorylates SREBP2 precursor to block its nuclear translocation in primary hepatocytes [39–41]. We consistently detected reduced AMPK phosphorylation in AURKA-depleted CRC cells (Fig. 3K; Supplementary Fig. S3H). Pharmacological activation of AMPK with AICAR reduced nuclear SREBP2 levels and suppressed *DHCR7* expression (Fig. 3L; Supplementary Fig. S3I), mirroring the ferroptosis-protective effects observed with SREBP2 knockdown, including increased resistance to RSL3 and reduced lipid peroxidation (Fig. 3M, N; Supplementary Fig. S3J, K). Of note, neither SREBP2 silencing nor AMPK activation significantly altered ferroptosis vulnerability in control CRC cells. These findings collectively demonstrate that AURKA suppresses ferroptosis by modulating the AMPK-SREBP2-DHCR7 signaling axis.

### AURKA prevents ferroptosis by interacting with SAPS3 in CRC

**Fig. 4:**
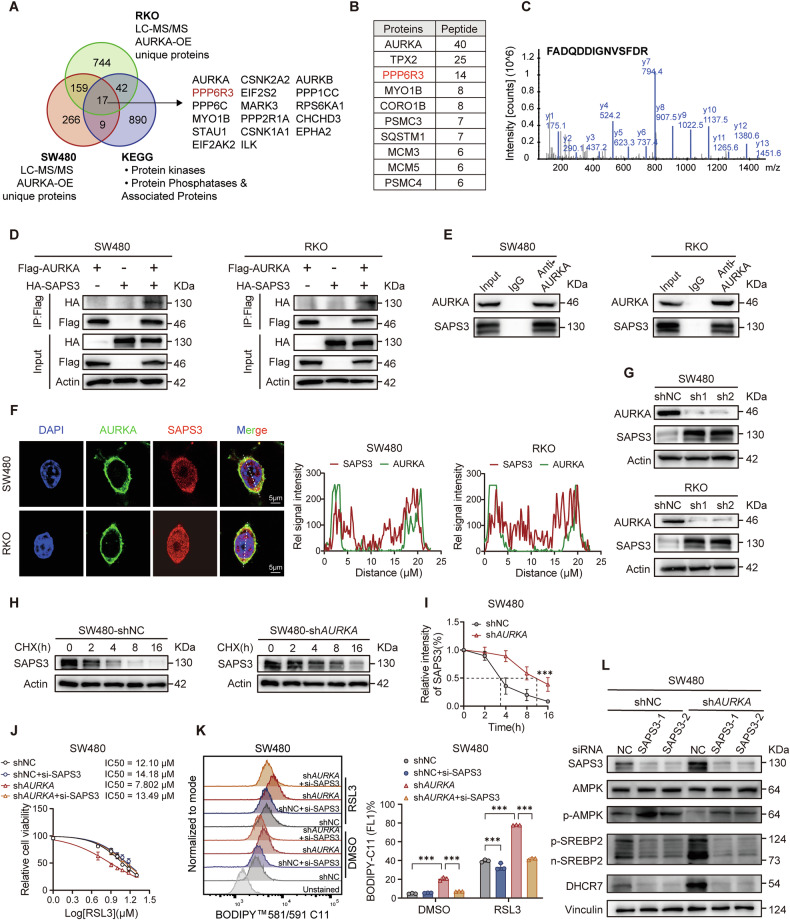
AURKA interacts with SAPS3 to prevent ferroptosis in CRC. **A** Immunoprecipitation-mass spectrometry (IP-MS) analysis of AURKA-interacting kinases/phosphatases. **B** Top 10 AURKA-binding proteins identified by mass spectrometry. **C** Representative SAPS3 peptide spectrum from IP-MS. **D** Co-IP assay showing the interaction between exogenous Flag-AURKA with HA-SAPS3 in SW480 and RKO cells. **E** Endogenous AURKA-SAPS3 interaction confirmed by Co-IP using anti-AURKA antibody in SW480 and RKO cells. **F** Immunofluorescent staining showing co-localization of endogenous AURKA and SAPS3 in SW480 and RKO cells. Scale bars: 5 µm. **G** Western blot analysis of SAPS3 in SW480 and RKO cells. **H**, **I** Western blot analysis of SAPS3 at indicated timepoints after CHX (100 µg/ml) treatment (**H**), and quantified degradation kinetics (**I**) in SW480 cells. **J** IC_50_ analysis of RSL3 in SW480 (shNC and sh*AURKA*) cells treated with RSL3 (48 h) following SAPS3 knockdown. **K** Representative FACS images and quantified values of lipid ROS levels in SW480 (shNC and sh*AURKA*) cells after SAPS3 silencing. **L** Western blot analysis of total AMPK, pAMPK, pSREBP2, nSREBP2, and DHCR7 levels in SW480 (shNC and sh*AURKA*) cells post SAPS3 knockdown. Data are presented as mean ± SD from at least three independent experiments. Statistical analyses were performed using unpaired Student’s *t* test (**K**) and two-way ANOVA test (**I**). ***p < 0.001.

To pinpoint the specific protein kinases/phosphatases that interact with AURKA and regulate AMPK phosphorylation, we performed co-immunoprecipitation followed by mass spectrometry (IP-MS) using Flag-tagged AURKA. AURKA itself, along with its well-established cofactor TPX2, were among the most abundant peptides detected, validating the reliability of the methodology (Fig. 4A, B). Notably, PPP6R3 (protein phosphatase 6 regulatory subunit 3), also known as SAPS3, was identified as the third most enriched peptide in the interactome (Fig. 4C). This observation aligns with prior studies demonstrating that PPP6R3 recruits the catalytic subunit of PP6 (PPP6C), which was also detected in our analysis, to dephosphorylate AMPK [42]. Subsequent co-immunoprecipitation (Co-IP) confirmed a direct interaction between Flag-tagged AURKA and HA-tagged SAPS3, as well as endogenous AURKA and SAPS3 in CRC cells, with co-localization observed primarily in the cytosol (Fig. 4D–F). In addition, loss of AURKA in CRC cells led to elevated SAPS3 protein expression (Fig. 4G), suggesting that AURKA negatively regulates SAPS3 levels. Consistent with this, cycloheximide (CHX) chase assays revealed that AURKA promoted SAPS3 degradation (Fig. 4H, I; Supplementary Fig. S4A, B). Functionally, SAPS3 knockdown abolished the ferroptosis hypersensitivity observed in sh*AURKA* cells, restoring ferroptosis resistance to baseline levels, while having negligible impact in control cells (Fig. 4J, K; Supplementary Fig. S4C, D). Since SAPS3 is a known negative regulator of AMPK, its depletion resulted in increased AMPK phosphorylation, impaired nuclear translocation of SREBP2 and reduced DHCR7 expression (Fig. 4L; Supplementary Fig. S4E). These findings imply that AURKA dictates the ferroptosis sensitivity of CRC through its direct interaction with SAPS3.

### AURKA modulates the AMPK-SREBP2-DHCR7 axis via kinase activity-dependent phosphorylation of SAPS3

**Fig. 5:**
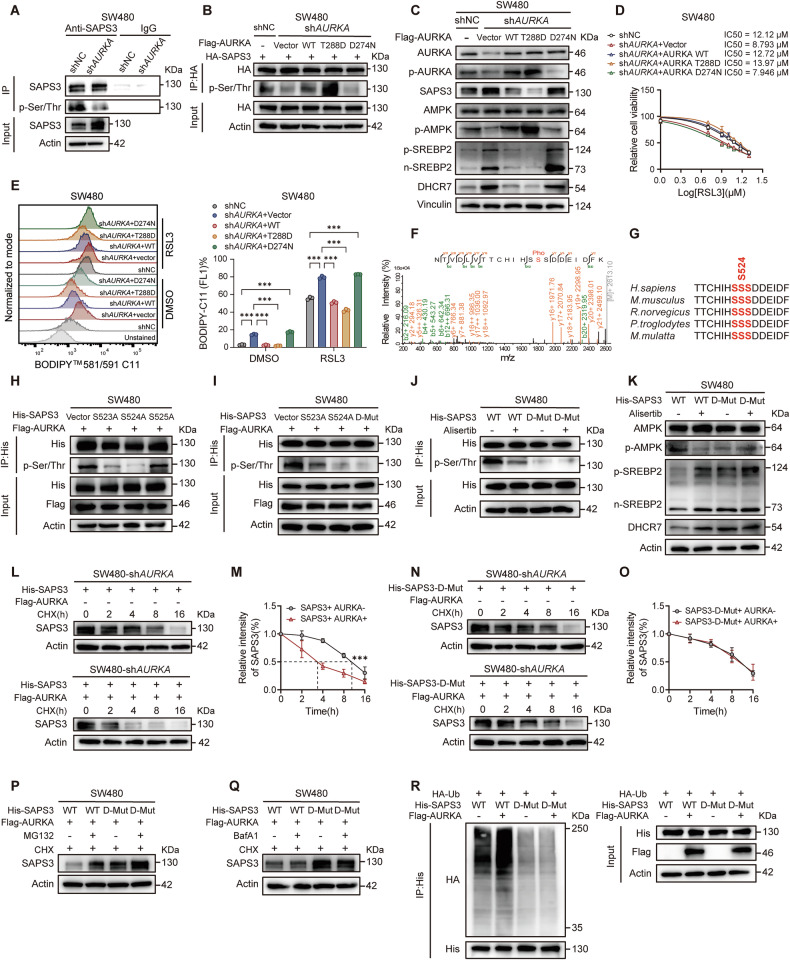
Phosphorylation of SAPS3 by AURKA modulates the AMPK-SREBP2-DHCR7 axis. **A** Co-IP analysis of SAPS3 indicating reduced phosphorylation levels in SW480-sh*AURKA* cells. **B** Co-IP analysis of the phosphorylation levels of SAPS3 in SW480-sh*AURKA* cells reconstituted with WT-, T288D-, or D274N-AURKA. **C** Western blot analysis of SAPS3, total AMPK, pAMPK, pSREBP2, nSREBP2, and DHCR7 in SW480-sh*AURKA* cells expressing WT-, T288D-, or D274N-AURKA. **D** IC_50_ analysis of RSL3 in SW480-sh*AURKA* cells expressing WT-, T288D-, or D274N-AURKA. **E** Representative FACS images and quantified values of lipid ROS levels in SW480-sh*AURKA* cells expressing WT-, T288D-, or D274N-AURKA. **F** LC-MS/MS identified Ser524 as AURKA-dependent phosphorylation sites in SAPS3. **G** Evolutionary conservation of SAPS3 S523/S524/Ser525 phosphorylation motifs across species (sequence alignment). **H**, **I** Co-IP analysis of phosphorylation levels for WT-, S523A-, S524A-, and S525A-SAPS3 mutants (**H**) or D-mut SAPS3 (**I**) in SW480 cells. **J** Co-IP analysis of WT or D-mut SAPS3 phosphorylation in SW480 cells treated with/without Alisertib (5 µM). **K** Western blot analysis of total AMPK, pAMPK, pSREBP2, nSREBP2, and DHCR7 levels in SW480 cells transfected with WT- or D-mut SAPS3, with/without Alisertib treatment. **L**, **N** Western blot analysis of WT- (**L**) or D-mut SAPS3 (**N**) protein stability at indicated timepoints after CHX (100 µg/ml) treatment in SW480-sh*AURKA* cells, comparing AURKA-reconstituted versus control conditions. **M**, **O** Quantified degradation kinetics (half-life) of WT- (**M**) or D-mut SAPS3 (**O**) from (**L**, **N**), respectively. **P** Western blot analysis of WT- or D-mut SAPS3 stability in CHX-treated SW480 cells, with/without MG132 (10 µM) under AURKA-expressing conditions. **Q** Western blot analysis of WT- or D-mut SAPS3 stability in CHX-treated SW480 cells, with/without BafA1 (200 ng/ml) under AURKA-expressing conditions. **R** Ubiquitination assays of WT- or D-mut SAPS3 in SW480 cells with/without AURKA expression. Data are presented as mean ± SD from at least three independent experiments. Statistical analyses were performed using unpaired Student’s *t* test (**E**) and two-way ANOVA test (**M**, **O**). ***p < 0.001.

Given that AURKA is a versatile serine/threonine kinase, we postulated that it might regulate SAPS3 protein stability through phosphorylation. Indeed, immunoprecipitated SAPS3 from AURKA-depleted CRC cells showed reduced serine/threonine phosphorylation level (Fig. 5A; Supplementary Fig. S5A). Reconstitution with wild-type AURKA (WT) restored SAPS3 phosphorylation in sh*AURKA* CRC cells, while a constitutively active AURKA mutant (T288D) further enhanced it. In contrast, a kinase-inactive AURKA variant (D274N) failed to restore SAPS3 phosphorylation, confirming SAPS3 as a substrate of AURKA (Fig. 5B; Supplementary Fig. S5B). In addition, downstream signaling events mediated by SAPS3, including AMPK activation, SREBP2 nuclear translocation, and DHCR7 expression, were similarly regulated by WT and mutant AURKA constructs in sh*AURKA* CRC cells (Fig. 5C; Supplementary Fig. S5C). More importantly, both WT and T288D AURKA reversed the ferroptosis hypersensitivity induced by AURKA depletion, whereas D274N failed to do so, suggesting that AURKA suppresses ferroptosis through its kinase activity (Fig. 5D, E; Supplementary Fig. S5D, E). To identify AURKA-mediated phosphorylation sites on SAPS3, we performed mass spectrometry and detected phosphorylation at Ser 524 (Fig. 5F). We constructed phospho-deficient SAPS3 mutants by substituting the consecutive serine (S) residues (S523, S524, and S525) with alanine (A). Mutation of S523 or S524, but not S525, significantly impaired AURKA-mediated phosphorylation (Fig. 5G, H; Supplementary Fig. S5F). We then generated a double mutant (D-mut) harboring S523A and S524A, which exhibited an even lower phosphorylation than the single mutants (Fig. 5I; Supplementary Fig. S5G). Further, in vitro kinase assays using purified recombinant proteins demonstrated that AURKA directly phosphorylates SAPS3 at S523/S524, supporting a direct kinase-substrate interaction (Supplementary Fig. S5H). Inhibiting AURKA with Alisertib reduced the phosphorylation of WT SAPS3 but had a negligible effect on the D-mut variant (Fig. 5J; Supplementary Fig. S5I). Importantly, D-mut SAPS3 failed to modulate the AMPK-SREBP2-DHCR7 axis and remained unresponsive to Alisertib treatment (Fig. 5K; Supplementary Fig. S5J). In CHX chase assays, both single mutants (S523A or S524A) and D-mut SAPS3 counteracted AURKA-induced degradation, with the D-mut variant displaying a markedly extended half-life compared to WT SAPS3 (Fig. 5L–O; Supplementary Fig. S5K–P). This degradation was rescued by the proteasome inhibitor MG132 but not by the autophagy inhibitor BafA1, indicating dependence on the ubiquitin-proteasome system (UPS) (Fig. 5P, Q; Supplementary Fig. S5Q, R). Accordingly, ubiquitination assays showed that AURKA enhanced ubiquitination of WT but not D-mut SAPS3 (Fig. 5R). Together, these results demonstrate that AURKA phosphorylates SAPS3 at Ser523 and Ser524 to expedite its UPS-dependent degradation.

### Targeting AURKA sensitizes CRC cells to chemotherapy

**Fig. 6:**
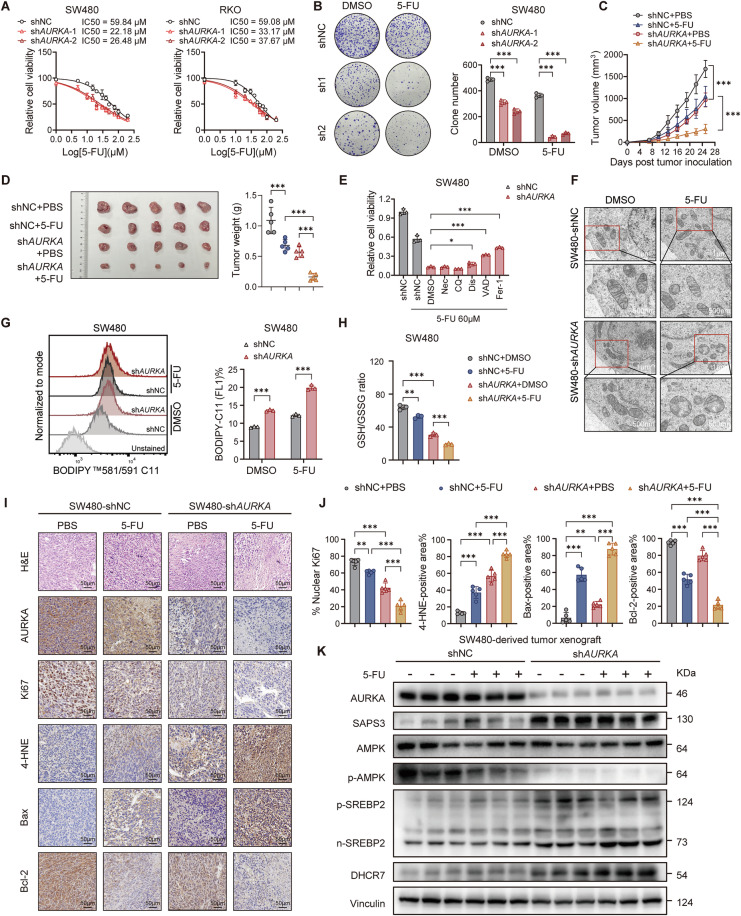
AURKA knockdown renders CRC cells vulnerable to chemotherapy. **A** Dose-response analysis of 5-FU sensitivity in CRC (shNC and sh*AURKA*) cells treated for 48 h. **B** Representative images (left) and quantification (right) of clonogenic assay with 500 SW480 cells/well treated with 5-FU (60 μM). **C**, **D** Tumor growth curves (**C**) and representative images of excised tumors with their weights at endpoint (**D**) in SW480 (shNC and sh*AURKA*) implanted nude mice, followed by *i.p*. injection of 5-FU (25 mg/kg) or PBS (*n* = 5 mice per group). **E** Viability of SW480 (shNC and sh*AURKA*) cells with or without 5-FU (60 μM) for 48 h in combination with Necrostatin (Nec, 20 μM), Chloroquine (CQ, 30 μM), Disulfiram (Dis, 1 μM), Z-VAD-FMK (VAD, 25 μM) and Ferrostatin-1 (Fer, 10 μM). **F** Representative TEM images of SW480 (shNC and sh*AURKA*) cells after 5-FU (60 μM) treatment for 24 h. **G**, **H** Representative FACS images and quantified lipid ROS levels (**G**) and GSH/GSSG ratios (**H**) in SW480 (shNC and sh*AURKA*) cells treated with 5-FU (60 μM, 24 h). **I**, **J** Representative H&E and IHC staining images (**I**) and quantification (**J**) of Ki67, 4-HNE, Bax, and Bcl-2 staining in xenograft tumor sections. **K** Western blot analysis of AURKA, SAPS3, total AMPK, pAMPK, pSREBP2, nSREBP2, and DHCR7 in SW480 xenograft tumors with or without 5-FU treatment. Data are presented as mean ± SD from at least three independent experiments. Statistical analyses were performed using unpaired Student’s *t* test (**B**, **D**, **E**, **G**, **H**, **J**) and two-way ANOVA test (**C**). **p < 0.01, ***p < 0.001.

Ferroptosis augmentation in combination with chemotherapy has emerged as a promising strategy to overcome cancer resistance [7, 43, 44]. We thus hypothesized that targeting AURKA could enhance the efficacy of chemotherapeutic agents in CRC. As expected, AURKA knockdown significantly sensitized CRC cells to 5-FU and oxaliplatin, markedly reducing cell viability (Fig. 6A; Supplementary Fig. S6A) and impairing colony formation (Fig. 6B; Supplementary Fig. S6B). This chemosensitization effect was further validated in vivo, where AURKA depletion synergized with 5-FU treatment to suppress tumor growth and reduced xenograft tumor weights, achieving the strongest suppression of tumorigenicity (Fig. 6C, D). Further, administration of the ferroptosis inhibitor ferrostatin-1 (Fer-1) and the apoptotic inhibitor Z-VAD-FMK (VAD), but not inhibitors of other cell death modalities (including necroptosis [Nec], autophagy [CQ], and pyroptosis [Dis]), reversed the viability decline of 5-FU-treated sh*AURKA* CRC cells (Fig. 6E), suggesting that targeting AURKA potentiates the cytotoxic effects of 5-FU primarily through ferroptosis and apoptosis. Notably, combining either (VAD+Fer-1) or (VAD + 7-DHC) led to near-complete recovery of cell viability, indicating that the pro-apoptotic effect of AURKA knockdown acts independently of the 7-DHC-mediated ferroptotic pathway (Supplementary Fig. S6D). In line with this, AURKA-deficient CRC cells exhibited pronounced ferroptotic features following 5-FU treatment, including exacerbated mitochondrial alterations (Fig. 6F), increased lipid ROS (Fig. 6G), and reduced GSH/GSSG ratios (Fig. 6H; Supplementary Fig. S6E), along with elevated apoptotic markers (Supplementary Fig. S6F–H). In vivo, AURKA-deficient xenograft tumors showed decreased proliferation, enhanced necrosis, elevated lipid ROS, and higher apoptotic indexes post 5-FU treatment, compared to control tumors (Fig. 6I, J). Molecular profiling revealed that AURKA depletion upregulated SAPS3, promoted AMPK dephosphorylation, and induced SREBP2 nuclear accumulation with concomitant DHCR7 overexpression in xenografts (Fig. 6K). Collectively, these findings establish that targeting AURKA sensitizes CRC to chemotherapy by inducing ferroptosis and apoptosis.

### AURKA inhibitor augments the benefit of 5-FU in CRC

**Fig. 7:**
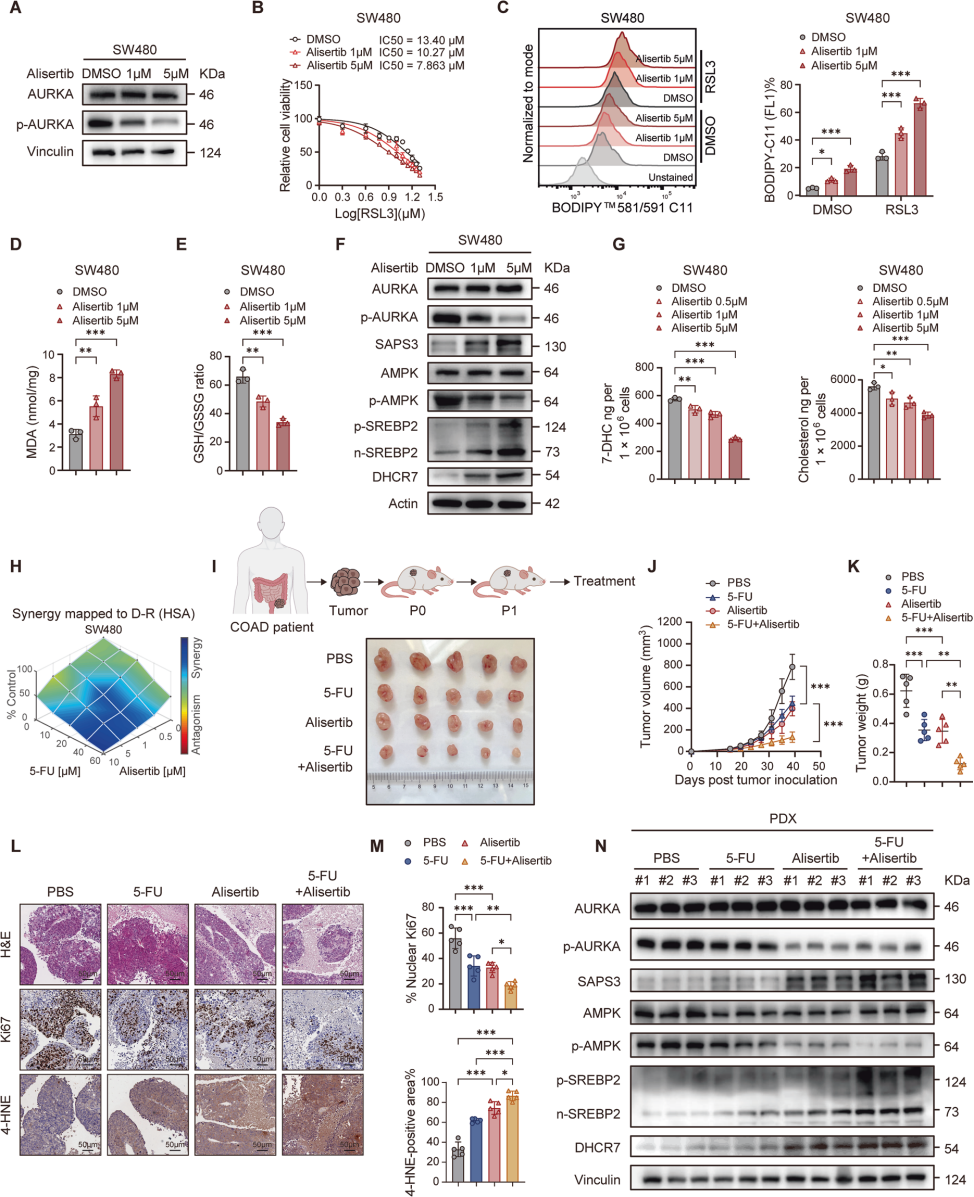
AURKA inhibitor enhances the therapeutic efficacy of 5-FU in CRC. **A** Western blot analysis of pAURKA in SW480 cells treated with Alisertib. **B** RSL3-IC_50_ analysis in SW480 cells after Alisertib treatment (1 µM & 5 µM). **C**-**E** Representative FACS plots and quantified lipid ROS levels (**C**), MDA levels (**D**), and GSH/GSSG ratios (**E**) in SW480 cells treated with RSL3 (5 µM) and Alisertib. **F** Western blot analysis of AURKA, pAURKA, SAPS3, AMPK, pAMPK, pSREBP2, nSREBP2, and DHCR7 in Alisertib-treated SW480 cells. **G** LC-MS/MS quantification of 7-DHC and cholesterol levels in SW480 cells post Alisertib treatment. **H** Clonogenic assay showing synergistic suppression of SW480 survival with Alisertib and 5-FU (Combenefit synergy analysis). **I**, **K** Excised tumor images (**I**), tumor growth curves (**J**) and endpoint tumor weights (**K**) from PDX model after 5-FU (25 mg/kg, i.p.) and/or Alisertib (30 mg/kg, oral gavage) (n = 5 mice per group). **L,** **M** Representative H&E and IHC staining images (**L**) and quantification (**M**) of Ki67 and 4-HNE staining in PDX tumor sections. **N** Western blot analysis of AURKA, pAURKA, SAPS3, AMPK, pAMPK, pSREBP2, nSREBP2, and DHCR7 in Alisertib and/or 5-FU-treated PDX tumors. Data are presented as mean ± SD from at least three independent experiments. Statistical analyses were performed using unpaired Student’s *t* test (**C**, **D**, **E**, **G**, **K**, **M**) and two-way ANOVA test (**J**). *p < 0.05, **p < 0.01, ***p < 0.001.

To evaluate the therapeutic potential of AURKA inhibition, we treated CRC cells with Alisertib (MLN8237), a clinically validated and highly selective AURKA inhibitor. Consistent with genetic depletion, pharmacological inhibition of AURKA efficiently sensitized CRC cells to ferroptosis (Fig. 7A, B; Supplementary Fig. S7A, B), as manifested by elevated levels of lipid ROS and intracellular MDA, along with reduced GSH/GSSG ratios (Fig. 7C–E; Supplementary Fig. S7C–E). Alisertib treatment also recapitulated the molecular changes observed in AURKA-depleted cells, including modulation of the SAPS3-AMPK-SREBP2-DHCR7 axis in a dose-dependent manner (Fig. 7F; Supplementary Fig. S7F). More importantly, Alisertib elicited a dose-responsive decrease in intracellular 7-DHC levels, while cholesterol levels declined less markedly, confirming AURKA’s regulatory role in cholesterol metabolism (Fig. 7G). To determine whether Alisertib could not only induce cell death alone but also synergize with chemotherapy, we co-treated CRC cells with Alisertib and 5-FU. The combination significantly inhibited cell viability compared to either agent alone (Fig. 7H). To better simulate the physiological tumor microenvironment, we established a CRC patient-derived xenograft (PDX) model. As expected, monotherapy with Alisertib or 5-FU reduced tumor growth and xenograft weights, but their combination produced a synergistic antitumor effect (Fig. 7I, J). H&E and IHC analyses revealed that the combination therapy achieved the greatest reduction in cell proliferation (Ki67 staining) and the highest accumulation of lipid ROS (4-HNE staining) (Fig. 7K). Furthermore, expression patterns of the SAPS3-AMPK-SREBP2-DHCR7 axis in Alisertib-treated PDX tumors closely mirrored the changes observed in CRC cells (Fig. 7L). Taken together, the above results underscore the synergistic antitumor potential of Alisertib in Dcombination with 5-FU in vivo, acting through modulation of ferroptosis and cholesterol metabolism.

### High AURKA expression correlates with chemoresistance in CRC patients undergoing chemotherapy

**Fig. 8:**
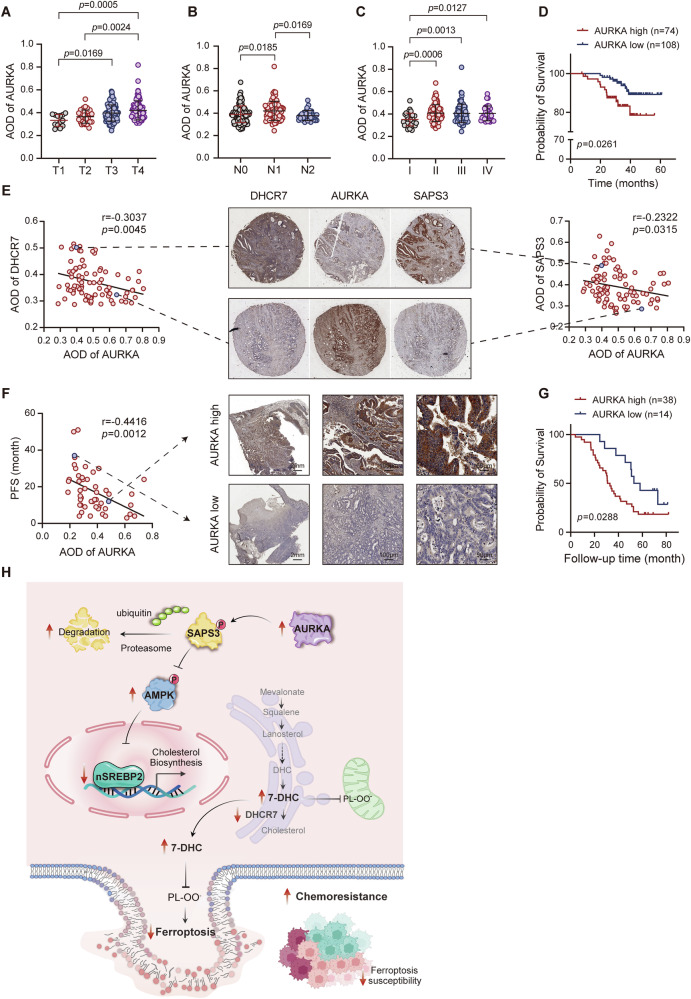
Elevated AURKA levels correlate with poor chemotherapy response in CRC patients. **A**–**C** IHC analysis of AURKA protein expression in CRC TMAs stratified by Tumor stages (**A**), lymph node metastases (**B**), and NCCN stages (**C**). **D** Kaplan-Meier overall survival (OS) curves comparing patients with low versus high AURKA expression (*n* = 182). **E** Correlation analysis of IHC scores between AURKA and SAPS3 or DHCR7 in tumor specimens (*n* = 86). **F**, **G** Pearson correlation analysis of AURKA expression with PFS (**F**), and OS (**G**) in CRC patients undergone chemotherapy (*n* = 52). **H** Schematic diagram illustrating how upregulated AURKA suppresses ferroptosis susceptibility and enhances chemoresistance in colorectal cancer. Data are presented as mean ± SD from at least three independent experiments. Statistical analyses were performed using unpaired Student’s *t* test (**A**–**C**), log-rank test (**D,** **G**).

To assess the clinical relevance of our findings, we analyzed AURKA protein expression in CRC-TMAs. Elevated AURKA levels were significantly associated with advanced tumor stages and lymph node metastasis (Fig. 8A–C). Moreover, high AURKA expression correlated with reduced overall survival (OS) in CRC patients, underscoring its prognostic significance (Fig. 8D). Consistent with observations in AURKA-depleted CRC cells and AURKA-inhibited PDX models, human CRC samples exhibited a strong inverse correlation between AURKA and the expression of SAPS3 and DHCR7 (Fig. 8E). To validate the clinical correlation between AURKA expression and chemotherapy response, we evaluated a cohort of stage II/III CRC patients who underwent curative resection followed by adjuvant chemotherapy (FOLFOX or XELOX regimens, Supplementary Table S2). Notably, patients with high AURKA expression showed significantly shorter progression-free survival (PFS) (Fig. 8F) and worse OS compared to those with low AURKA expression (Fig. 8G). These results reinforce the clinical relevance of AURKA as a biomarker for chemotherapy responsiveness in CRC and provide translational support for targeting the AURKA-SAPS3-cholesterol biosynthesis axis.

## Discussion

Our work characterizes an unforeseen role of AURKA in orchestrating cholesterol metabolism and ferroptosis resistance. Acting through a kinase-dependent mechanism, AURKA phosphorylates and destabilizes SAPS3, the regulatory subunit of its antagonistic phosphatase PP6, thereby modulating the AMPK-SREBP2-DHCR7 signaling cascade. This regulatory hierarchy facilitates the accumulation of 7-DHC, an anti-ferroptotic metabolite, augmenting chemoresistance (Fig. 8H). This study not only unveils a yet-unrecognized, cell-intrinsic anti-ferroptotic function of AURKA co-opted by cancer cells to evade therapy, shedding light on new chemosensitization strategies, but also refines our understanding of AURKA-evoked metabolic plasticity in tuning tumorigenesis and therapeutic adaptation.

While AURKA has been implicated in ferroptosis suppression in other tumor contexts, including KEAP1-NRF2 activation in meningioma [45], the NPM1/YAP1 axis in Ewing’s sarcoma [46], and redox regulation in breast cancer nanotherapies [47], our work uniquely connects AURKA to cholesterol biosynthesis. Although previous reports have noted associations between AURKA and lipid peroxidation markers (GPX4, SLC7A11, ALOX5, and ACSL4) [48, 49], our study provide the first mechanistic evidence that AURKA promotes ferroptosis resistance by sustaining 7-DHC levels through SREBP2-driven transcriptional control. SREBP2, a master regulator of cholesterol homeostasis, is synthesized as an inactive precursor and activated in the Golgi, after which its nuclear form induces the transcription of mevalonate pathway genes such as HMGCR, SQS, and DHCR24 [50]. A recent study reported that AURKB exacerbated resistance to chemotherapy and immunotherapy through NCEH1-driven intratumoral cholesterol immunosuppression in cholangiocarcinoma [51]. In contrast, our study identifies AURKA as a key regulator of 7-DHC biosynthesis through the AMPK-SREBP2-DHCR7 axis, linking it to ferroptosis evasion in CRC. While AURKB and AURKA act through distinct metabolic effectors, both kinases converge on cholesterol-related pathways that impact tumor progression and therapeutic response. These findings highlight the broader role of Aurora kinases in cholesterol homeostasis and underscore the potential of targeting this kinase family to overcome treatment resistance. Given the immunomodulatory effects of cholesterol, further investigation into AURKA’s role in immune evasion and response to therapy in immunocompetent models is warranted. We found that AURKA deficiency facilitated SREBP2 nuclear translocation and transcriptional activation, accompanied by reduced AMPK phosphorylation, implying that AURKA sustains AMPK activity to restrict SREBP2-driven cholesterol biosynthesis. Notably, this regulatory relationship appears context-specific, as in non-small cell lung cancer (NSCLC) cells, AURKA was reported to suppress AMPK phosphorylation [29], highlighting a tumor-type-dependent divergence in AURKA-AMPK axis regulation.

At the mechanistic core, we demonstrate that AURKA directly phosphorylates the PP6 regulatory subunit SAPS3 (PPP6R3) at Ser523/524, promoting its proteasomal degradation. SAPS3 defines PP6’s substrate specificity [42], and given that PP6 dephosphorylates AURKA to constrain its mitotic activity [52–54], this phosphorylation event establishes a reciprocal regulatory circuit. In this scenario, amplified AURKA destabilizes SAPS3, impairs PP6 function, thereby sustaining AURKA signaling and metabolic reprogramming. Our integrated proteomic and functional assays designate SAPS3 as a novel substrate of AURKA, revealing a feedforward mechanism that couples mitotic control with ferroptosis resistance via cholesterol metabolism.

Our findings also highlight a therapeutically actionable link between AURKA inhibition, ferroptosis sensitization, and enhanced chemotherapy response in CRC. Alisertib, a selective AURKA inhibitor, recapitulated the ferroptosis-sensitizing effects of AURKA knockdown and showed marked synergy with 5-FU in both CRC cells and patient-derived xenografts. These data support the rational design of combination regimens pairing AURKA inhibitors with standard chemotherapy to overcome resistance. While Alisertib has demonstrated clinical benefit in hematologic malignancies [55], its efficacy in solid tumors has been limited [56]. Our results provide a mechanistic rationale for repositioning AURKA inhibition in solid tumors, particularly CRC, as part of a ferroptosis-inducing therapeutic strategy.

In summary, this study establishes AURKA as a central node linking ferroptosis suppression, cholesterol metabolism, and chemoresistance in CRC. By mechanistically defining this pathway, we identify AURKA as both a biomarker for ferroptosis vulnerability and a promising therapeutic target to enhance chemotherapy efficacy. These insights underscore the potential of integrating metabolic reprogramming with ferroptosis modulation to improve treatment outcomes in solid tumors.

## Supplementary information

Supplementary Figure 1

Supplementary Figure 2

Supplementary Figure 3

Supplementary Figure 4

Supplementary Figure 5

Supplementary Figure 6

Supplementary Figure 7

Supplementary Figure legends

Supplementary Tables

Uncropped Western Blot Image

## Data Availability

All data needed to evaluate the conclusions in the paper are present in the paper and/or the Supplemental materials. Raw data are available upon request from the corresponding author. RNA-seq data have been deposited in the Gene Expression Omnibus with accession code GSE302491.
