# Supplementary figures and images for: AURKA-mediated destabilization of SAPS3 drives ferroptosis evasion via 7-dehydrocholesterol biosynthesis in colorectal cancer
Source: Cell Death Dis. 2026 Mar 16;17(1):361. doi: 10.1038/s41419-026-08549-9 (PMC13039981; doi:10.1038/s41419-026-08549-9)

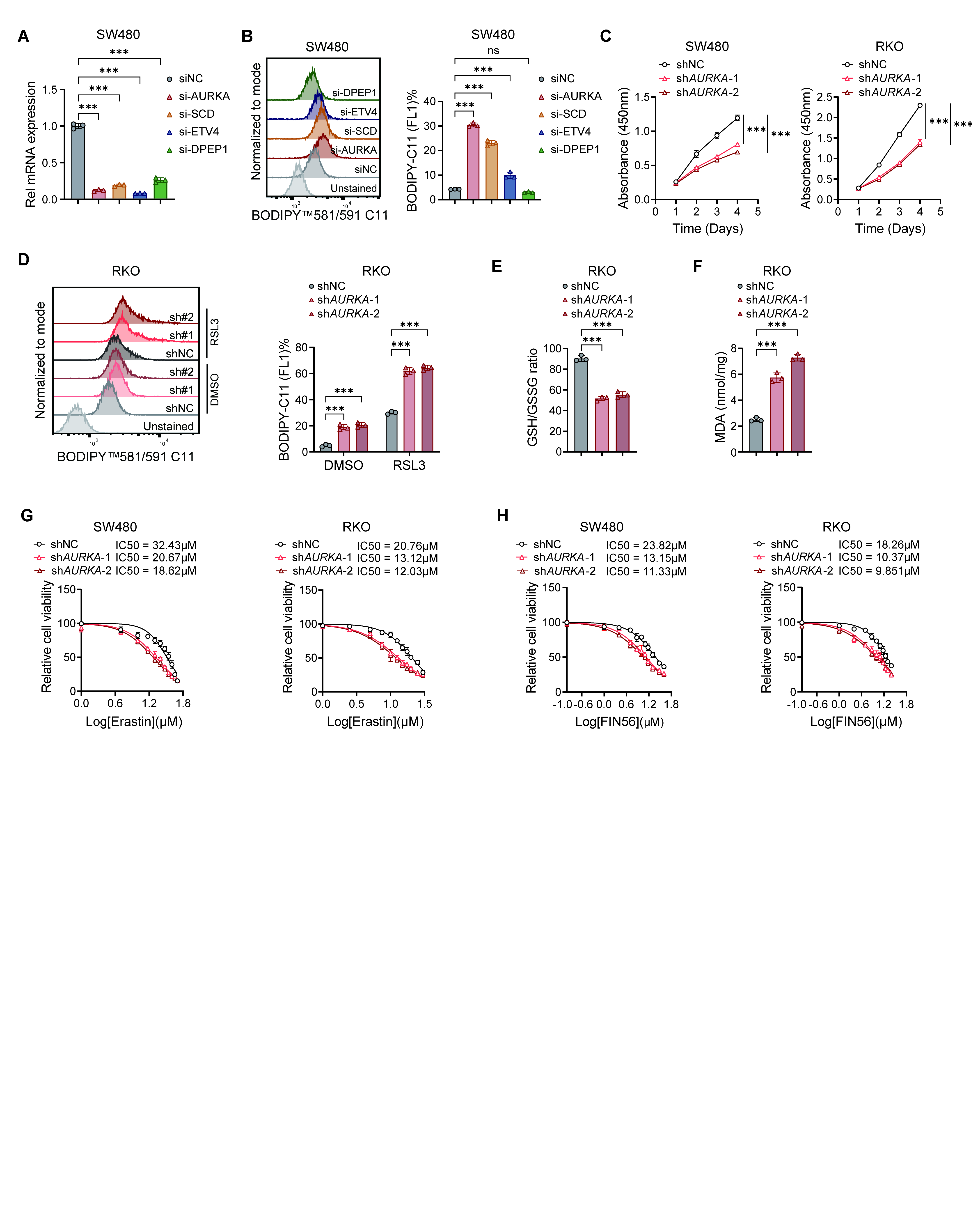

Supplement: Supplementary file 1 — Supplementary Figure 1 [file 41419_2026_8549_MOESM1_ESM.tif]

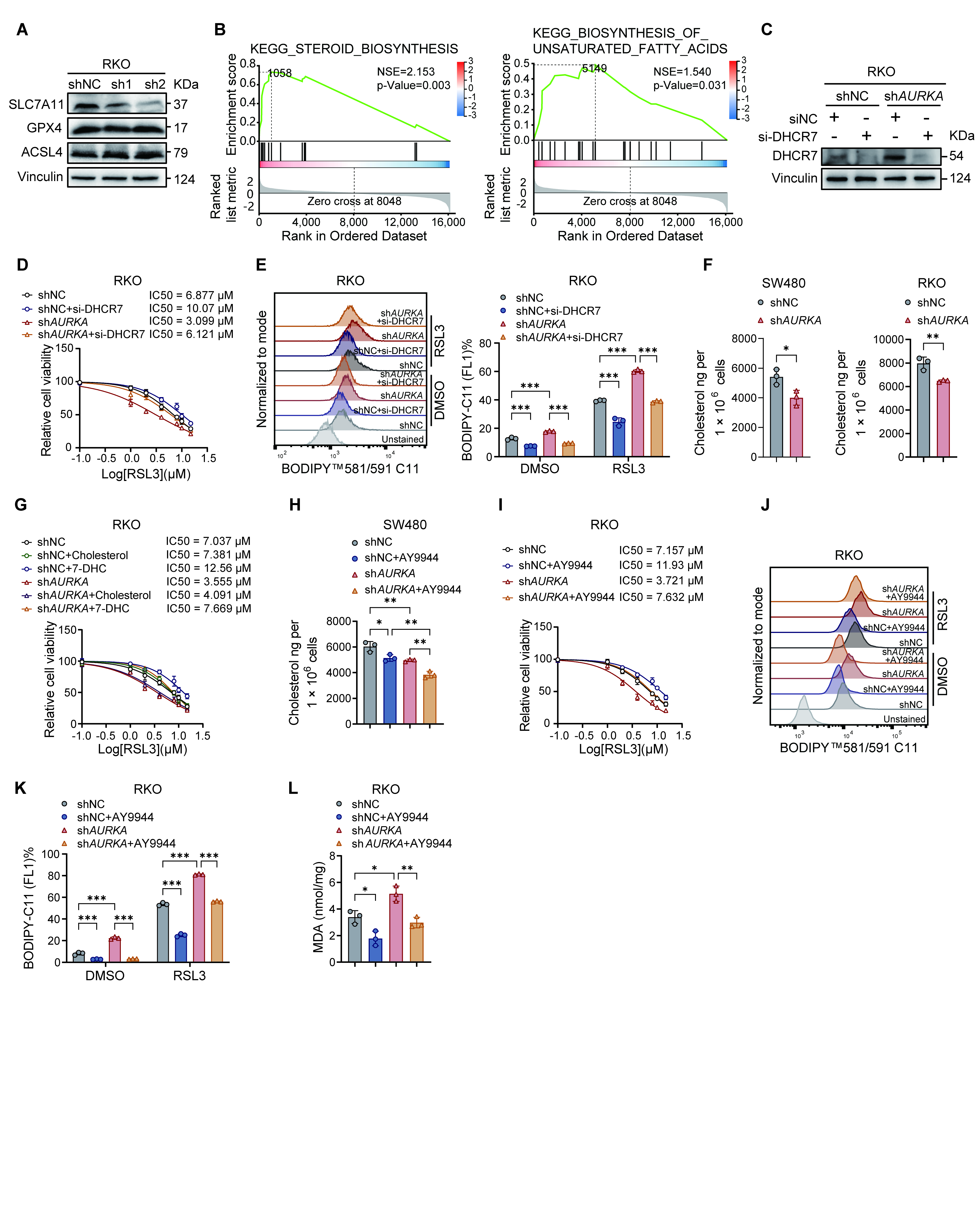

Supplement: Supplementary file 2 — Supplementary Figure 2 [file 41419_2026_8549_MOESM2_ESM.tif]

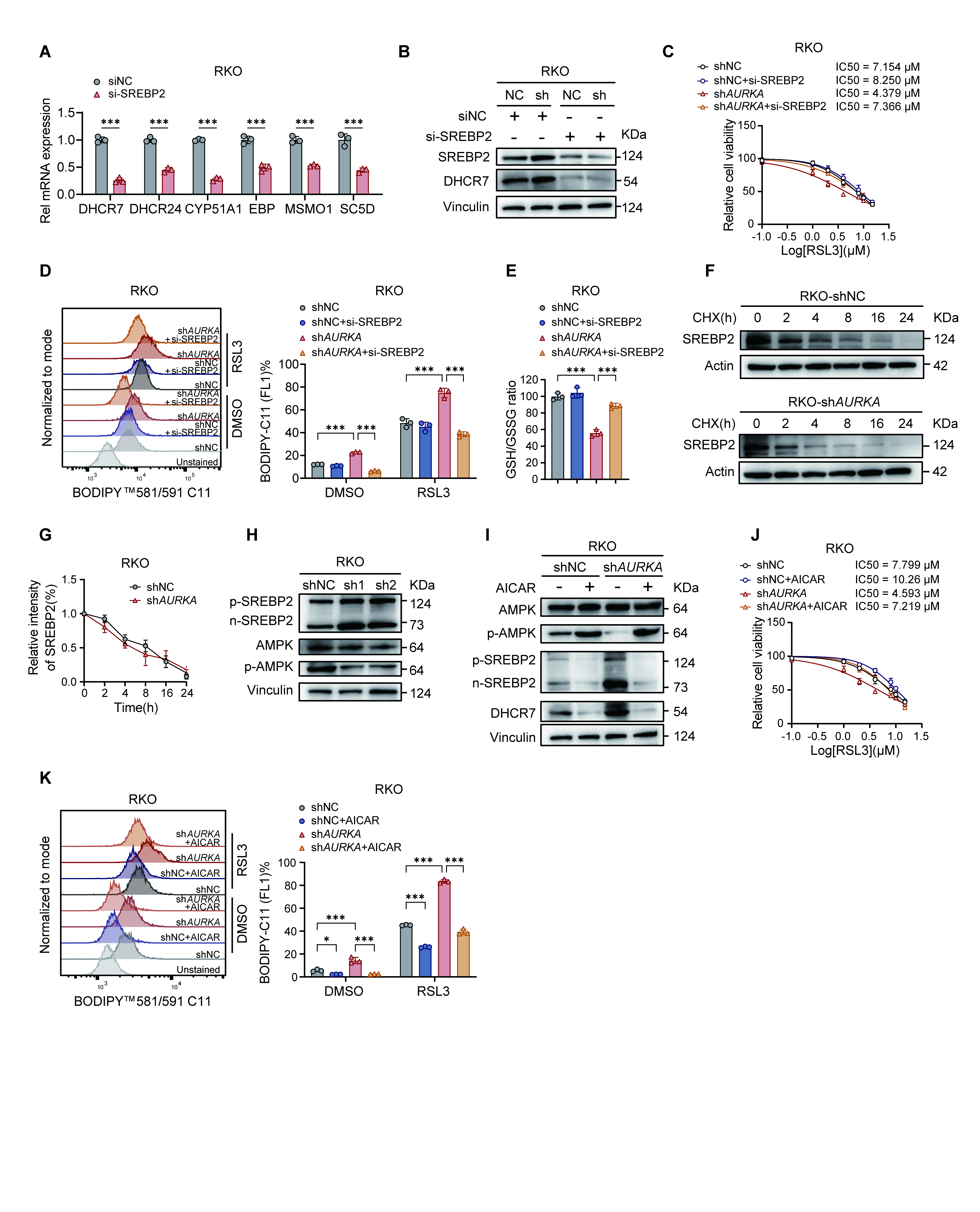

Supplement: Supplementary file 3 — Supplementary Figure 3 [file 41419_2026_8549_MOESM3_ESM.tif]

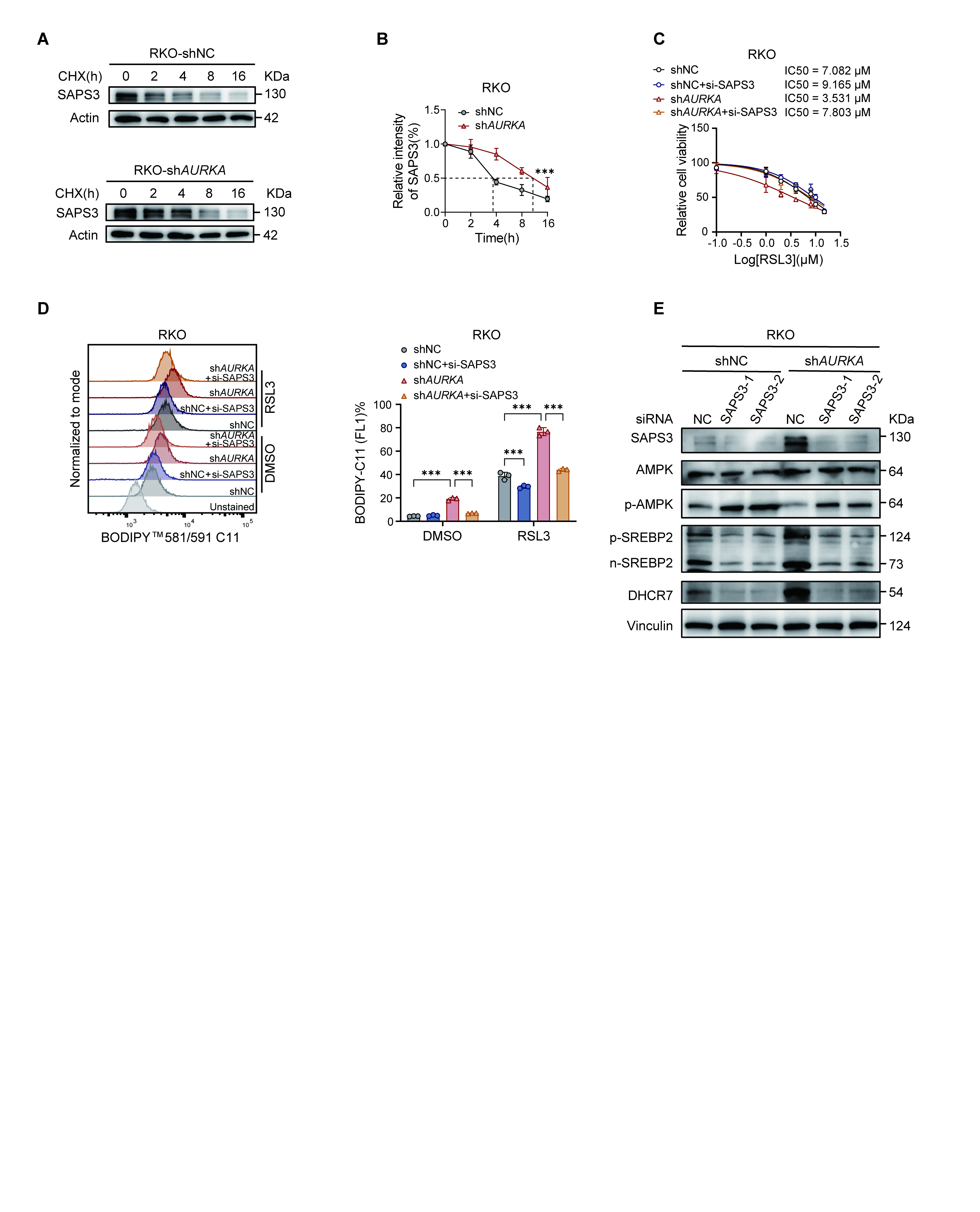

Supplement: Supplementary file 4 — Supplementary Figure 4 [file 41419_2026_8549_MOESM4_ESM.tif]

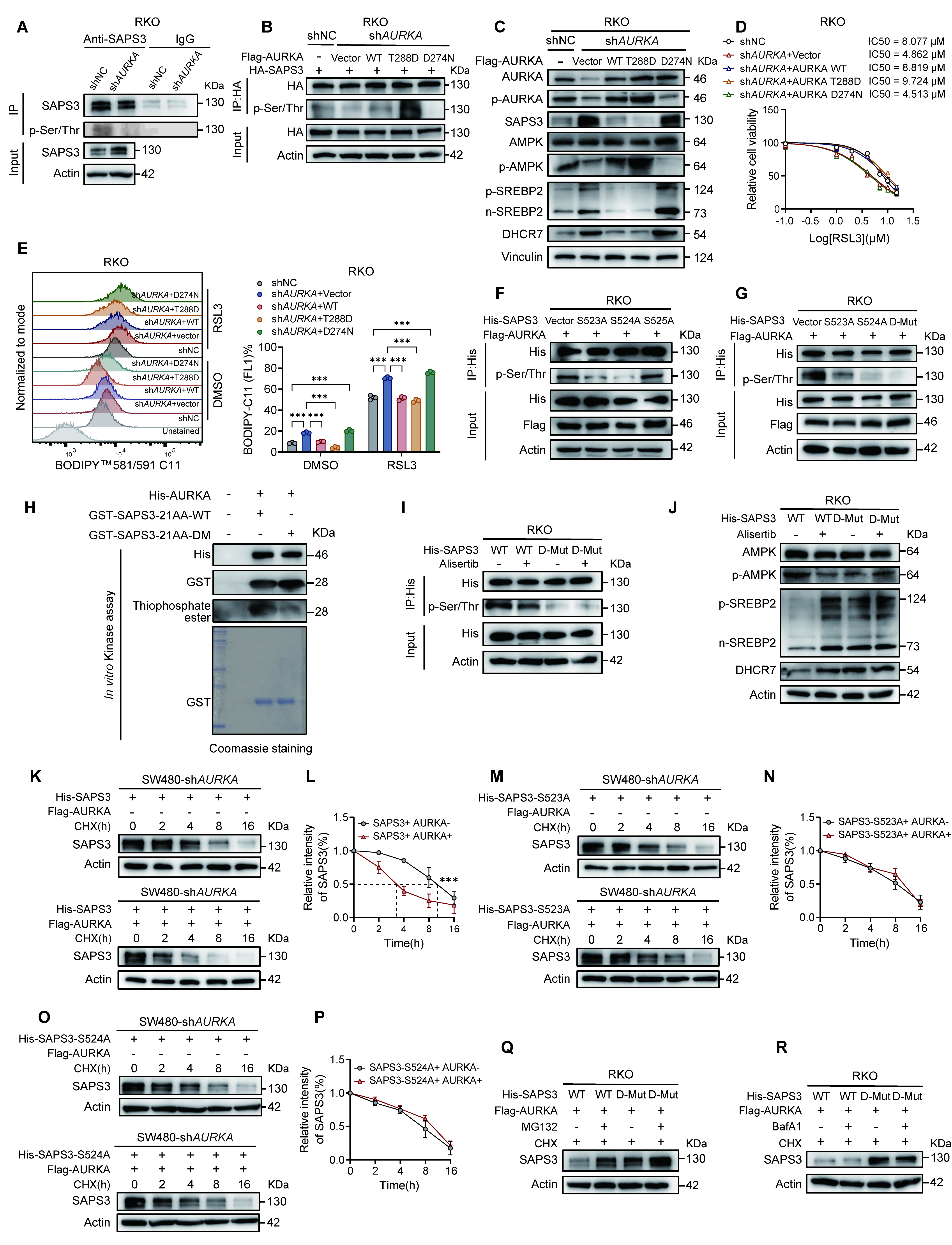

Supplement: Supplementary file 5 — Supplementary Figure 5 [file 41419_2026_8549_MOESM5_ESM.tif]

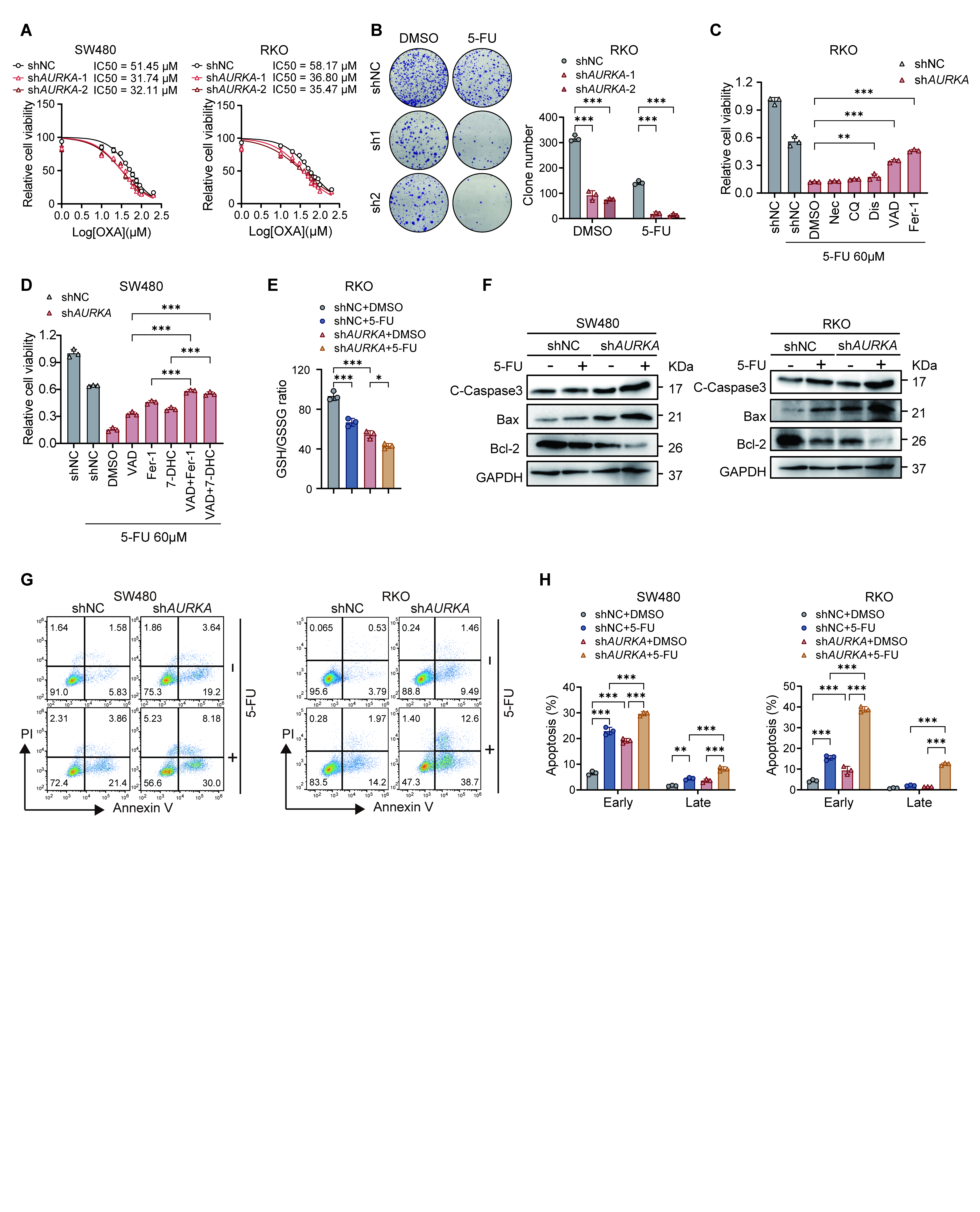

Supplement: Supplementary file 6 — Supplementary Figure 6 [file 41419_2026_8549_MOESM6_ESM.tif]

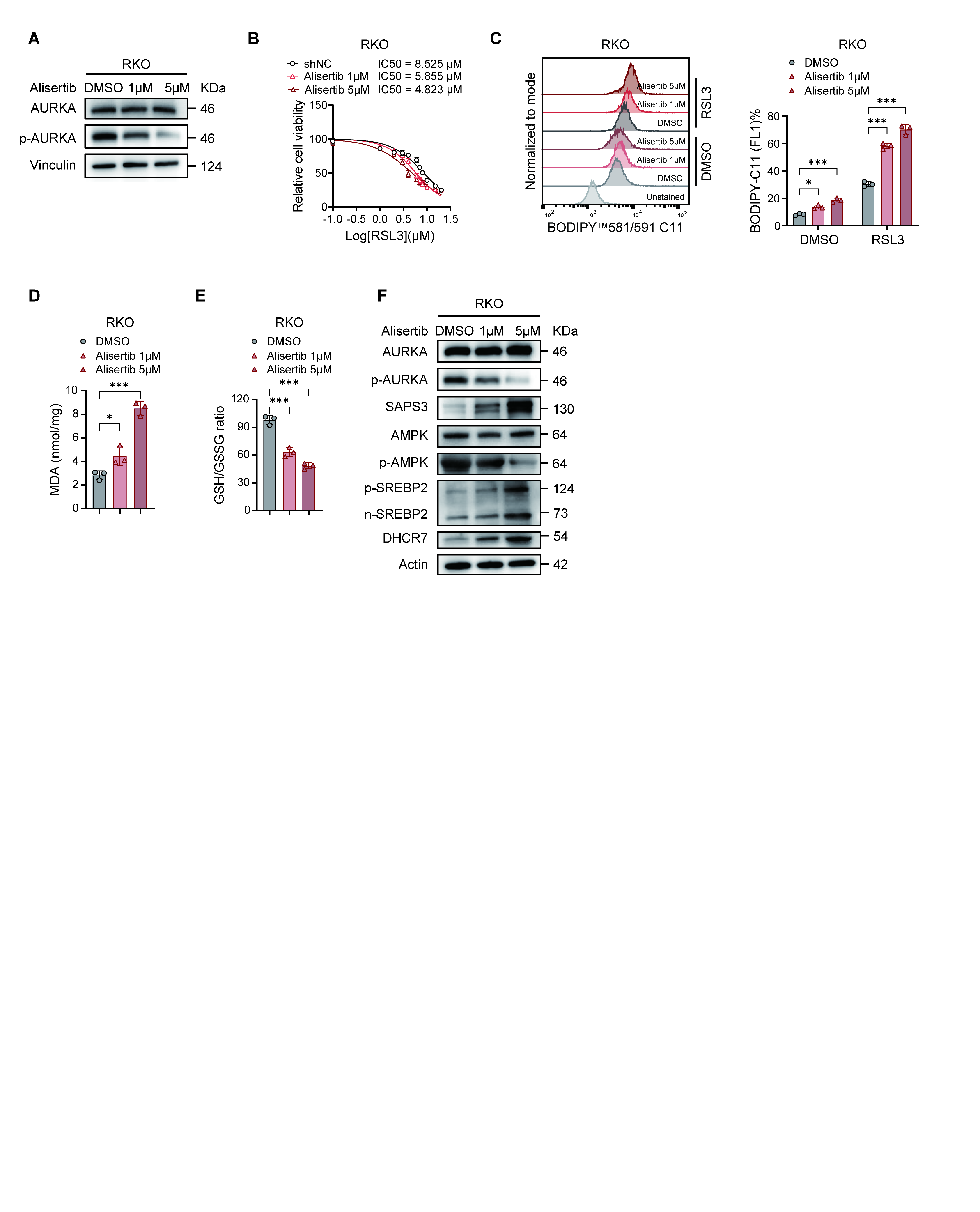

Supplement: Supplementary file 7 — Supplementary Figure 7 [file 41419_2026_8549_MOESM7_ESM.tif]
