# Supplementary material for: AURKA-mediated destabilization of SAPS3 drives ferroptosis evasion via 7-dehydrocholesterol biosynthesis in colorectal cancer
Source: Cell Death Dis. 2026 Mar 16;17(1):361. doi: 10.1038/s41419-026-08549-9 (PMC13039981; doi:10.1038/s41419-026-08549-9)
Supplement: Supplementary file 8 — Supplementary Figure legends [file 41419_2026_8549_MOESM8_ESM.docx]

**Supplementary Figure Legends**

**Supplementary Figure 1. AURKA is a ferroptosis suppressor in CRC. A,** qPCR analysis of siRNA knockdown efficiency for AURKA, SCD, ETV4, and DPEP1 in SW480 cells. **B,** Representative FACS images and quantified values of lipid ROS levels in SW480 cells post-knockdown. **C,** CCK-8 assays comparing viability of SW480 (shNC versus sh*AURKA*) cells. **D,** Representative FACS images and quantified values of the lipid ROS levels in RKO (shNC and sh*AURKA*) cells treated with RSL3 (2.5 μM) for 24 h. **E** and **F,** Intracellular GSH/GSSG ratios (**E**) and cellular concentrations of MDA (**F**) in RKO (shNC and sh*AURKA*) cells. **G** and **H,** IC_50_ analysis of erastin (**G**) or FIN56 (**H**) in CRC (shNC and sh*AURKA*) cells treated with different concentrations of ferroptotic inducers for 48 h. Data are presented as mean ± SD from at least three independent experiments. Statistical analyses were performed using unpaired Student’s *t* test [(A), (B), (D), (E), and (F)] and two-way ANOVA test (C). ****p* < 0.001.

**Supplementary Figure 2. AURKA suppresses ferroptosis** **by inhibiting DHCR7-catalyzed 7-DHC conversion. A,** Western blot analysis of SLC7A11, GPX4, and ACSL4 in RKO (shNC and sh*AURKA*) cells. **B,** Gene set enrichment analysis (GSEA) showing significant enrichment of Gene Ontology terms in SW480-sh*AURKA* cells. **C,** Western blot analysis of DHCR7 protein levels in RKO cells after siRNA-mediated knockdown. **D,** IC_50_ analysis of RSL3 in RKO (shNC and sh*AURKA*) cells treated with RSL3 (48 h) following DHCR7 silence. **E,** Representative FACS images and quantified values of lipid ROS levels in RKO (shNC and sh*AURKA*) cells treated with RSL3 (2.5 μM, 24 h) post DHCR7 silencing. **F,** LC-MS/MS quantification of cholesterol levels in CRC (shNC and sh*AURKA*) cells. **G,** Quantification of RSL3 IC_50_ values in RKO (shNC and sh*AURKA*) cells after pretreatment with 7-DHC (25 μM), cholesterol (25 μM) for 24 h. **H,** Intracellular levels of cholesterol in SW480 (shNC and sh*AURKA*) cells treated with AY9944 (100 nM) for 24 h. **I,** Quantification of RSL3 IC_50_ values in RKO (shNC and sh*AURKA*) cells after pretreatment with AY9944 (100 nM) for 24 h. **J**-**L,** Quantification of intracellular lipid ROS (**K**) and MDA (**L**) levels in RKO (shNC and sh*AURKA*) cells treated with AY9944 (100 nM) for 24 h. Data are presented as mean ± SD from at least three independent experiments. Statistical analyses were performed using unpaired Student’s *t* test [(E), (F), (H), (K), and (L)]. **p* < 0.05, ***p* < 0.01, ****p* < 0.001.

**Supplementary Figure 3. AURKA downregulates DHCR7 expression through the AMPK-SREBP2 pathway. A,** qPCR quantification of cholesterol biosynthesis enzyme mRNA levels in SREBP2-silenced RKO cells. **B**-**E,** Knockdown of SREBP2 in RKO cells with siRNA transfection. Western blot analysis of SREBP2 and DHCR7 protein expression (**B**). RSL3 sensitivity (IC_50_) of RKO (shNC and sh*AURKA*) cells (**C**), representative FACS images with quantified lipid ROS levels (**D**), and GSH/GSSG ratios (**E**) following RSL3 treatment (24 h). **F** and **G,** Western blot analysis of SREBP2 at indicated timepoints after CHX (100µg/ml) treatment (**F**), and quantified degradation kinetics (**G**) in RKO cells. **H** and **I,** Western blot analysis of pSREBP2, nSREBP2, total AMPK, and pAMPK levels in RKO (shNC and sh*AURKA*) cells (**H**) and post AICAR treatment (1 mM, 24 h, **I**). **J,** IC_50_ analysis of RSL3 in RKO (shNC and sh*AURKA*) cells after pretreatment with AICAR (1 mM, 24 h). **K,** Representative FACS images and quantified values of the lipid ROS levels in RKO (shNC and sh*AURKA*) cells treated with AICAR (1 mM, 24 h). Data are presented as mean ± SD from at least three independent experiments. Statistical analyses were performed using unpaired Student’s *t* test [(A), (D), (E), and (K)]. **p* < 0.05, ****p* < 0.001.

**Supplementary Figure 4. AURKA inhibits ferroptosis via its interaction with SAPS3. A** and **B,** Western blot analysis of SAPS3 at indicated timepoints after CHX (100µg/ml) treatment (**A**), and quantified degradation kinetics (**B**) in RKO cells. **C,** IC_50_ analysis of RSL3 in RKO (shNC and sh*AURKA*) cells treated with RSL3 (48 h) following SAPS3 knockdown. **D,** Representative FACS images and quantified values of lipid ROS levels in RKO (shNC and sh*AURKA*) cells after SAPS3 silencing. **E,** Western blot analysis of total AMPK, pAMPK, pSREBP2, nSREBP2, and DHCR7 levels in RKO (shNC and sh*AURKA*) cells after SAPS3 knockdown. Data are presented as mean ± SD from at least three independent experiments. Statistical analyses were performed using unpaired Student’s *t* test (D) and two-way ANOVA test (B). ****p* < 0.001.

**Supplementary Figure 5. AURKA-mediated phosphorylation of SAPS3 regulates the AMPK-SREBP2-DHCR7 axis. A,** Co-IP analysis of SAPS3 indicating reduced phosphorylation levels in RKO-sh*AURKA* cells. **B,** Co-IP analysis of the phosphorylation levels of SAPS3 in RKO-sh*AURKA* cells reconstituted with WT-, T288D-, or D274N-AURKA. **C,** Western blot analysis of SAPS3, total AMPK, pAMPK, pSREBP2, nSREBP2, and DHCR7 in RKO-sh*AURKA* cells expressing WT-, T288D-, or D274N-AURKA. **D,** IC_50_ analysis of RSL3 in RKO-sh*AURKA* cells expressing WT-, T288D-, or D274N-AURKA. **E,** Representative FACS images and quantified values of lipid ROS levels in RKO-sh*AURKA* cells expressing WT-, T288D-, or D274N-AURKA. **F** and **G,** Co-IP analysis of phosphorylation levels for WT-, S523A-, S524A-, and S525A-SAPS3 mutants (F) or D-mut SAPS3 (G) in RKO cells. **H,** In vitro kinase assays were performed with commercial recombinant His-tagged AURKA protein as kinase and bacterially purified GST-SAPS3-21AA-WT or GST-SAPS3-21AA-DM as substrate. The reaction products were subject to WB analysis with indicated antibodies. **I,** Co-IP analysis of WT or D-mut SAPS3 phosphorylation in RKO cells treated with/without Alisertib (5 µM). **J,** Western blot analysis of total AMPK, pAMPK, pSREBP2, nSREBP2, and DHCR7 levels in RKO cells transfected with WT or D-mut SAPS3, with/without Alisertib treatment. **K**, **M** and **O,** Western blot analysis of WT- (**K**), S523A- (**M**), and S524A- (**O**) SAPS3 protein stability at indicated timepoints after CHX (100 µg/ml) treatment in SW480-sh*AURKA* cells, comparing AURKA-reconstituted versus control conditions. **L**, **N** and **P,** Quantified degradation kinetics (half-life) of WT- (**L**), S523A- (**N**), and S524A-SAPS3 (**P**) from panels K, M, and O, respectively. **Q,** Western blot analysis of WT- or D-mut SAPS3 stability in CHX-treated RKO cells, with/without MG132 (10 µM) under AURKA-expressing conditions. **R,** Western blot analysis of WT- or D-mut SAPS3 stability in CHX-treated RKO cells, with/without BafA1 (200 ng/ml) under AURKA-expressing conditions. Data are presented as mean ± SD from at least three independent experiments. Statistical analyses were performed using unpaired Student’s *t* test (E) and two-way ANOVA test (L). ****p* < 0.001.

**Supplementary Figure 6. Targeting AURKA potentiates chemotherapy efficacy in CRC.** **A,** Dose-response analysis of Oxaliplatin (Oxa) sensitivity in CRC (shNC and sh*AURKA*) cells treated for 48 h. **B,** Representative images (left) and quantification (right) of clonogenic assay with 500 RKO cells/well treated with 5-FU (60 μM). **C,** Viability of RKO (shNC and sh*AURKA*) cells with or without 5-FU (60 μM) for 48 h in combination with Necrostatin (Nec, 20 μM), Chloroquine (CQ, 30 μM), Disulfiram (Dis, 1 μM), Z-VAD-FMK (VAD, 25 μM) and Ferrostatin-1(Fer-1, 10 μM). **D,** Cell viability of shNC and sh*AURKA* SW480 cells treated with 5-FU (60 μM) for 48h in combination with VAD, Fer-1, 7-DHC, VAD+Fer-1, and VAD+7-DHC. **E,** Quantified GSH/GSSG ratios in RKO (shNC and sh*AURKA*) cells treated with 5-FU (60 μM, 24 h). **F,** Western blot analysis of C-Caspase3, Bax, and Bcl-2 protein expression in CRC (shNC and sh*AURKA*) cells with or without 5-FU treatment. **G,** Representative FACS plots showing Annexin V/PI staining of CRC (shNC and sh*AURKA*) cells with or without 5-FU (60 μM) treatment. **H,** Quantification of apoptotic cells (Annexin V^+^ and/or PI^+^) from three independent experiments. Data are presented as mean ± SD from at least three independent experiments. Statistical analyses were performed using unpaired Student’s *t* test [(B), (C), (F), and (G)]. **p* < 0.05, ***p* < 0.01, ****p* < 0.001.

**Supplementary Figure 7. AURKA inhibitor augments 5-FU efficacy in CRC cells. A,** Western blot analysis of pAURKA in RKO cells treated with Alisertib. **B,** RSL3-IC_50_ analysis in RKO cells after Alisertib treatment (1µM & 5µM). **C**-**E,** Representative FACS plots and quantified lipid ROS levels (**C**), MDA levels (**D**), and GSH/GSSG ratios (**E**) in RKO cells treated with RSL3 (2.5 µM) and Alisertib. **F,** Western blot analysis of AURKA, pAURKA, SAPS3, AMPK, pAMPK, pSREBP2, nSREBP2, and DHCR7 in Alisertib-treated RKO cells. Data are presented as mean ± SD from at least three independent experiments. Statistical analyses were performed using unpaired Student’s *t* test [ (C), (D), and (E)]. **p* < 0.05, ****p* < 0.001.
