# Supplementary material for: AURKA-mediated destabilization of SAPS3 drives ferroptosis evasion via 7-dehydrocholesterol biosynthesis in colorectal cancer
Source: Cell Death Dis. 2026 Mar 16;17(1):361. doi: 10.1038/s41419-026-08549-9 (PMC13039981; doi:10.1038/s41419-026-08549-9)
Supplement: Supplementary file 9 — Supplementary Tables [file 41419_2026_8549_MOESM9_ESM.docx]

**Supplementary Table 1. Clinical features of CRC patients in TMAs.**

| **Clinical feature** | **Group** | **Low expression**  **(n=123)** | **%** | **High expression**  **(n=123)** | **%** |
| --- | --- | --- | --- | --- | --- |
| Age | ≤55 | 34 | 27.6 | 40 | 32.5 |
|  | >55 | 89 | 72.4 | 83 | 67.5 |
| Gender | Men | 77 | 62.6 | 74 | 60.2 |
|  | Women | 46 | 37.4 | 49 | 39.8 |
| Localization | Colon | 64 | 52 | 88 | 71.5 |
|  | Rectum | 59 | 48 | 35 | 28.5 |
| Differentiation | Moderately | 37 | 30.1 | 50 | 40.7 |
|  | Poorly | 11 | 8.9 | 8 | 6.5 |
|  | Missing | 75 | 61 | 65 | 52.8 |

**Supplementary Table 2. Stage Ⅱ/Ⅲ CRC patients subjected to XELOX on AURKA expression.**

| **Clinical feature** | **Group** | **Low expression**  **(n=14)** | **%** | **High expression**  **(n=38)** | **%** |
| --- | --- | --- | --- | --- | --- |
| Age | ≤55 | 5 | 35.7 | 23 | 60.5 |
|  | >55 | 9 | 64.3 | 15 | 39.5 |
| Gender | Men | 7 | 50.0 | 23 | 60.5 |
|  | Women | 7 | 50.0 | 15 | 39.5 |
| Localization | Colon | 9 | 64.3 | 23 | 60.5 |
|  | Rectum | 5 | 35.7 | 14 | 36.8 |
|  | Cecum | 0 | 0 | 1 | 2.6 |
| Differentiation | Moderately | 9 | 64.3 | 25 | 65.8 |
|  | Poorly | 5 | 35.7 | 12 | 31.6 |
|  | Missing | 0 | 0 | 1 | 2.6 |

**Supplementary Table 3. Site-directed mutagenesis primers for AURKA/SAPS3.**

| **Primer name** | **Sequence** |
| --- | --- |
| hAURKA-BamHI Fw | CCTCAGGATCCACTAGTCCAGTGTGGTGGAAT |
| hAURKA-D274N Fw | CTGGAGAGCTTAAAATTGCAAATTTTGGGTGGTCAGTACATGC |
| hAURKA-D274N Rw | GCATGTACTGACCACCCAAAATTTGCAATTTTAAGCTCTCCAG |
| hAURKA-T288D Fw | CTCCATCTTCCAGGAGGACCGATCTCTGTGGCACCCTGGACTA |
| hAURKA-T288D Rw | TAGTCCAGGGTGCCACAGAGATCGGTCCTCCTGGAAGATGGAG |
| hAURKA-SacII Rw | CCTCACCGCGGGAAGGAAGGTCCGCTGGATT |
| hSAPS3-HindIII Fw | CCTCAAAGCTTATGTTTTGGAAATTTGATCTTCAC |
| hSAPS3-S523A Fw | AGTTACAACCTGCCATATTCATGCATCCAGTGATGATGAAATTG |
| hSAPS3-S523A Rw | CAATTTCATCATCACTGGATGCATGAATATGGCAGGTTGTAACT |
| hSAPS3-S524A Fw | TACAACCTGCCATATTCATTCAGCCAGTGATGATGAAATTGACT |
| hSAPS3-S524A Rw | AGTCAATTTCATCATCACTGGCTGAATGAATATGGCAGGTTGTA |
| hSAPS3-S525A Fw | ACCTGCCATATTCATTCATCCGCTGATGATGAAATTGACTTTAA |
| hSAPS3-S525A Rw | TTAAAGTCAATTTCATCATCAGCGGATGAATGAATATGGCAGGT |
| hSAPS3-S523A&S524A Fw | TACAACCTGCCATATTCATGCAGCCAGTGATGATGAAATTGACT |
| hSAPS3-S523A&S524A Rw | AGTCAATTTCATCATCACTGGCTGCATGAATATGGCAGGTTGTA |
| hSAPS3-XhoI Rw | CCTCACTCGAGTACAGGGCCATTCACTGAAG |

**Supplementary Table 4. The sequences of shRNA and siRNA.**

| **Gene name** | **Targeted sequences** | **Region** |
| --- | --- | --- |
| *AURKA* | CACATACCAAGAGACCTACAA | CDS |
| *AURKA* | CCTGTCTTACTGTCATTCGAA | CDS |
| *SCD* | CGTCCTTATGACAAGAACATT | CDS |
| *ETV4* | CCCTGTGTACATATAAATGAA | CDS |
| *DPEP1* | CGTCCTTATGACAAGAACATT | CDS |
| *DHCR7* | ACTTCAAGCTGTTCTTCAATG | CDS |
| *SREBP2* | CCTCAGATCATCAAGACAGAT | CDS |
| *SAPS3* | CGAGTATCAGACATCAACT | CDS |
| *SAPS3* | CAAGAAATTATAGAGCAGC | CDS |

**Supplementary Table 5. Primer sequences for qRT-PCR.**

| **Gene name** | **Primer** | **Sequence** |
| --- | --- | --- |
| *AURKA* | Fw | TGGTCGCCCTCTGGGTAAAG |
|  | Rw | TGCCGAAGGTGGGACTGTAT |
| *SCD* | Fw | GGAGCCACCGCTCTTACAAA |
|  | Rw | GAAAACTTGTGGTGGGCACG |
| *ETV4* | Fw | AGGTGGCTGGTGAGCGTTAC |
|  | Rw | CTCATCCAAGTGGGACAAAGG |
| *DPEP1* | Fw | GCTGGTGAAACAGACAGACA |
|  | Rw | CCTCCTTGATGTGATCCAGATG |
| *DHCR24* | Fw | CAAGTTCACCCACGAGTCCC |
|  | Rw | AGTAATTGCCAATGCTATTCAGC |
| *EBP* | Fw | CTGTCCCTGTGCTGGTTTGC |
|  | Rw | GATGTATCGGCTGTCTCCCT |
| *MSMO1* | Fw | TTGTGCAGTCATTGAAGATA |
|  | Rw | TAGAGTCTCCAAAGGATGTG |
| *SC5D* | Fw | GGTGGTTTATTTAAGTCTGTAC |
|  | Rw | ATGGTGGTCTGTATGATGAG |
| *CYP51A1* | Fw | TAGTTTCAGACGCAGGGACA |
|  | Rw | CTGCCAAGAGTAATCCAATAAG |
| *DHCR7* | Fw | AATCGCAACCCAACATTCCC |
|  | Rw | AGATGACGCTCGCCAGTGAA |
| *GAPDH* | Fw | GGACTCATGACCACAGTCCA |
|  | Rw | AGGCAGGGATGATGTTCTGG |

**Supplementary Table 6. Antibody list.**

| **Antibody name** | **Company** | **Host Species** | **Cat No.** | **Usage** | **RRID** |
| --- | --- | --- | --- | --- | --- |
| AURKA | Proteintech | Mouse | 66757-1-Ig | WB, IP, IHC, IF | AB_2882103 |
| β-Actin | Cell Signaling Technology | Rabbit | 4967 | WB | AB_330288 |
| Vinculin | ABclonal | Rabbit | A2752 | WB | AB_2863020 |
| SLC7A11 | Proteintech | Rabbit | 26864-1-AP | WB | AB_2880661 |
| GPX4 | Proteintech | Rabbit | 30388-1-AP | WB | AB_3086304 |
| ACSL4 | Proteintech | Rabbit | 22401-1-AP | WB | AB_2832995 |
| DHCR7 | Abcam | Rabbit | ab226784 | WB, IHC |  |
| SREBP2 | Abcam | Rabbit | ab30682 | IF | AB_779079 |
| SREBP2 | Proteintech | Rabbit | 28212-1-AP | WB | AB_2881091 |
| c-MYC | Proteintech | Mouse | 67447-1-Ig | WB | AB_2882681 |
| ZEB1 | Cell Signaling Technology | Rabbit | 3396 | WB | AB_1904164 |
| MAX | Proteintech | Rabbit | 10426-1-AP | WB | AB_2141660 |
| AMPKα | Proteintech | Rabbit | 10929-2-AP | WB | AB_2169568 |
| phospho-AMPKα | Abcam | Rabbit | ab133448 | WB | AB_2923300 |
| HA tag | Proteintech | Rabbit | 51064-2-AP | WB, IP | AB_11042321 |
| Flag tag | Sigma Aldrich | Mouse | F1804 | WB, IP |  |
| SAPS3 | Proteintech | Rabbit | 16944-1-AP | WB, IP, IF | AB_2183826 |
| Phospho-(Ser/Thr) | Abcam | Rabbit | ab17464 | WB | AB_443891 |
| Phospho-Aurora A (Thr288) | Cell Signaling Technology | Rabbit | 3079 | WB | AB_2061481 |
| His tag | Proteintech | Mouse | 66005-1-Ig | WB, IP | AB_11232599 |
| GAPDH | ABclonal | Mouse | AC054 | WB |  |
| Ki67 | Abcam | Rabbit | ab15580 | IHC | AB_443209 |
| 4-HNE | Abcam | Rabbit | ab48506 | IHC | AB_867452 |
| Bax | ABclonal | Rabbit | A12009 | WB, IHC | AB_2861644 |
| Bcl-2 | Abcam | Rabbit | ab196495 | WB, IHC | AB_2924862 |
| Cleaved Caspase 3 | Proteintech | Rabbit | 25128-1-AP | WB | AB_3073913 |
| Thiophosphate ester | Abcam | Rabbit | ab92570 | WB |  |
| HRP-mouse IgG | Cell Signaling Technology | Goat | 7076 | WB | AB_330924 |
| HRP-rabbit IgG | Cell Signaling Technology | Goat | 7074 | WB | AB_2099233 |
