# Supplementary material for: AURKA-mediated destabilization of SAPS3 drives ferroptosis evasion via 7-dehydrocholesterol biosynthesis in colorectal cancer
Source: Cell Death Dis. 2026 Mar 16;17(1):361. doi: 10.1038/s41419-026-08549-9 (PMC13039981; doi:10.1038/s41419-026-08549-9)
Supplement: Supplementary file 10 — Uncropped Western Blot Image [file 41419_2026_8549_MOESM10_ESM.pdf]

Fig.1E

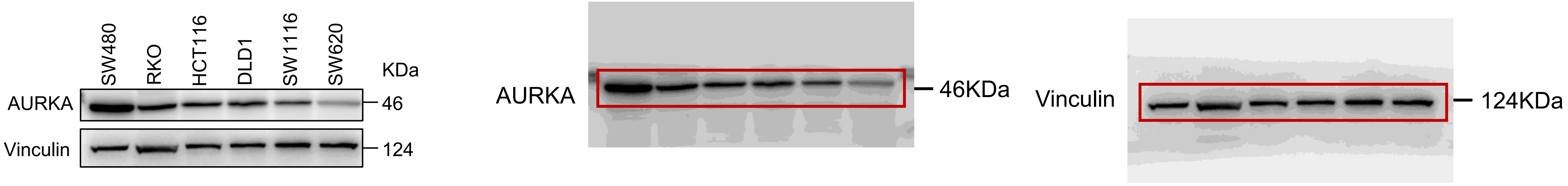
SW480
RKO
HCT116
DLD1
SW1116
SW620
KDa
AURKA
46
Vinculin
124
AURKA
46KDa
Vinculin
124KDa

Fig.1F

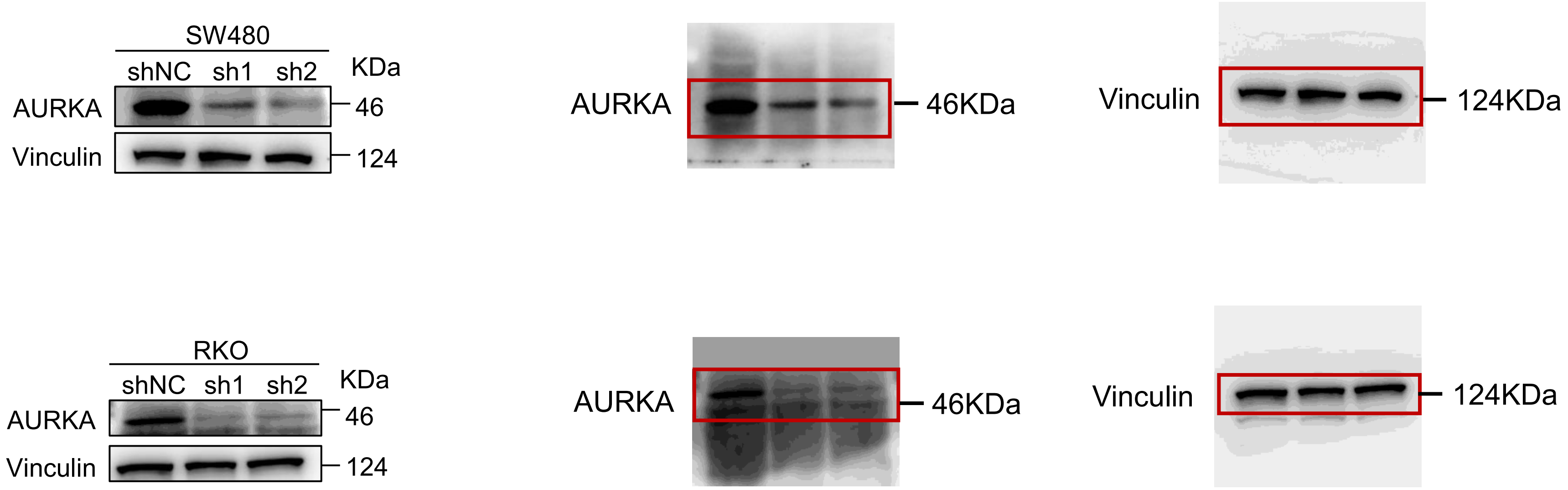
SW480
shNC
sh1
sh2
KDa
AURKA
46
Vinculin
124
AURKA
46KDa
Vinculin
124KDa
RKO
shNC
sh1
sh2
KDa
AURKA
46
Vinculin
124
AURKA
46KDa
Vinculin
124KDa

Fig.1M

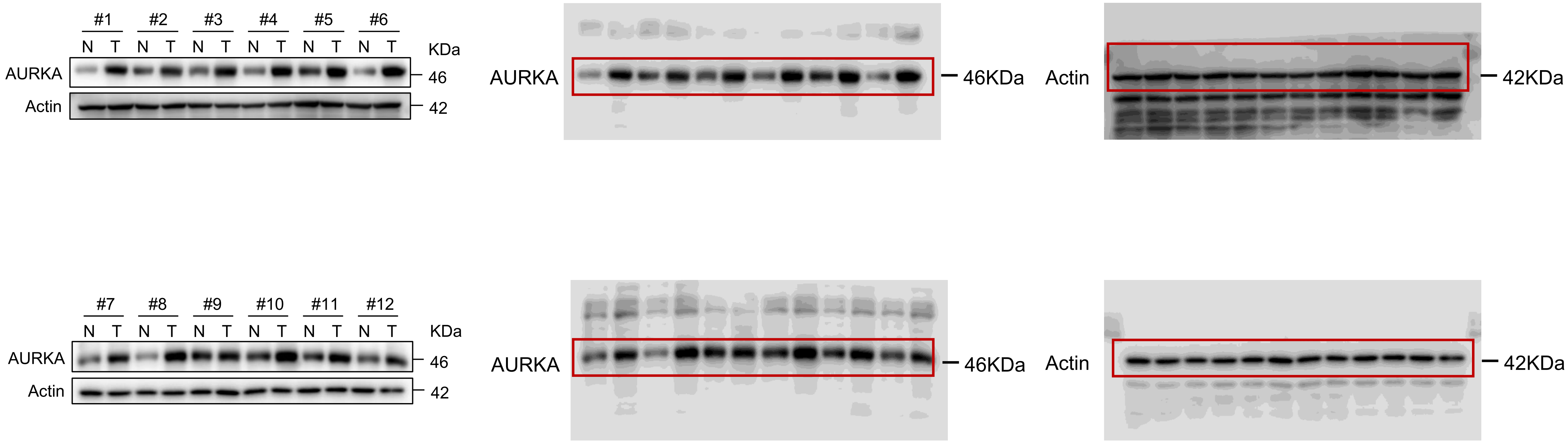
#1 #2 #3 #4 #5 #6
N T N T N T N T N T N T
KDa
AURKA
46
Actin
42
AURKA
46KDa
Actin
42KDa
#7 #8 #9 #10 #11 #12
N T N T N T N T N T N T
KDa
AURKA
46
Actin
42
AURKA
46KDa
Actin
42KDa

Fig.2A

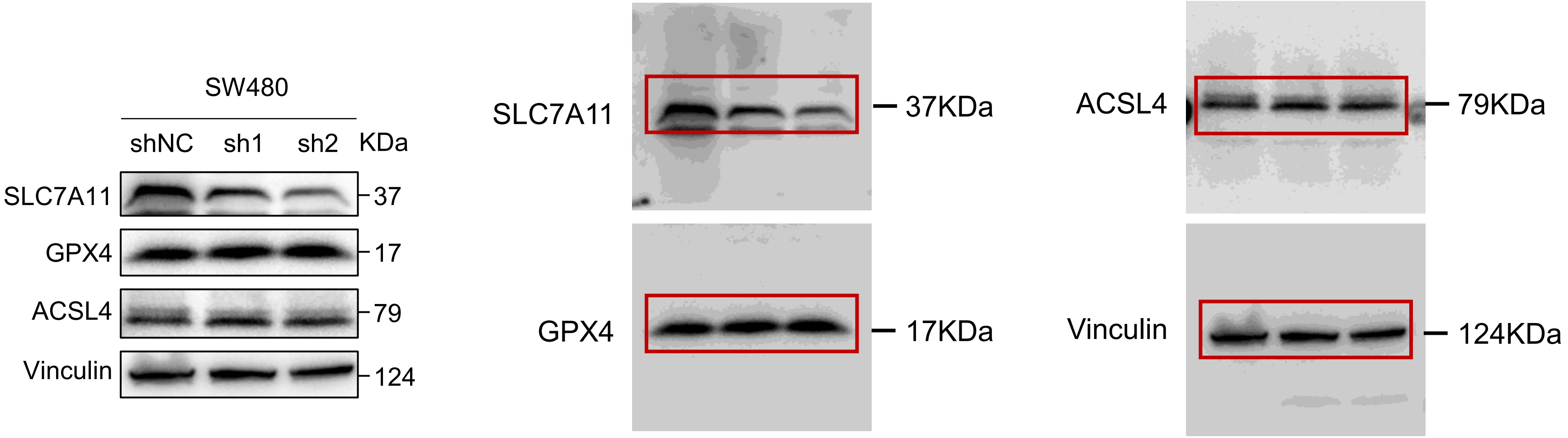
SW480
shNC sh1 sh2 KDa
SLC7A11 37
GPX4 17
ACSL4 79
Vinculin 124
SLC7A11 37KDa
GPX4 17KDa
ACSL4 79KDa
Vinculin 124KDa

Fig.2F

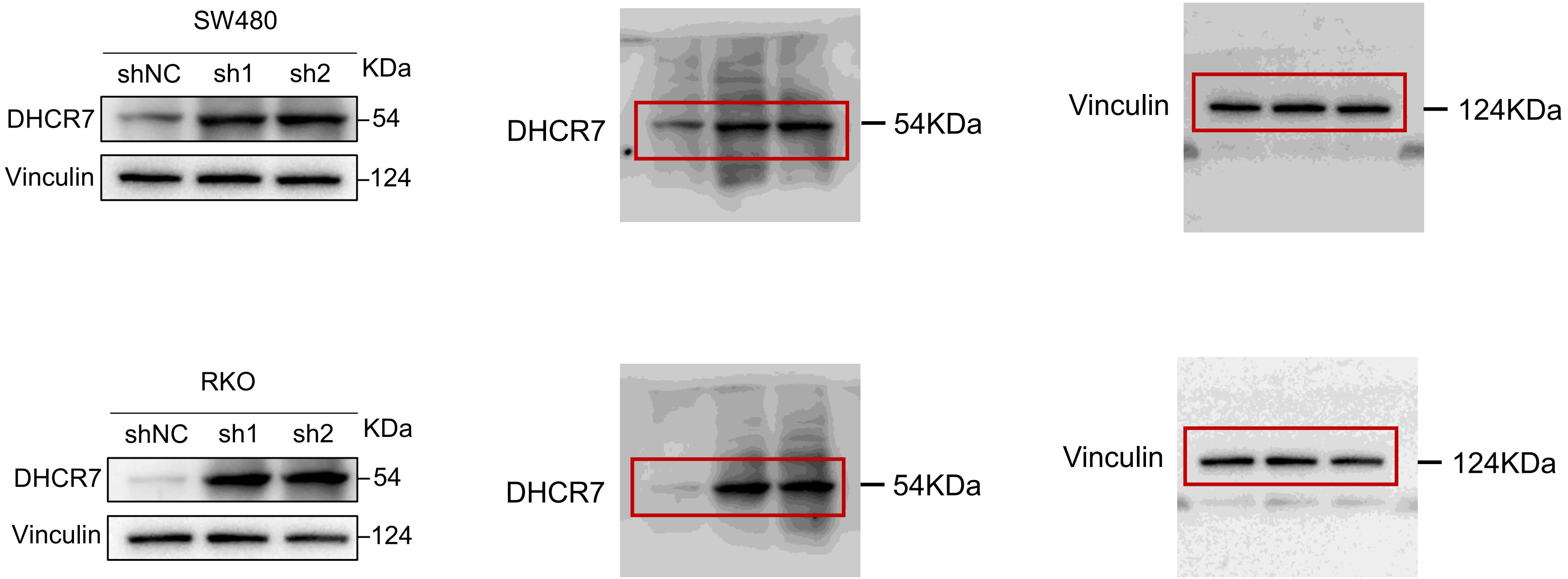
SW480
shNC sh1 sh2 KDa
DHCR7 54
Vinculin 124
DHCR7 54KDa
Vinculin 124KDa
RKO
shNC sh1 sh2 KDa
DHCR7 54
Vinculin 124
DHCR7 54KDa
Vinculin 124KDa

Fig.2G

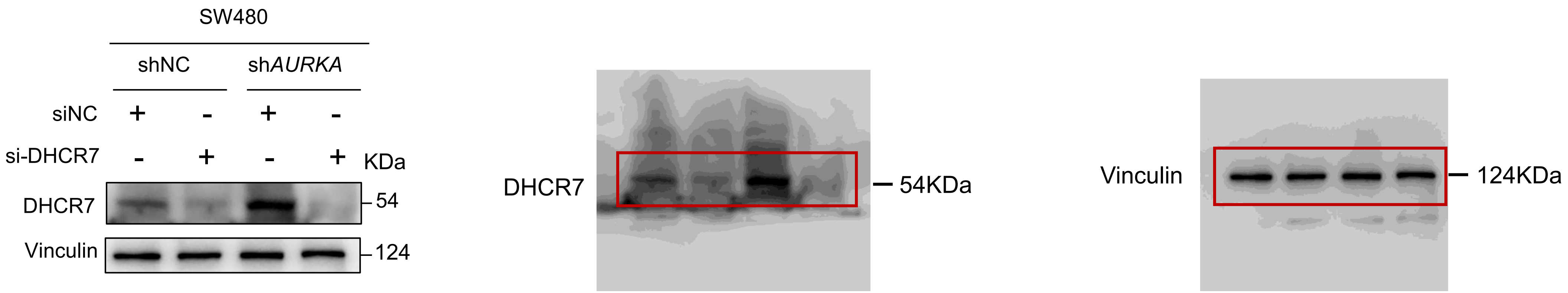

SW480
shNC
shAURKA
siNC + - + -
si-DHCR7 - + - +
KDa
DHCR7
54
Vinculin
124
DHCR7
54KDa
Vinculin
124KDa

Fig.3B

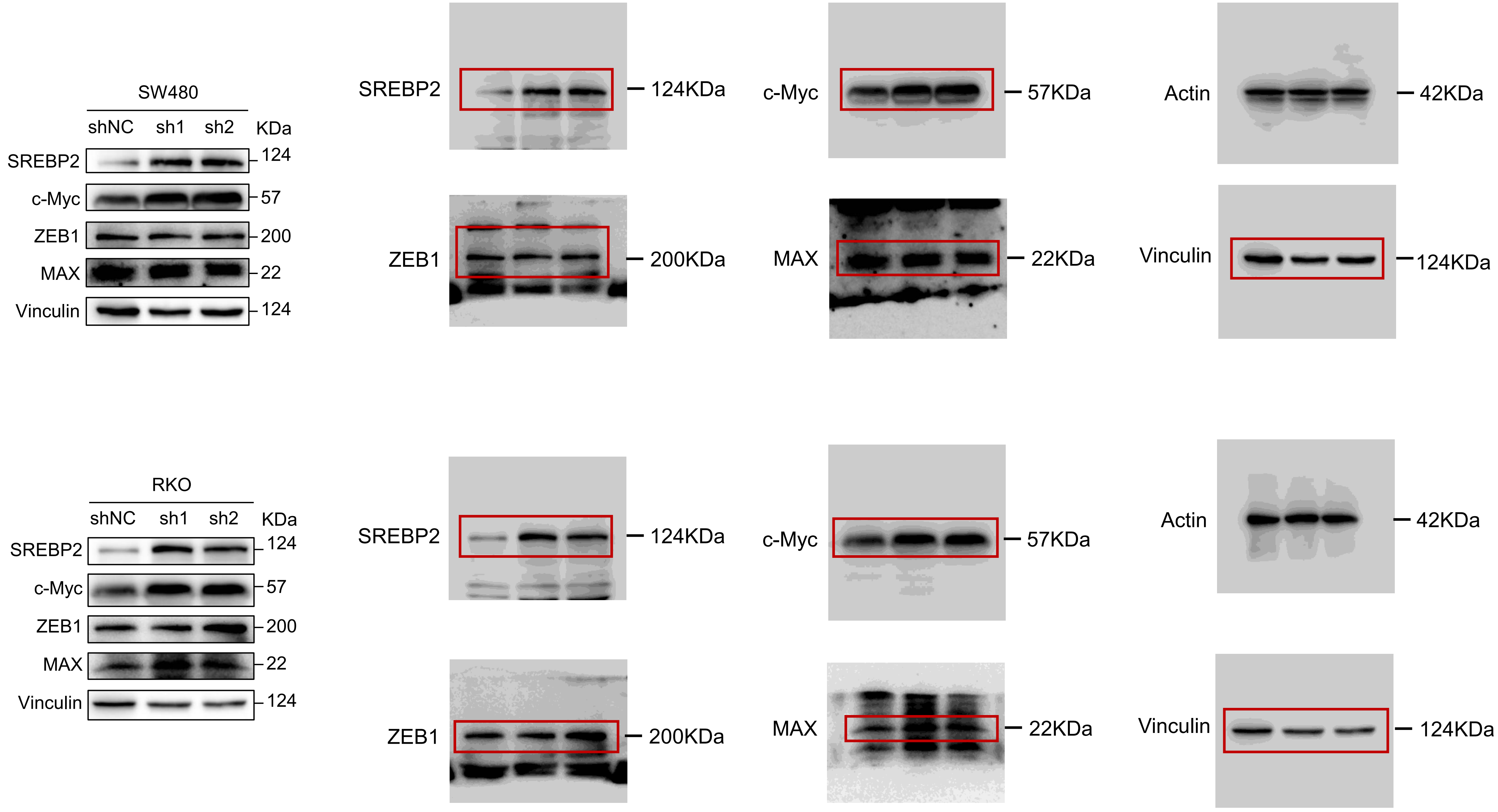
SW480
shNC sh1 sh2 KDa
SREBP2 124
c-Myc 57
ZEB1 200
MAX 22
Vinculin 124
SREBP2 124KDa
c-Myc 57KDa
Actin 42KDa
ZEB1 200KDa
MAX 22KDa
Vinculin 124KDa
RKO
shNC sh1 sh2 KDa
SREBP2 124
c-Myc 57
ZEB1 200
MAX 22
Vinculin 124
SREBP2 124KDa
c-Myc 57KDa
Actin 42KDa
ZEB1 200KDa
MAX 22KDa
Vinculin 124KDa

Fig.3C

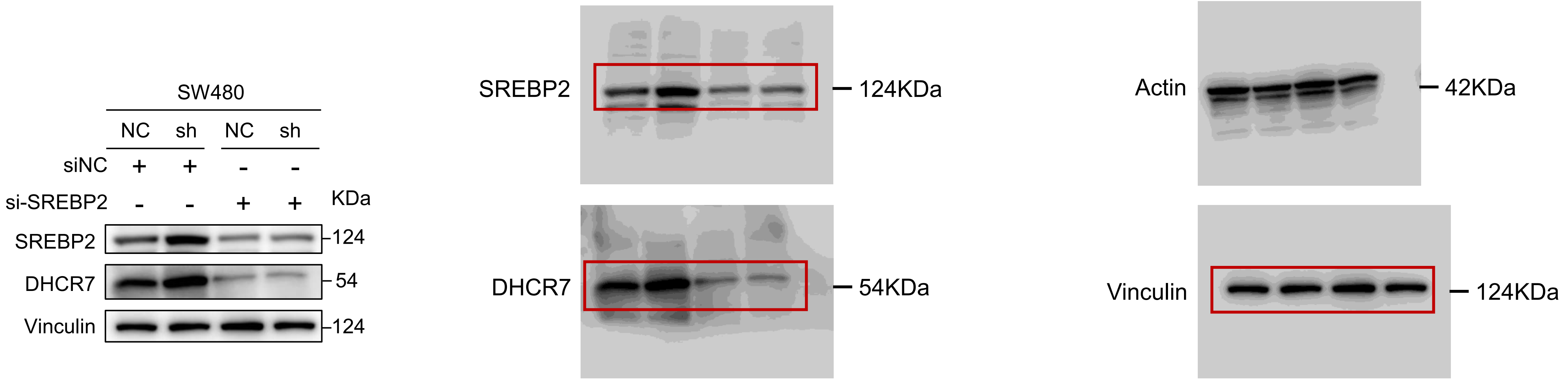
SW480
NC
sh
NC
sh
siNC
+
+
-
-
si-SREBP2
-
-
+
+
KDa
SREBP2
124
DHCR7
54
Vinculin
124
SREBP2
124KDa
DHCR7
54KDa
Actin
42KDa
Vinculin
124KDa

Fig.3H

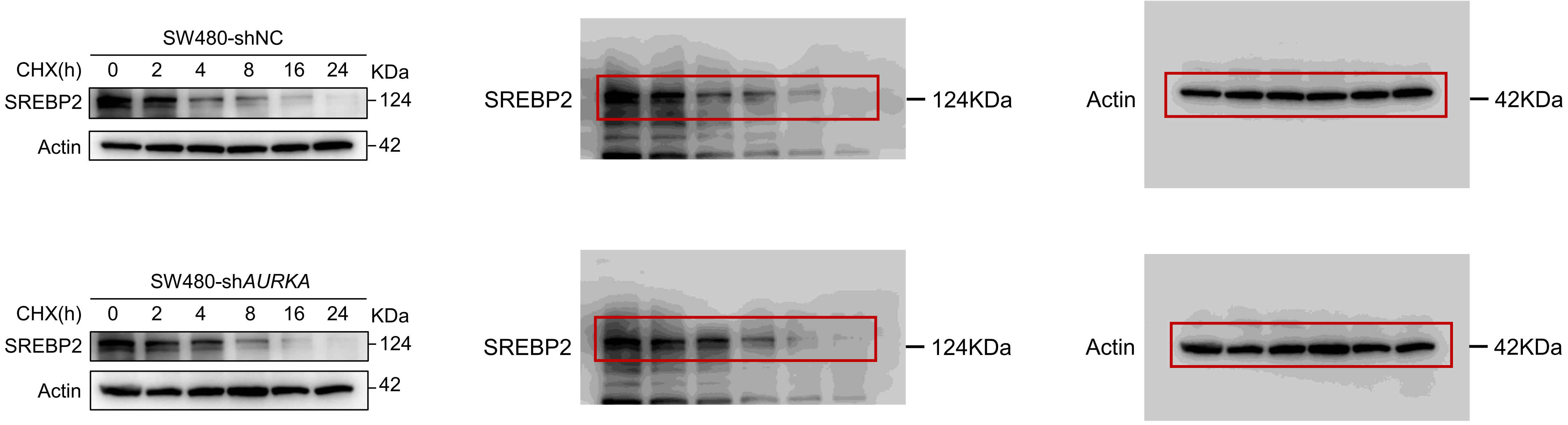
SW480-shNC
CHX(h)
0
2
4
8
16
24
KDa
SREBP2
124
Actin
42
SREBP2
124KDa
Actin
42KDa
SW480-sh*AURKA*
CHX(h)
0
2
4
8
16
24
KDa
SREBP2
124
Actin
42
SREBP2
124KDa
Actin
42KDa

Fig.3K

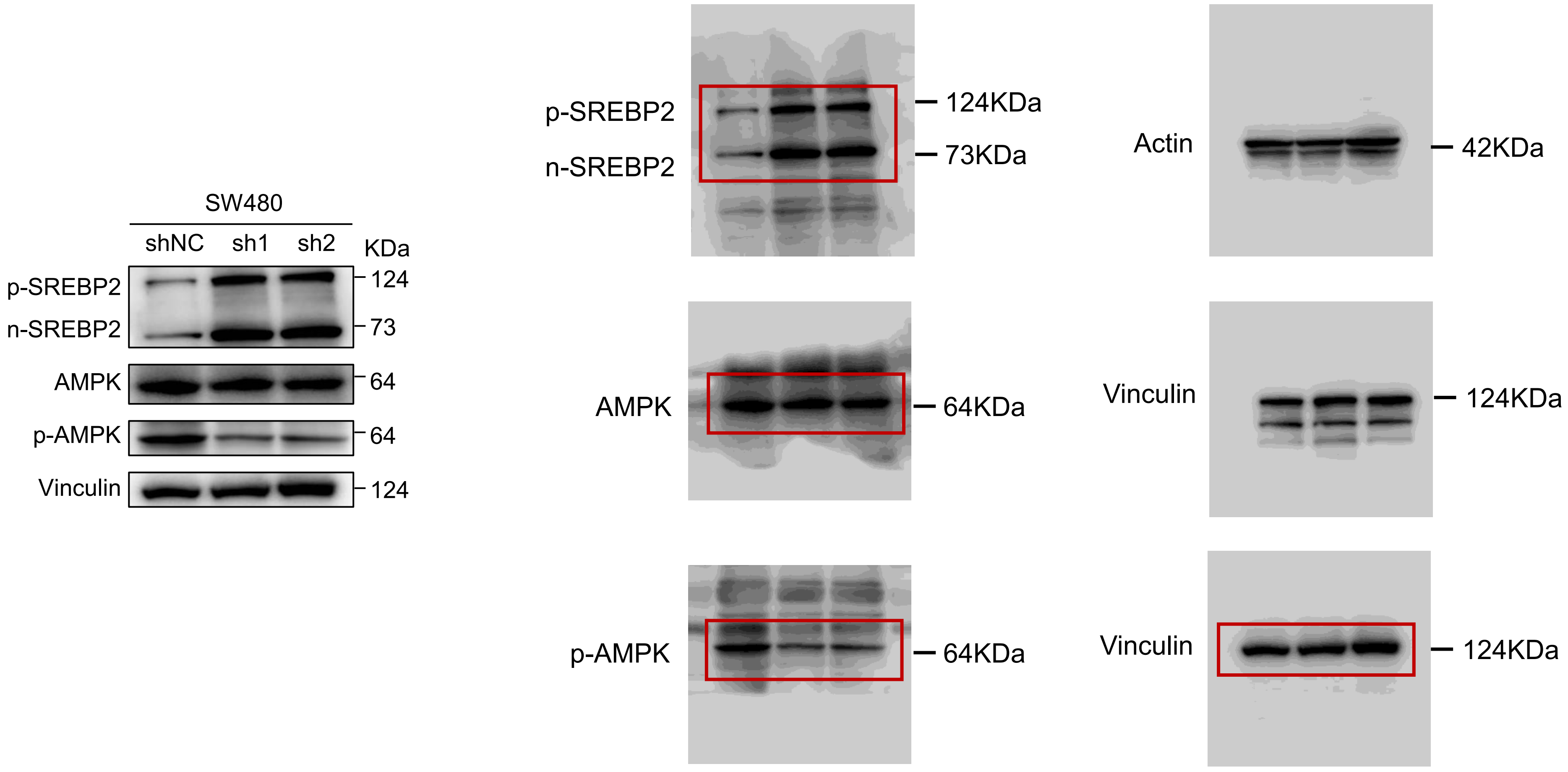
SW480
shNC
sh1
sh2
KDa
p-SREBP2
n-SREBP2
AMPK
p-AMPK
Vinculin
124
73
64
64
124
p-SREBP2
n-SREBP2
124KDa
73KDa
AMPK
64KDa
p-AMPK
64KDa
Actin
42KDa
Vinculin
124KDa
Vinculin
124KDa

Fig.3L

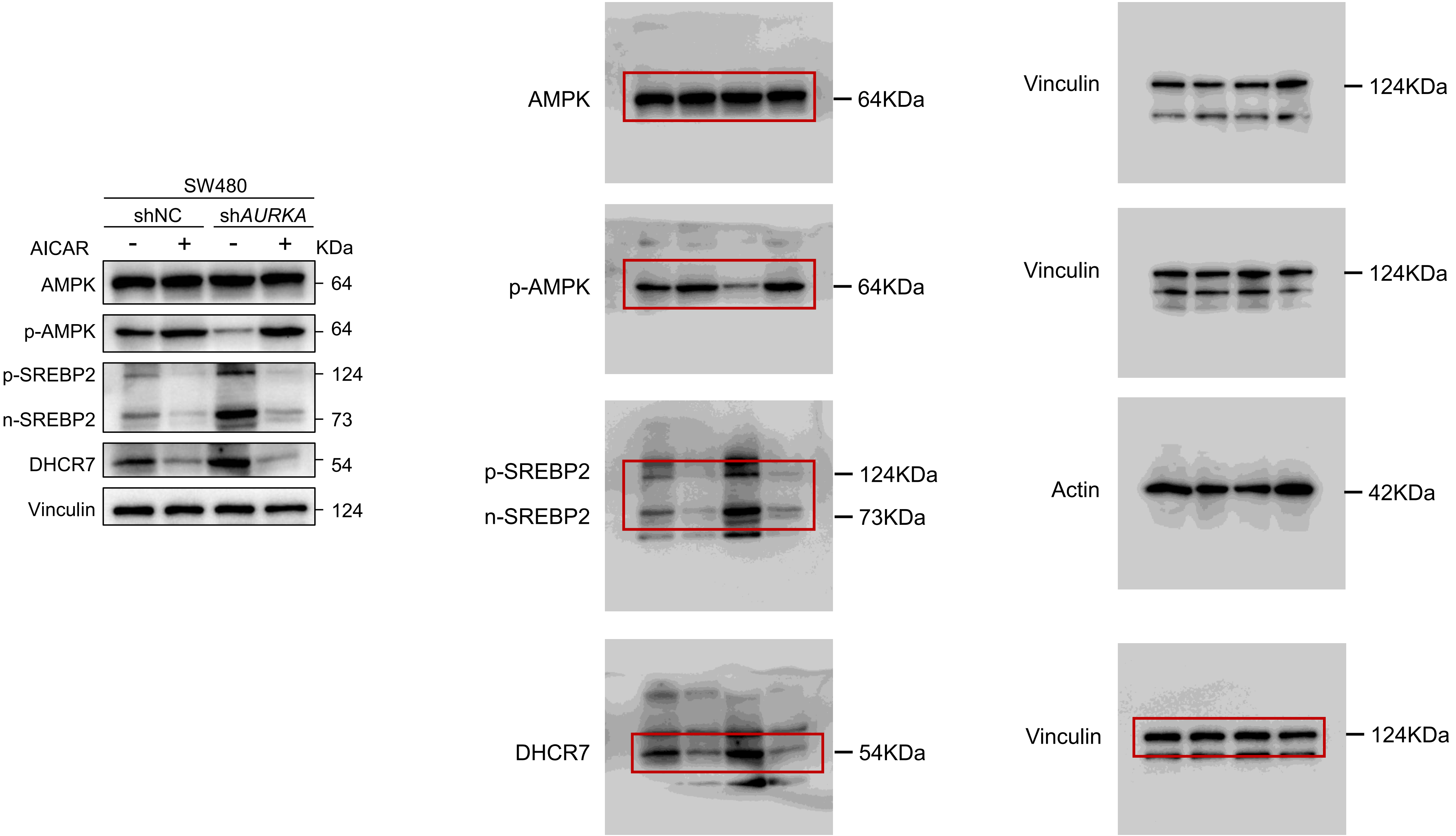
SW480
shNC
sh*AURKA*
AICAR
-
+
-
+
KDa
AMPK
64
p-AMPK
64
p-SREBP2
124
n-SREBP2
73
DHCR7
54
Vinculin
124
AMPK
64KDa
p-AMPK
64KDa
p-SREBP2
124KDa
n-SREBP2
73KDa
DHCR7
54KDa
Vinculin
124KDa
Vinculin
124KDa
Actin
42KDa
Vinculin
124KDa

Fig.4D

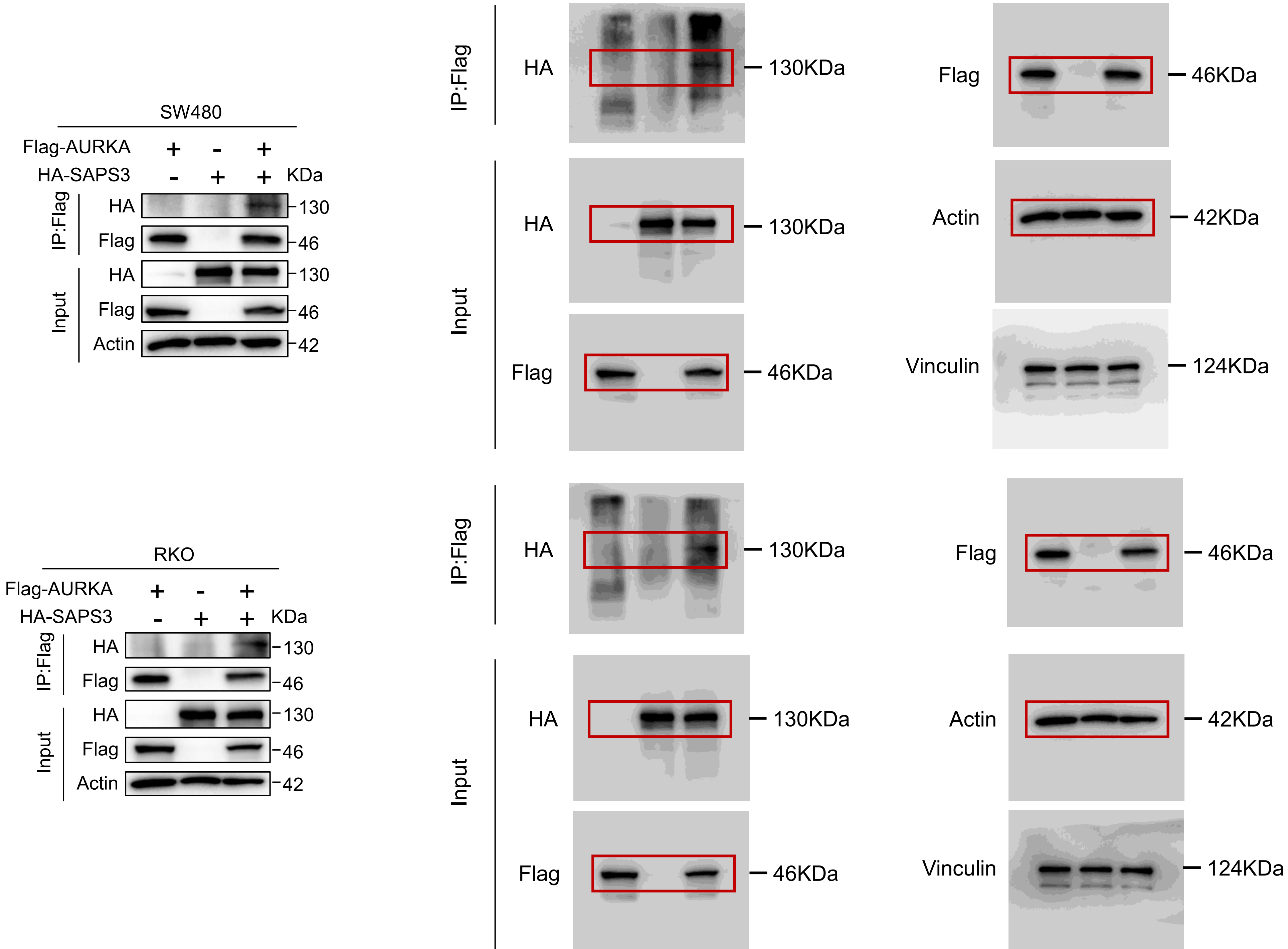
SW480
Flag-AURKA + - +
HA-SAPS3 - + + KDa
IP:Flag
HA 130
Flag 46
Input
HA 130
Flag 46
Actin 42
RKO
Flag-AURKA + - +
HA-SAPS3 - + + KDa
IP:Flag
HA 130
Flag 46
Input
HA 130
Flag 46
Actin 42
IP:Flag
HA 130KDa
Flag 46KDa
Input
HA 130KDa
Actin 42KDa
Flag 46KDa
Vinculin 124KDa
IP:Flag
HA 130KDa
Flag 46KDa
Input
HA 130KDa
Actin 42KDa
Flag 46KDa
Vinculin 124KDa

Fig.4E

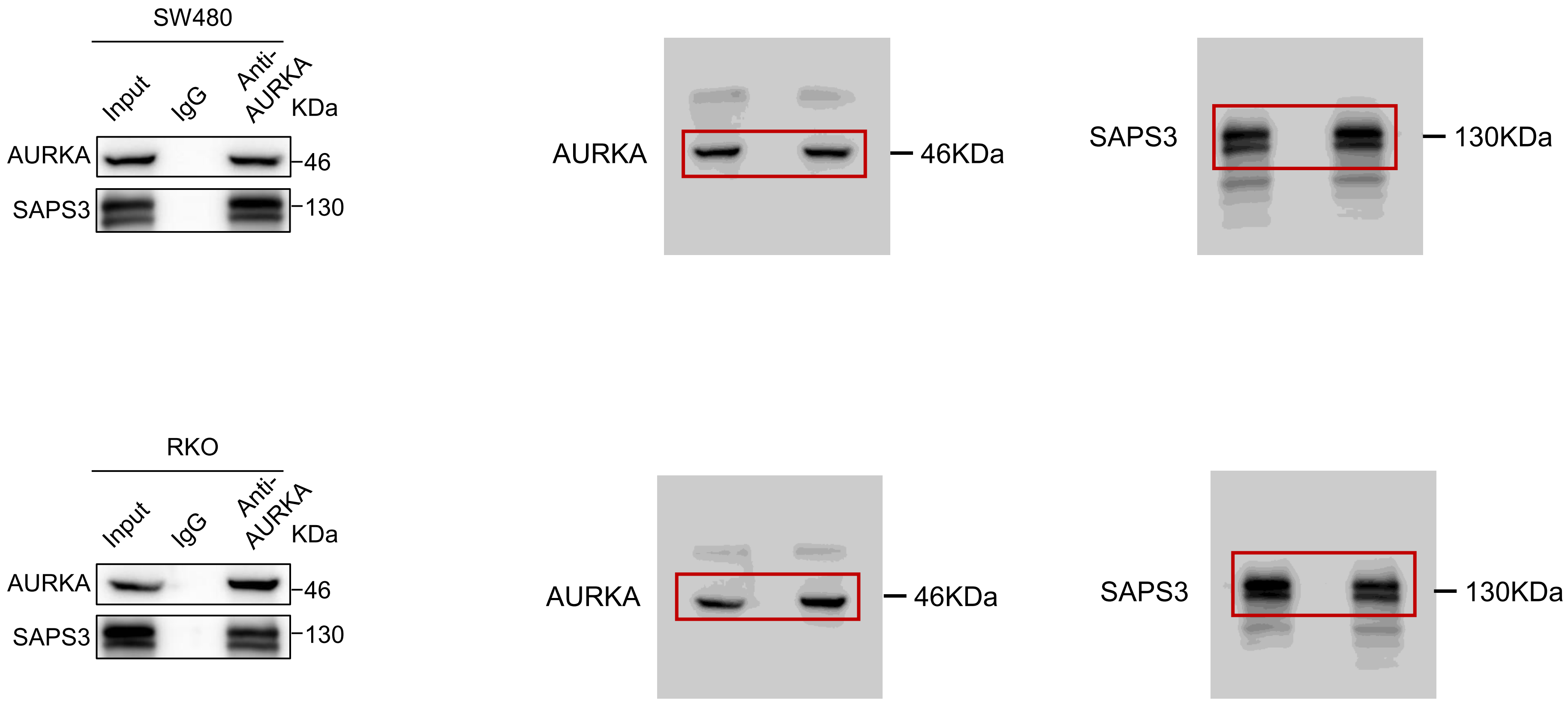
SW480
Input
IgG
Anti-AURKA
KDa
AURKA
SAPS3
46
130
AURKA
46KDa
SAPS3
130KDa
RKO
Input
IgG
Anti-AURKA
KDa
AURKA
SAPS3
46
130
AURKA
46KDa
SAPS3
130KDa

Fig.4G

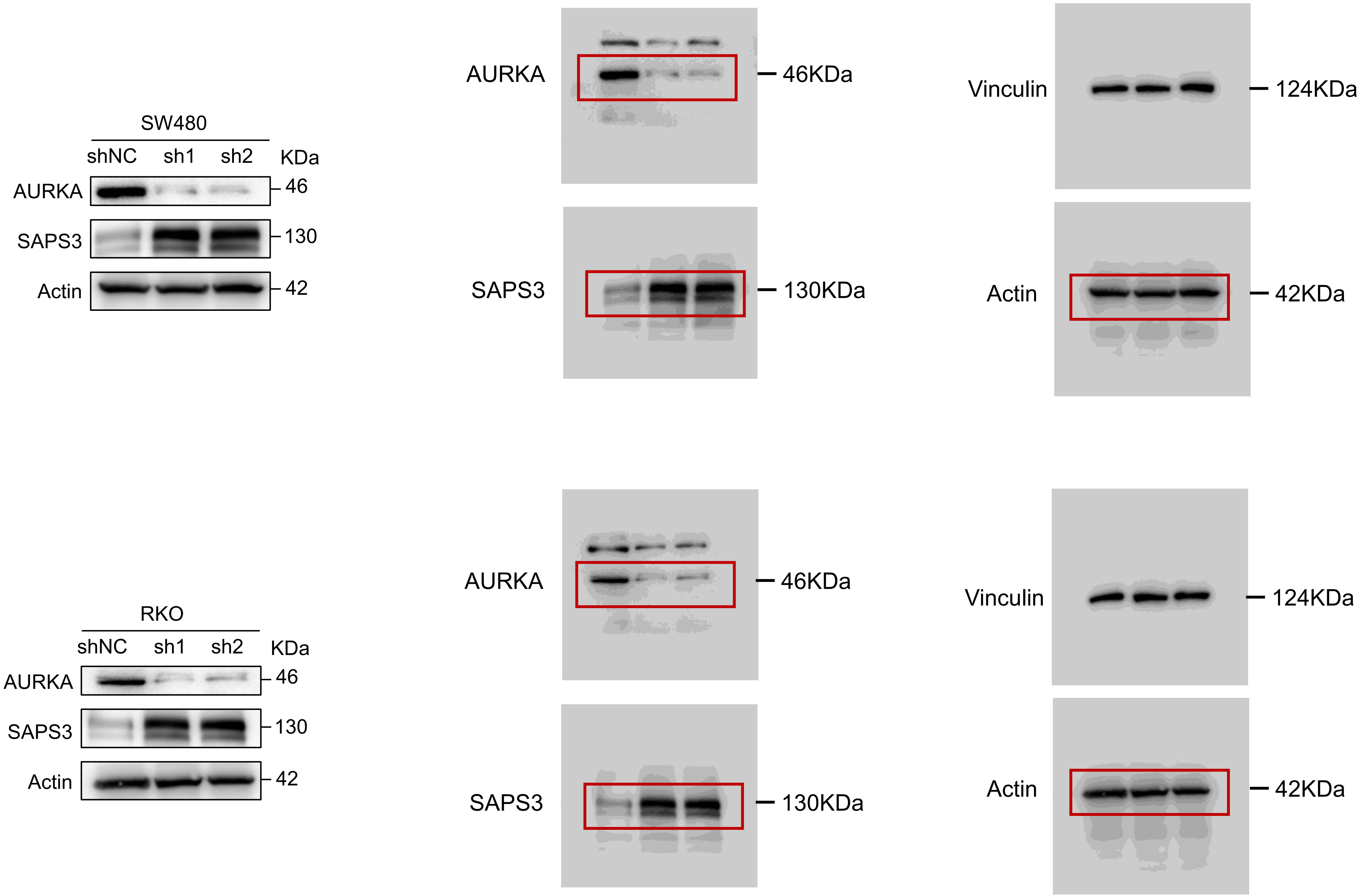
SW480
shNC
sh1
sh2
KDa
AURKA
46
SAPS3
130
Actin
42
RKO
shNC
sh1
sh2
KDa
AURKA
46
SAPS3
130
Actin
42
AURKA
46KDa
SAPS3
130KDa
Vinculin
124KDa
Actin
42KDa
AURKA
46KDa
SAPS3
130KDa
Vinculin
124KDa
Actin
42KDa

Fig.4H

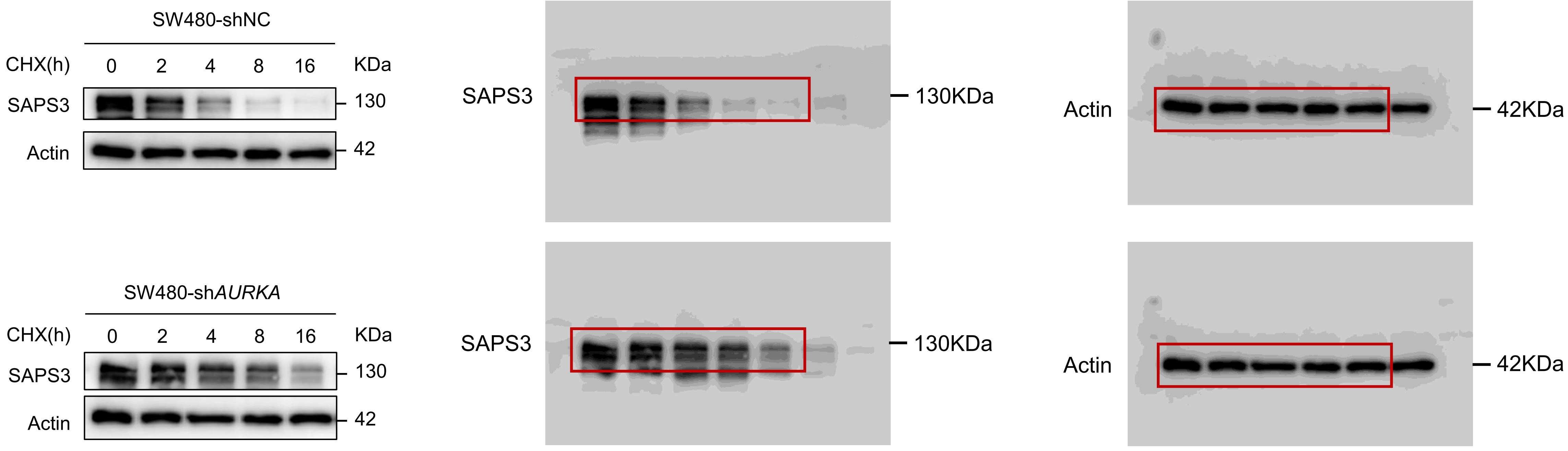
SW480-shNC
CHX(h) 0 2 4 8 16 KDa
SAPS3
130
Actin
42
SW480-shAURKA
CHX(h) 0 2 4 8 16 KDa
SAPS3
130
Actin
42
SAPS3
130KDa
Actin
42KDa
SAPS3
130KDa
Actin
42KDa

Fig.4L

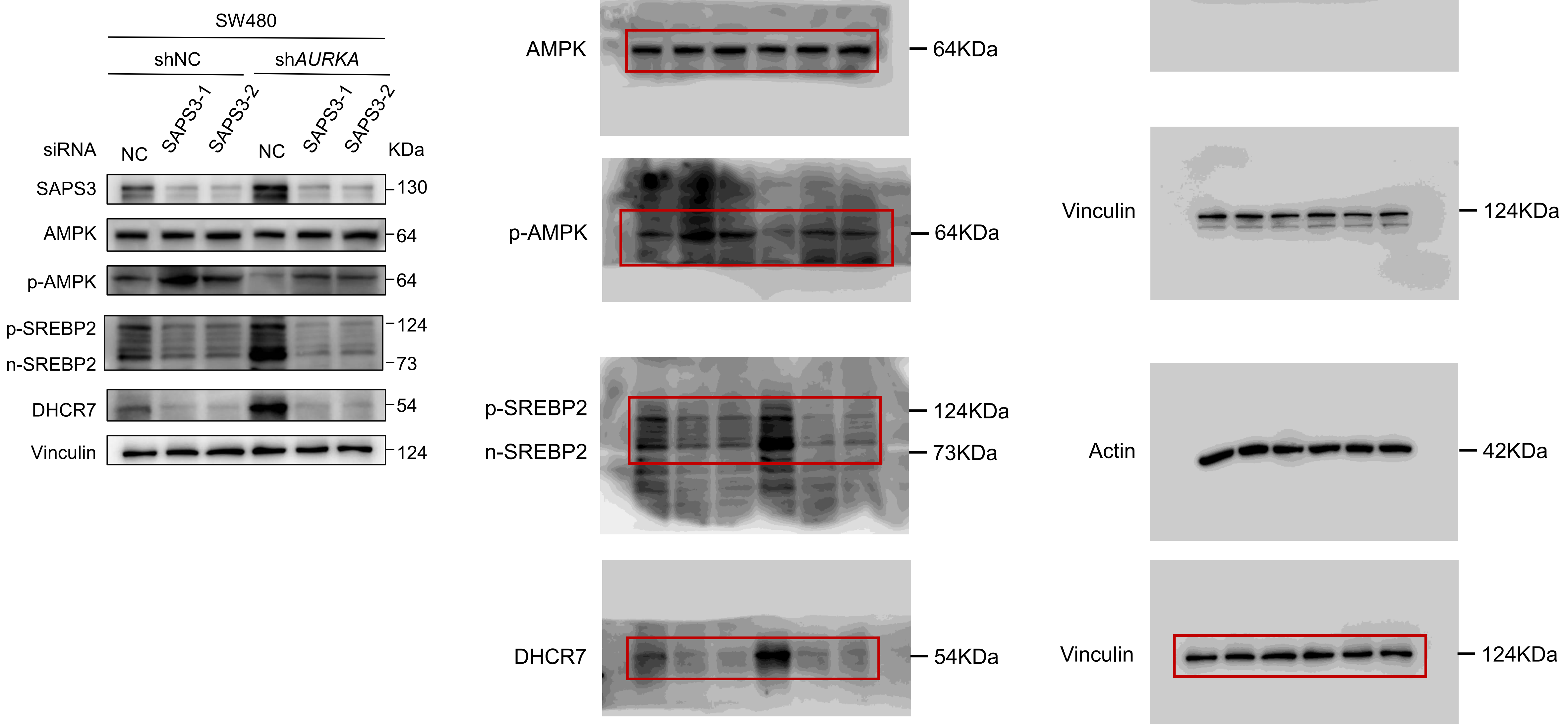
SW480
shNC
shAURKA
siRNA
NC
SAPS3-1
SAPS3-2
NC
SAPS3-1
SAPS3-2
KDa
SAPS3
130
AMPK
64
p-AMPK
64
p-SREBP2
124
n-SREBP2
73
DHCR7
54
Vinculin
124
SAPS3
130KDa
AMPK
64KDa
p-AMPK
64KDa
p-SREBP2
124KDa
n-SREBP2
73KDa
DHCR7
54KDa
Actin
42KDa
Vinculin
124KDa
Actin
42KDa
Vinculin
124KDa

Fig.5A

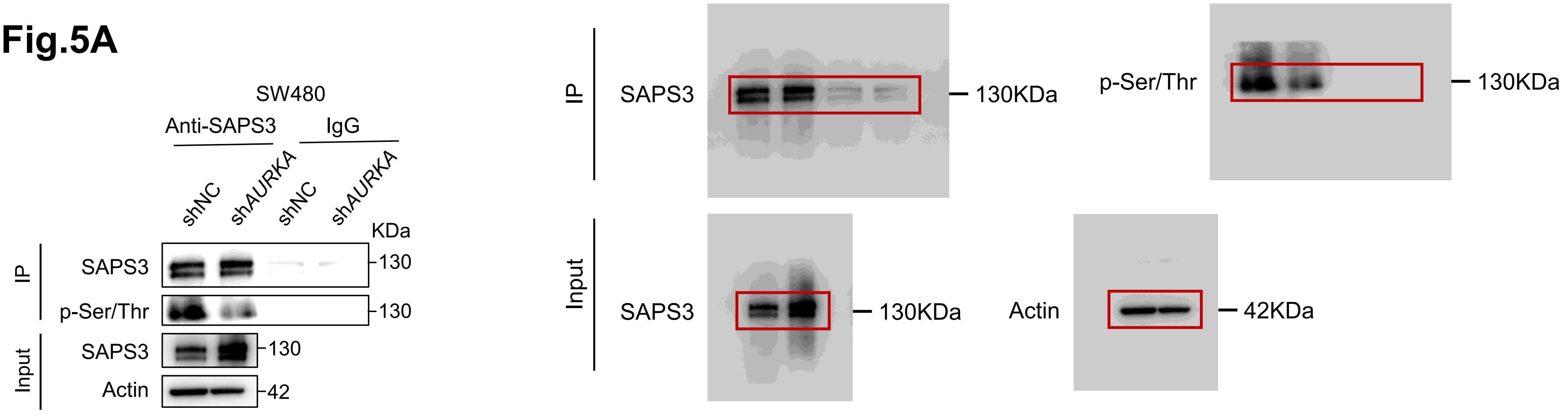

SW480
Anti-SAPS3
IgG
shNC
shAURKA
shNC
shAURKA
KDa
IP
SAPS3
130
p-Ser/Thr
130
Input
SAPS3
130
Actin
42
IP
SAPS3
130KDa
p-Ser/Thr
130KDa
Input
SAPS3
130KDa
Actin
42KDa

Fig.5B

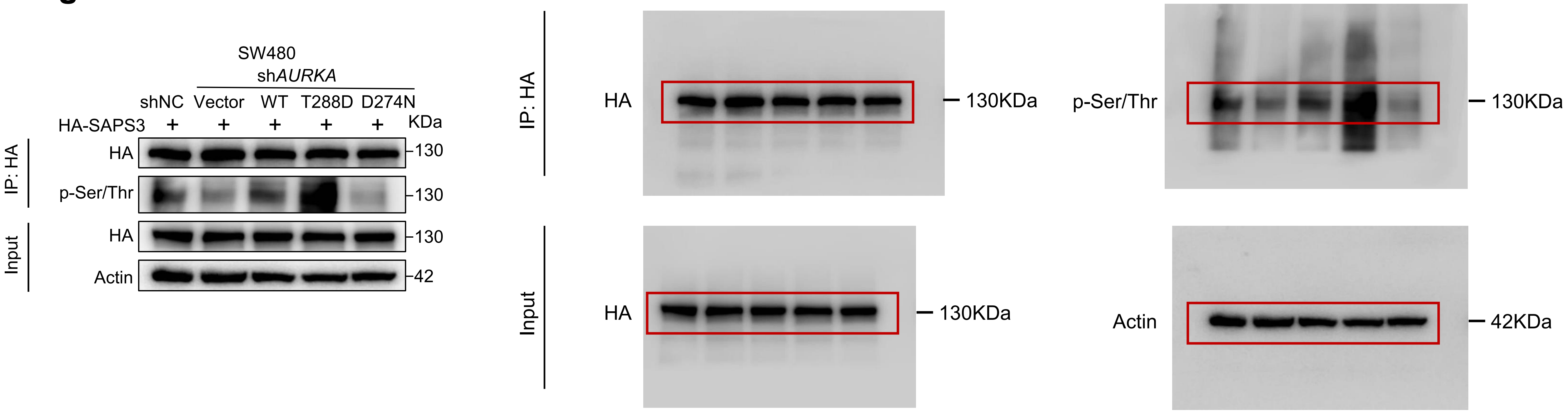

SW480
shAURKA
shNC
Vector
WT
T288D
D274N
HA-SAPS3
+
+
+
+
+
KDa
IP: HA
HA
130
p-Ser/Thr
130
Input
HA
130
Actin
42
IP: HA
HA
130KDa
p-Ser/Thr
130KDa
Input
HA
130KDa
Actin
42KDa

Fig.5C

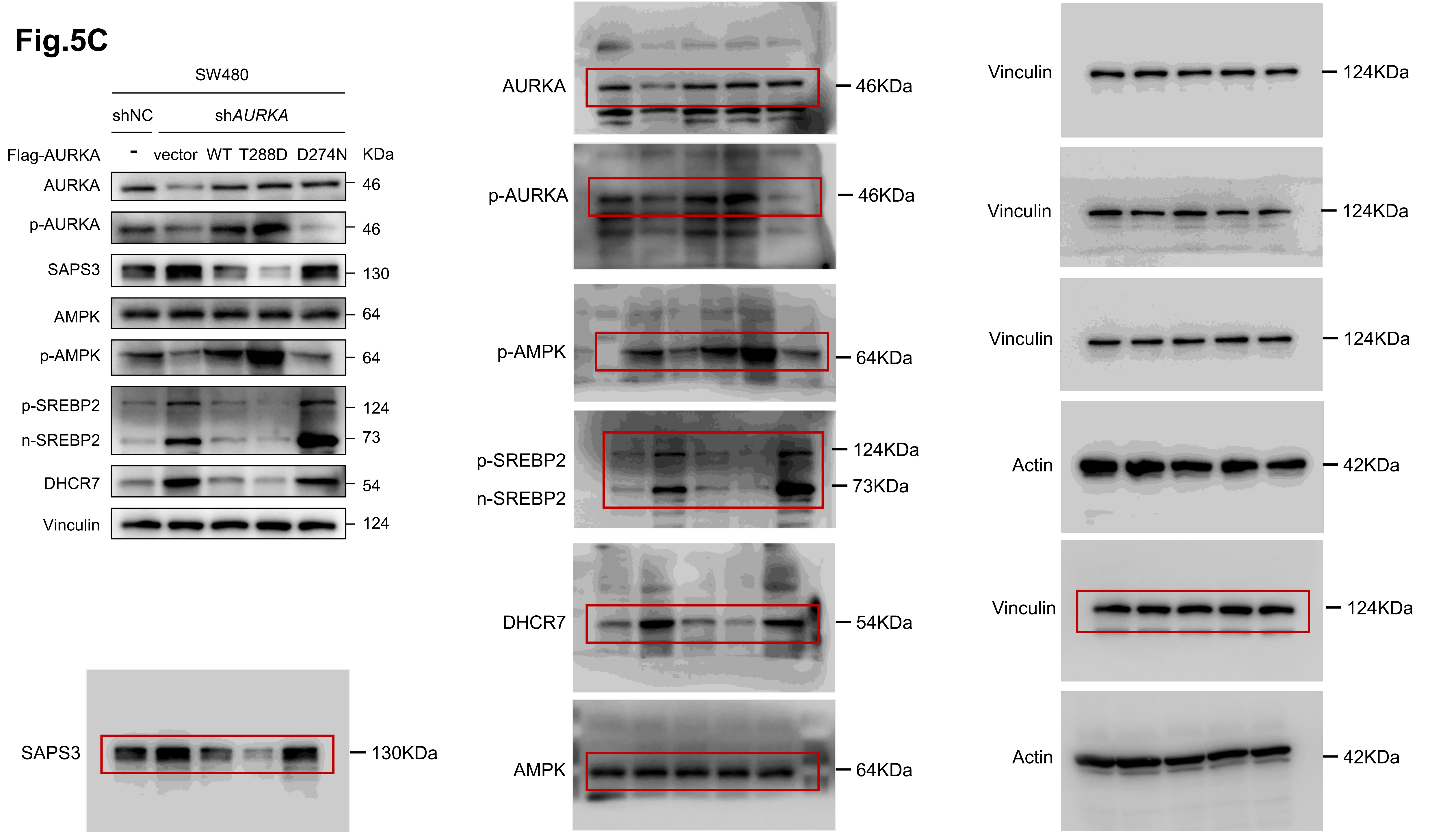
SW480
shNC
shAURKA
Flag-AURKA
-
vector
WT
T288D
D274N
KDa
AURKA
46
p-AURKA
46
SAPS3
130
AMPK
64
p-AMPK
64
p-SREBP2
124
n-SREBP2
73
DHCR7
54
Vinculin
124
SAPS3
130KDa
AURKA
46KDa
p-AURKA
46KDa
p-AMPK
64KDa
p-SREBP2
n-SREBP2
124KDa
73KDa
DHCR7
54KDa
AMPK
64KDa
Vinculin
124KDa
Vinculin
124KDa
Vinculin
124KDa
Actin
42KDa
Vinculin
124KDa
Actin
42KDa

Fig.5H

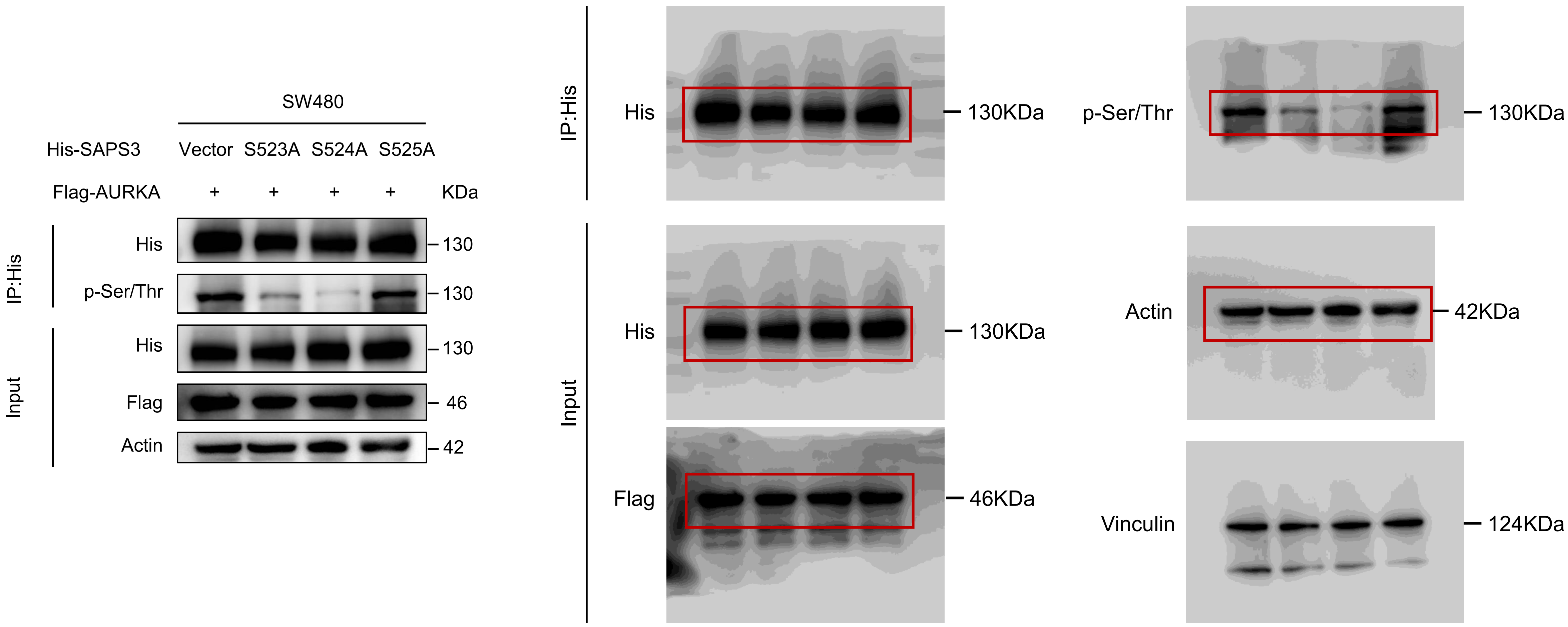
SW480
His-SAPS3
Vector
S523A
S524A
S525A
Flag-AURKA
+
+
+
+
KDa
IP:His
His
130
p-Ser/Thr
130
Input
His
130
Flag
46
Actin
42
IP:His
His
130KDa
p-Ser/Thr
130KDa
Input
His
130KDa
Actin
42KDa
Flag
46KDa
Vinculin
124KDa

Fig.5I

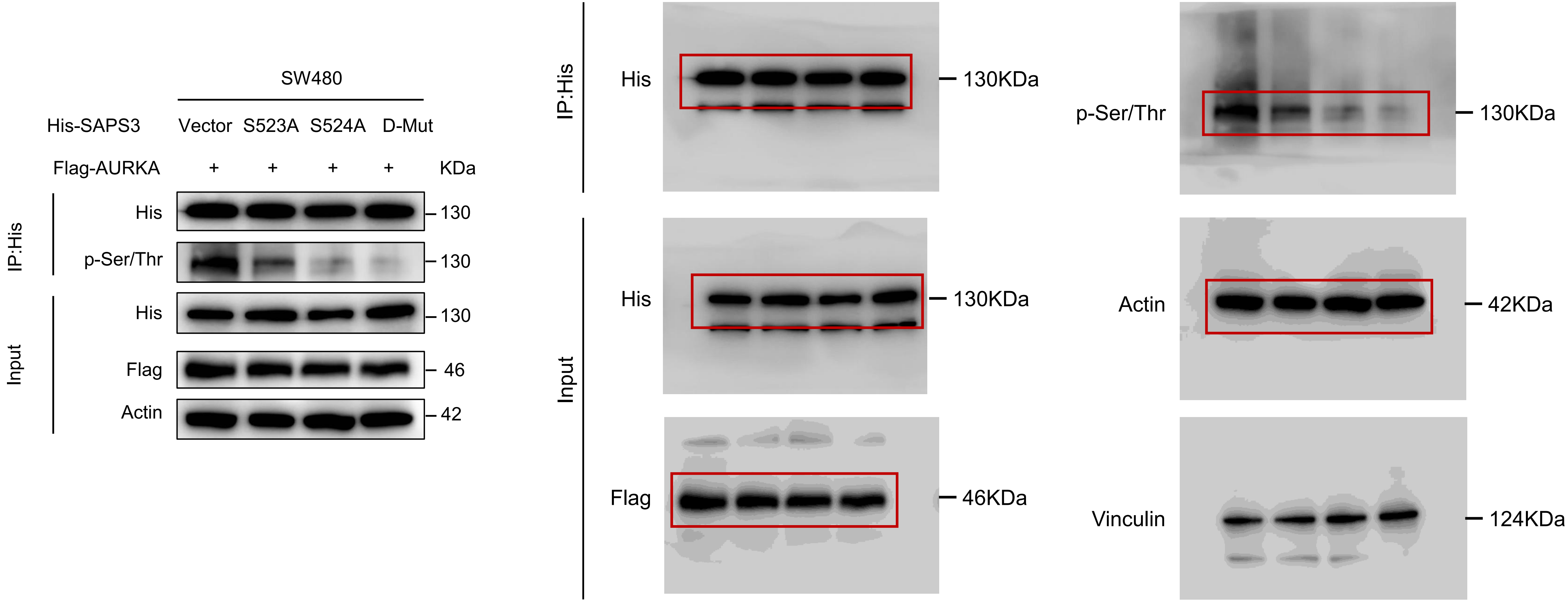

SW480
His-SAPS3
Vector
S523A
S524A
D-Mut
Flag-AURKA
+
+
+
+
KDa
IP:His
His
p-Ser/Thr
130
130
Input
His
Flag
Actin
130
46
42
IP:His
His
130KDa
p-Ser/Thr
130KDa
Input
His
130KDa
Actin
42KDa
Flag
46KDa
Vinculin
124KDa

Fig.5J

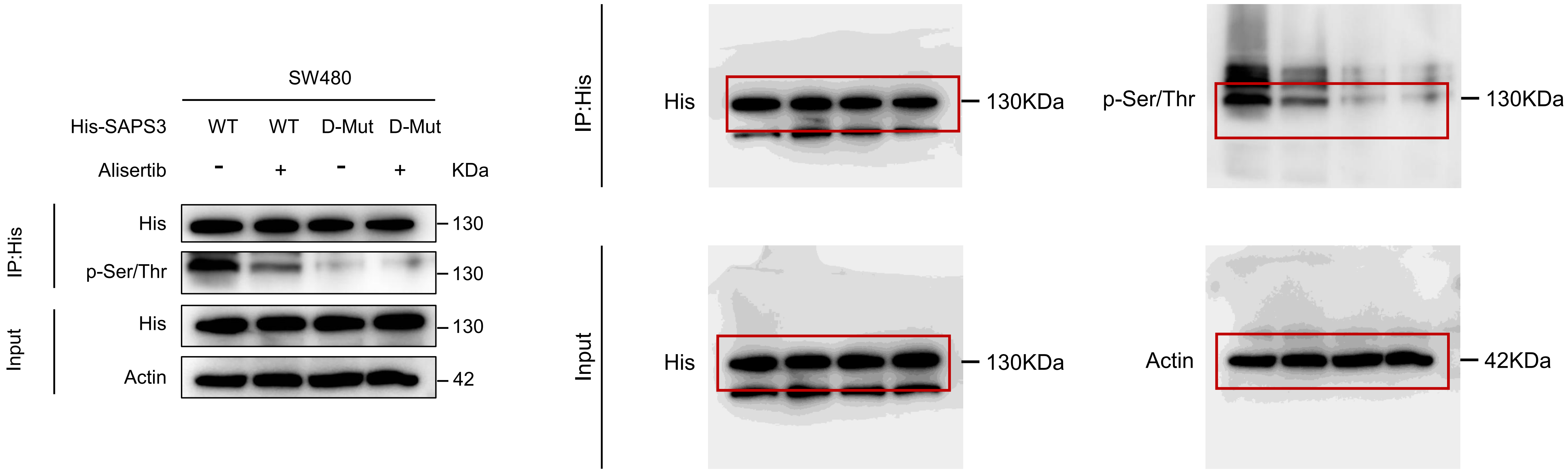
SW480
His-SAPS3
WT
WT
D-Mut
D-Mut
Alisertib
-
+
-
+
KDa
IP:His
His
130
p-Ser/Thr
130
Input
His
130
Actin
42
IP:His
His
130KDa
p-Ser/Thr
130KDa
Input
His
130KDa
Actin
42KDa

Fig.5K

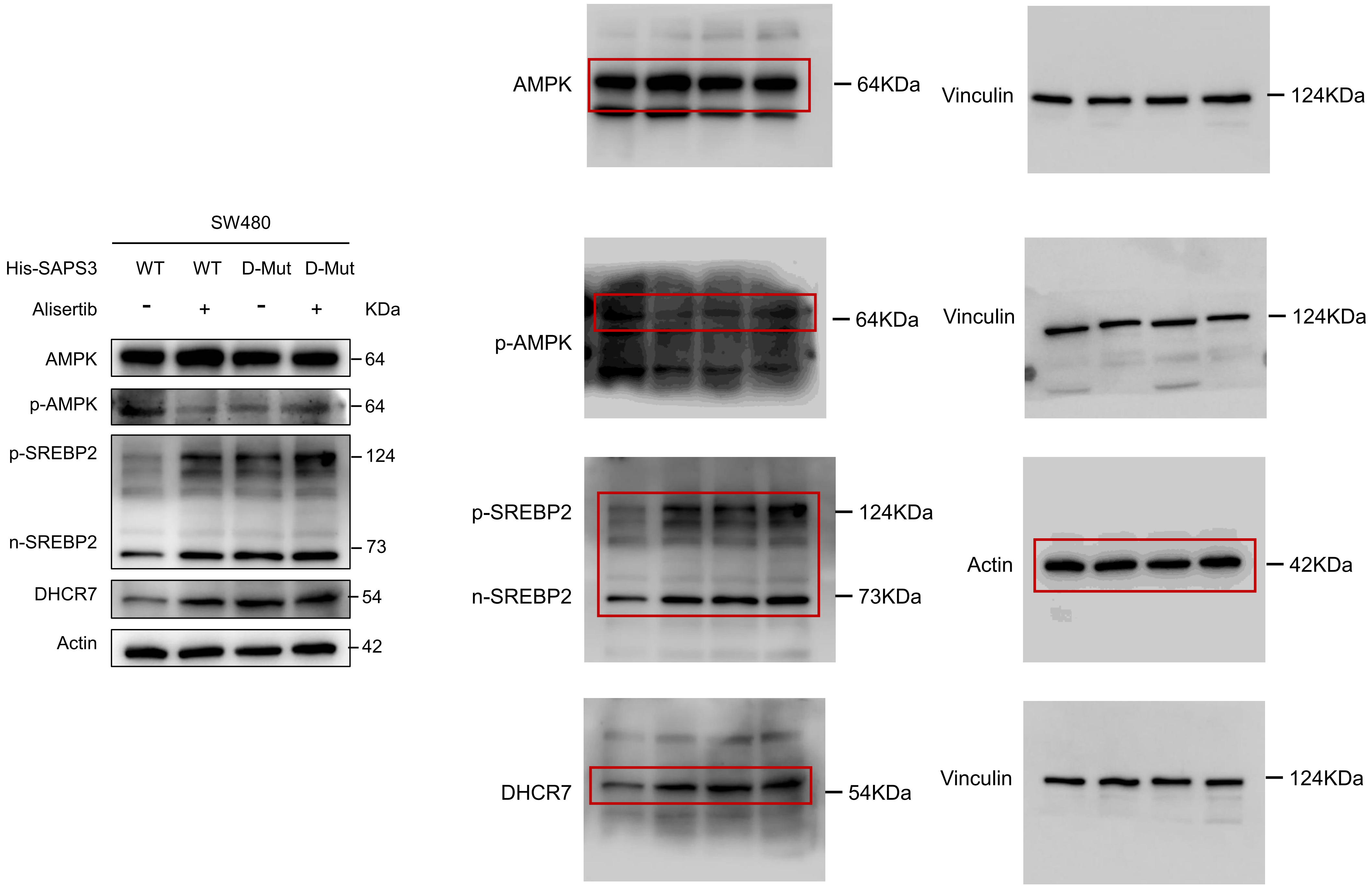
SW480
His-SAPS3
WT
WT
D-Mut
D-Mut
Alisertib
-
+
-
+
KDa
AMPK
64
p-AMPK
64
p-SREBP2
124
n-SREBP2
73
DHCR7
54
Actin
42
AMPK
64KDa
Vinculin
124KDa
p-AMPK
64KDa
Vinculin
124KDa
p-SREBP2
124KDa
n-SREBP2
73KDa
Actin
42KDa
DHCR7
54KDa
Vinculin
124KDa

Fig.5L

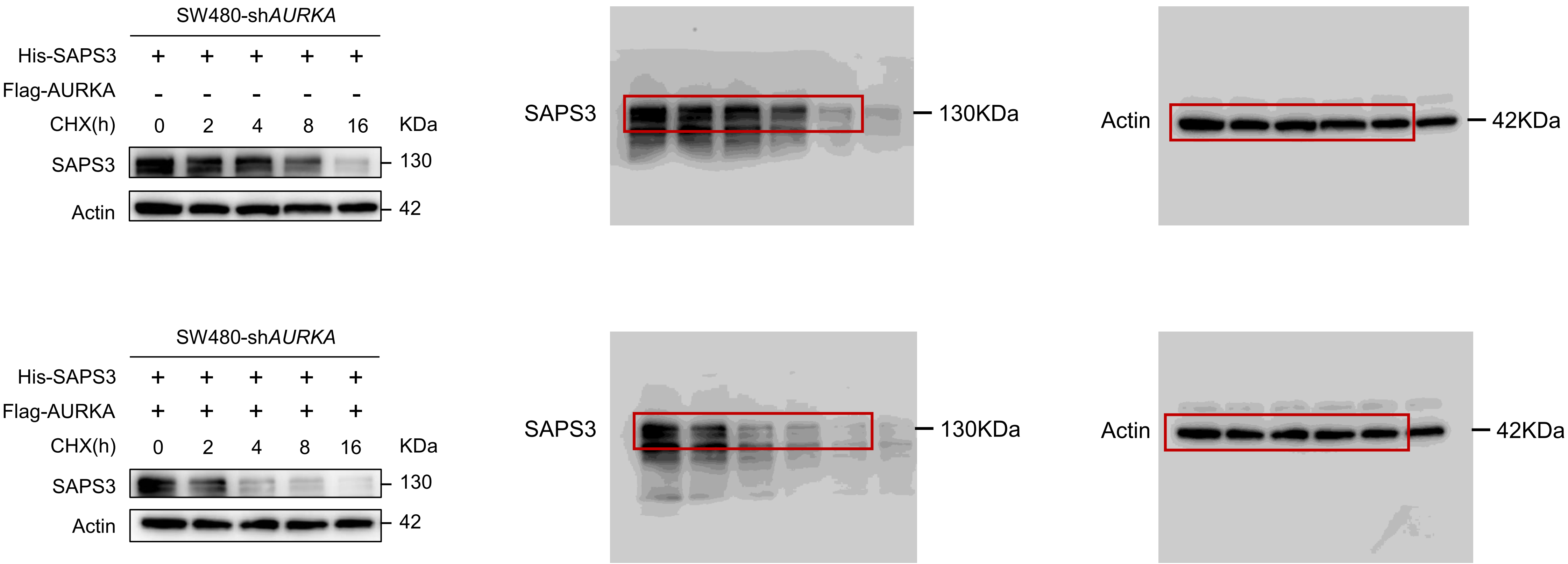
SW480-shAURKA
His-SAPS3 + + + + +
Flag-AURKA - - - - -
CHX(h) 0 2 4 8 16 KDa
SAPS3 130
Actin 42
SAPS3 130KDa
Actin 42KDa
SW480-shAURKA
His-SAPS3 + + + + +
Flag-AURKA + + + + +
CHX(h) 0 2 4 8 16 KDa
SAPS3 130
Actin 42
SAPS3 130KDa
Actin 42KDa

Fig.5N

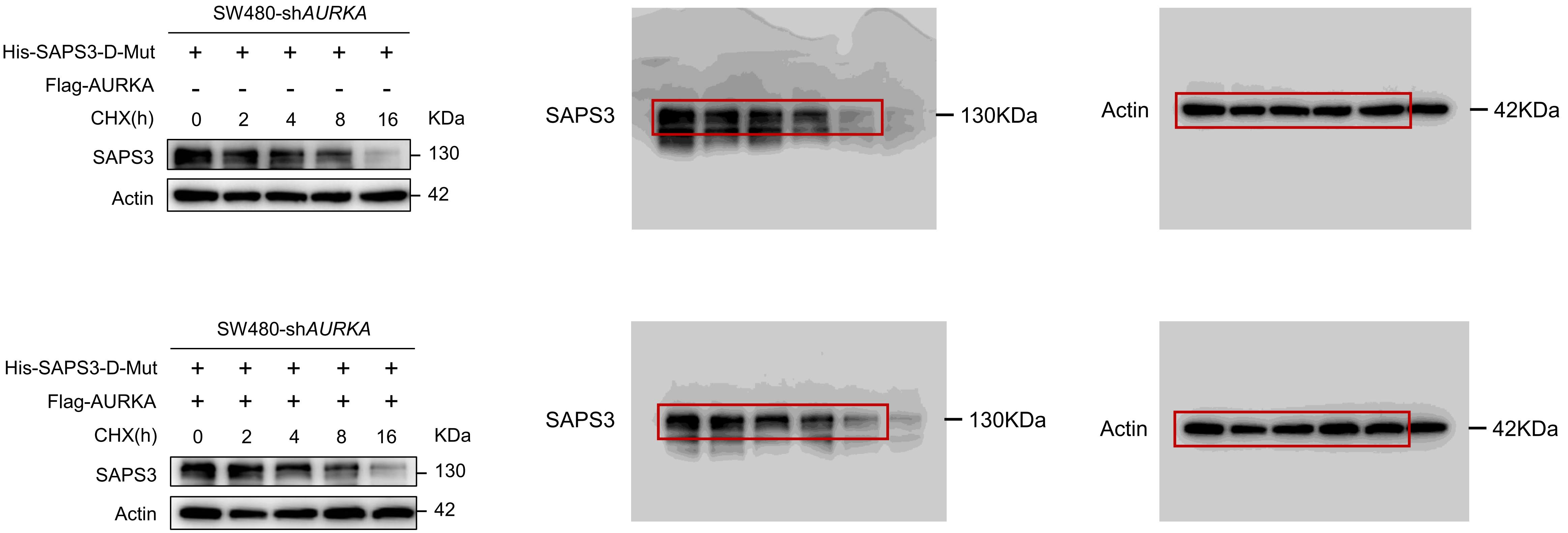
SW480-shAURKA
His-SAPS3-D-Mut + + + + +
Flag-AURKA - - - - -
CHX(h) 0 2 4 8 16 KDa
SAPS3 130
Actin 42
SAPS3
130KDa
Actin
42KDa
SW480-shAURKA
His-SAPS3-D-Mut + + + + +
Flag-AURKA + + + + +
CHX(h) 0 2 4 8 16 KDa
SAPS3 130
Actin 42
SAPS3
130KDa
Actin
42KDa

Fig.5P

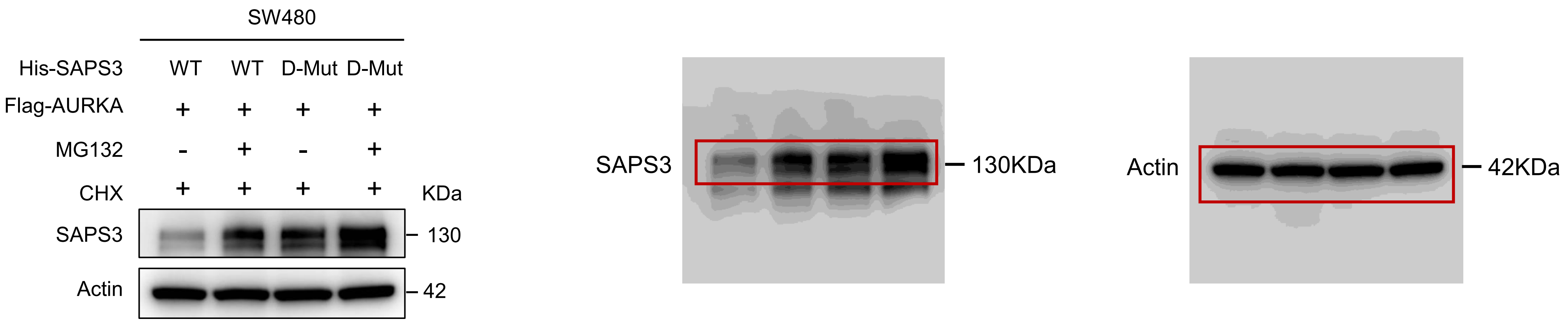
SW480
His-SAPS3
WT
WT
D-Mut
D-Mut
Flag-AURKA
+
+
+
+
MG132
-
+
-
+
CHX
+
+
+
+
KDa
SAPS3
130
Actin
42
SAPS3
130KDa
Actin
42KDa

Fig.5Q

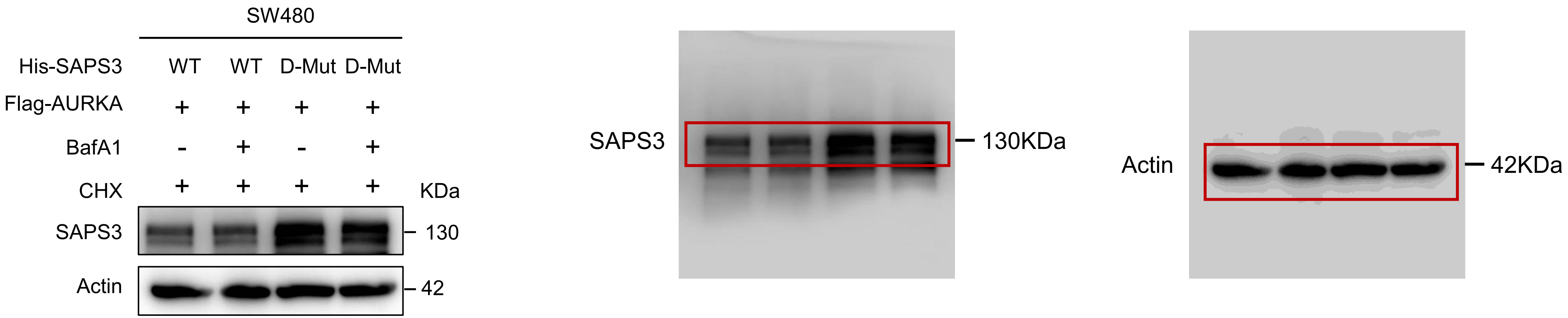
SW480
His-SAPS3
WT
WT
D-Mut
D-Mut
Flag-AURKA
+
+
+
+
BafA1
-
+
-
+
CHX
+
+
+
+
KDa
SAPS3
130
Actin
42
SAPS3
130KDa
Actin
42KDa

Fig.5R

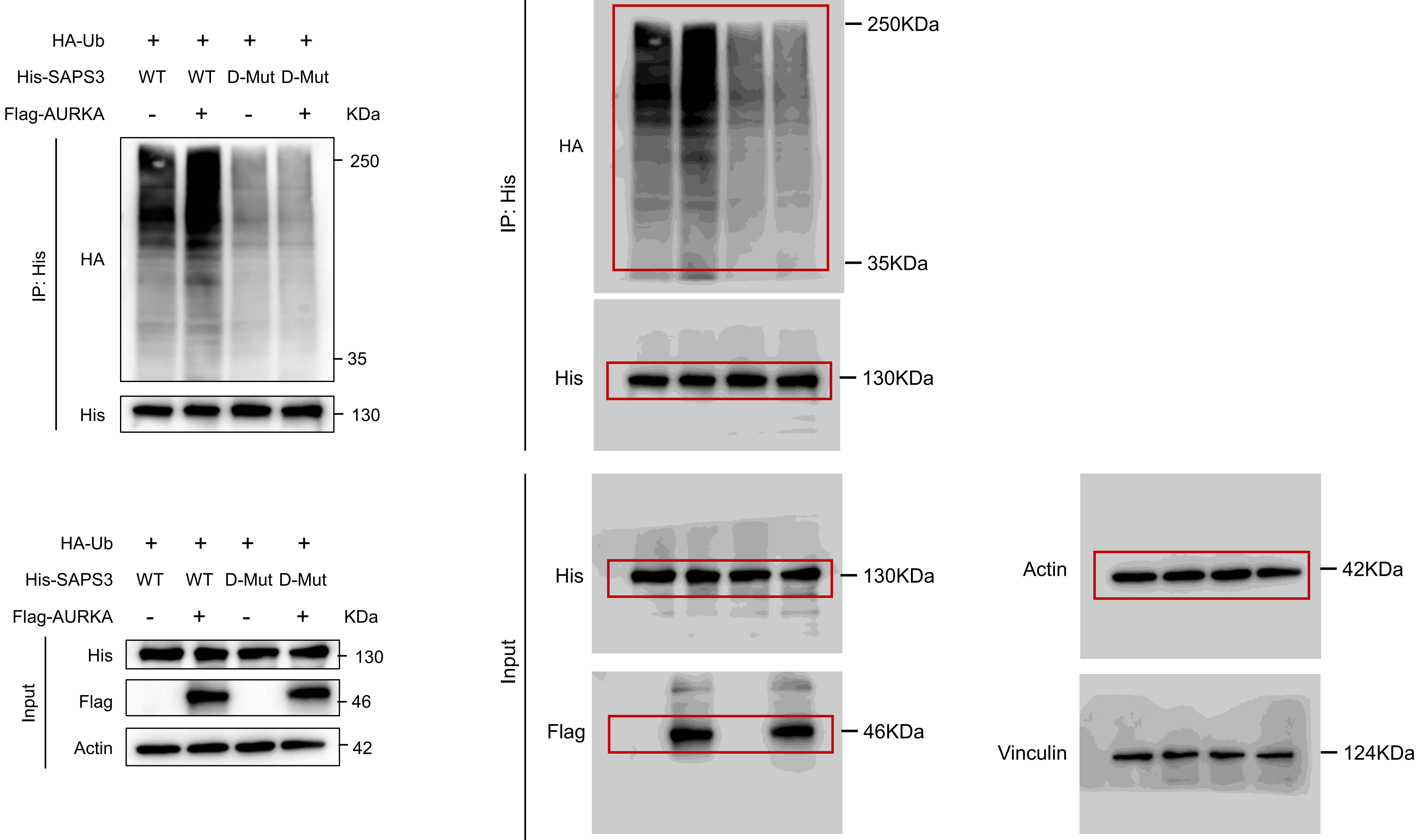
HA-Ub + + + +
His-SAPS3 WT WT D-Mut D-Mut
Flag-AURKA - + - + KDa
IP: His
HA
250
35
His
130
HA-Ub + + + +
His-SAPS3 WT WT D-Mut D-Mut
Flag-AURKA - + - + KDa
Input
His
130
Flag
46
Actin
42
IP: His
HA
250KDa
35KDa
His
130KDa
Input
His
130KDa
Flag
46KDa
Actin
42KDa
Vinculin
124KDa

Fig.6K

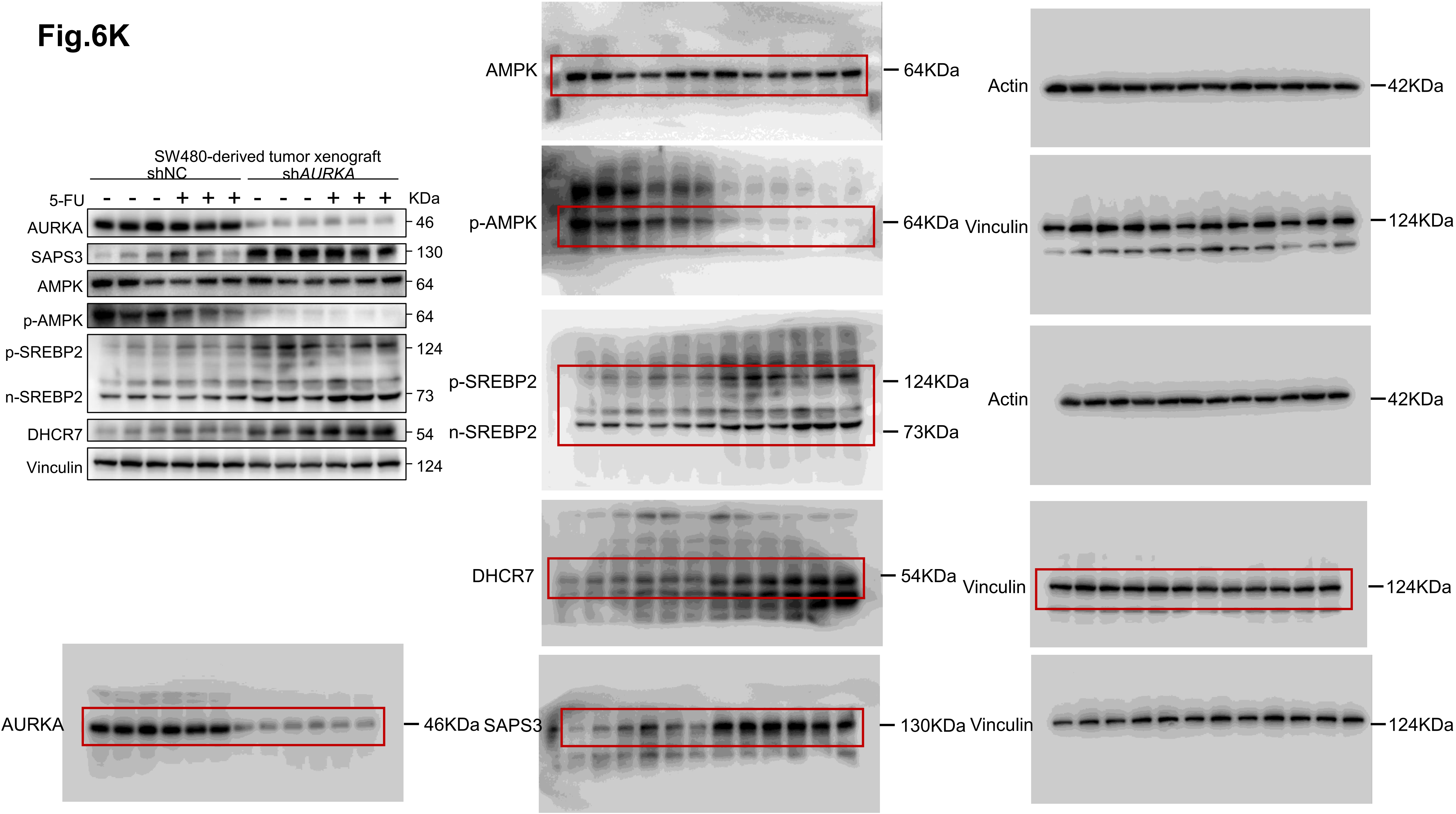
SW480-derived tumor xenograft
shNC
shAURKA
5-FU - - - + + + - - - + + +
KDa
AURKA 46
SAPS3 130
AMPK 64
p-AMPK 64
p-SREBP2 124
n-SREBP2 73
DHCR7 54
Vinculin 124
AMPK 64KDa
p-AMPK 64KDa
p-SREBP2 124KDa
n-SREBP2 73KDa
DHCR7 54KDa
AURKA 46KDa
SAPS3 130KDa
Actin 42KDa
Vinculin 124KDa
Actin 42KDa
Vinculin 124KDa
Vinculin 124KDa

Fig.7A

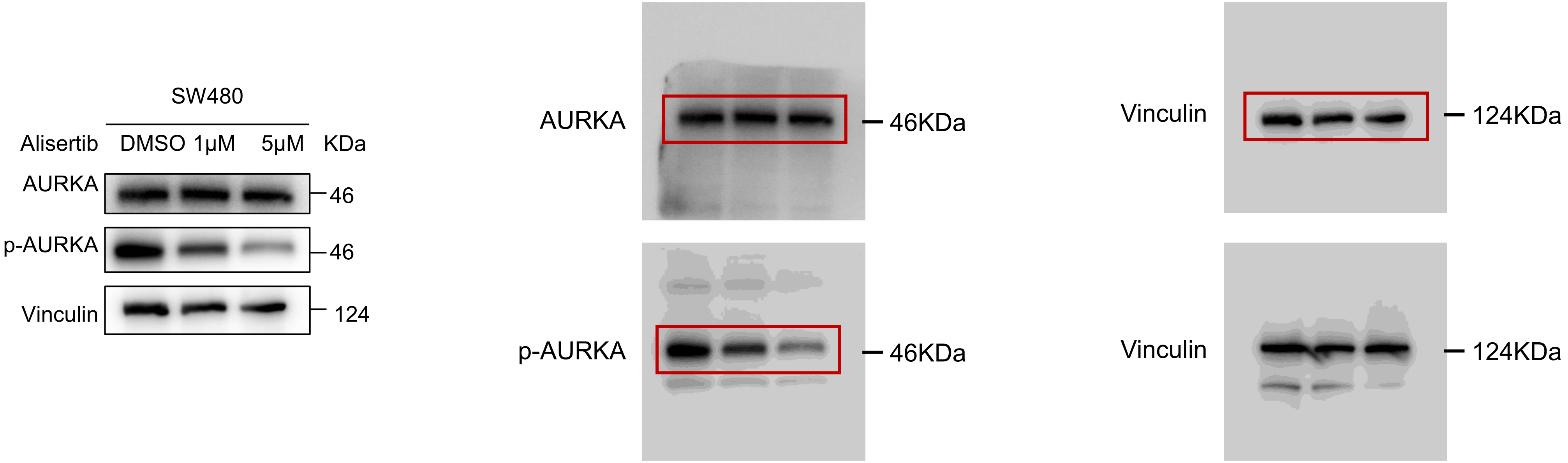

SW480
Alisertib
DMSO
1μM
5μM
KDa
AURKA
46
p-AURKA
46
Vinculin
124
AURKA
46KDa
p-AURKA
46KDa
Vinculin
124KDa
Vinculin
124KDa

Fig.7F

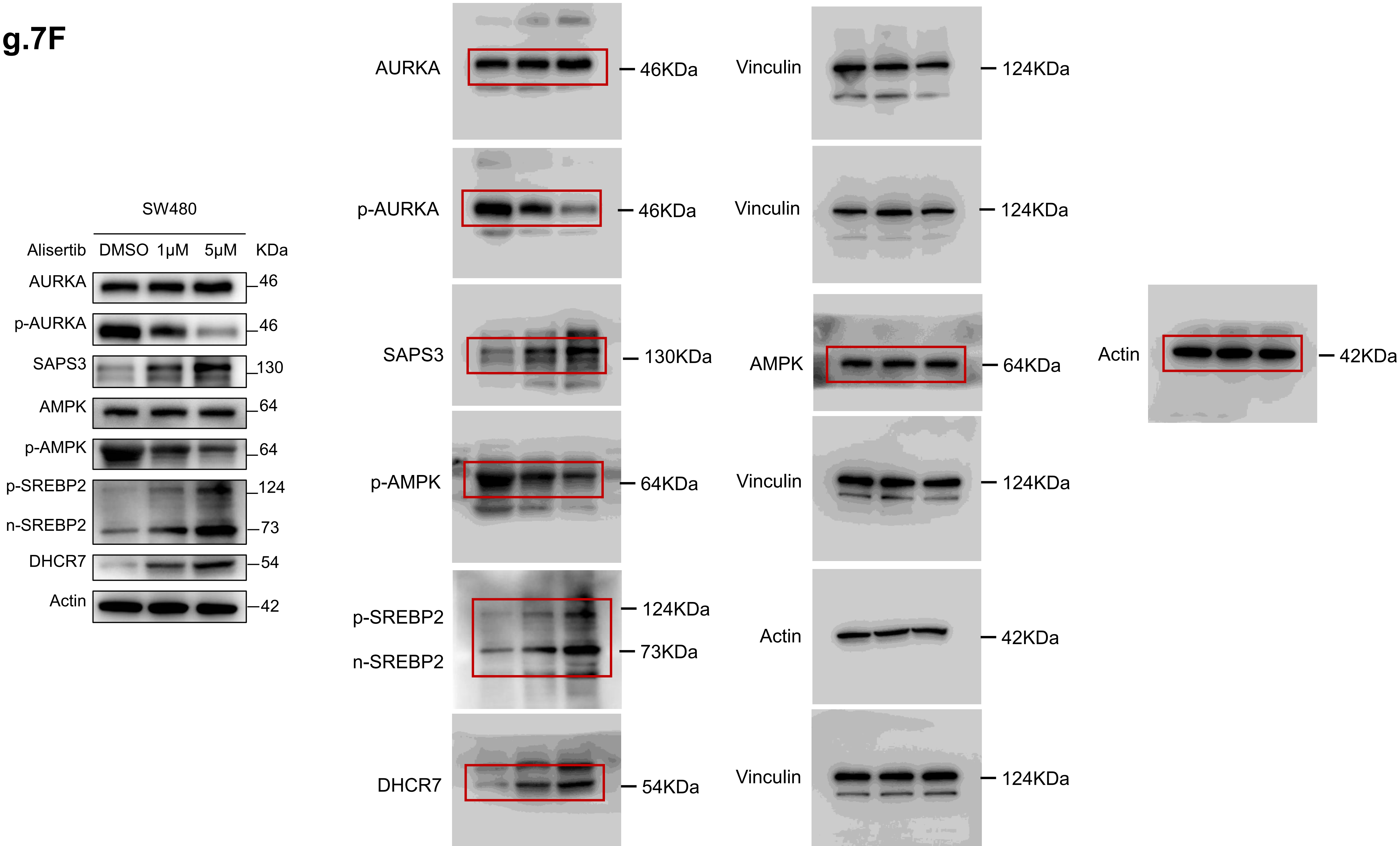
SW480
Alisertib DMSO 1μM 5μM KDa
AURKA 46
p-AURKA 46
SAPS3 130
AMPK 64
p-AMPK 64
p-SREBP2 124
n-SREBP2 73
DHCR7 54
Actin 42
AURKA 46KDa
p-AURKA 46KDa
SAPS3 130KDa
p-AMPK 64KDa
p-SREBP2 124KDa
n-SREBP2 73KDa
DHCR7 54KDa
Vinculin 124KDa
Vinculin 124KDa
AMPK 64KDa
Vinculin 124KDa
Actin 42KDa
Vinculin 124KDa
Actin 42KDa

Fig.7N

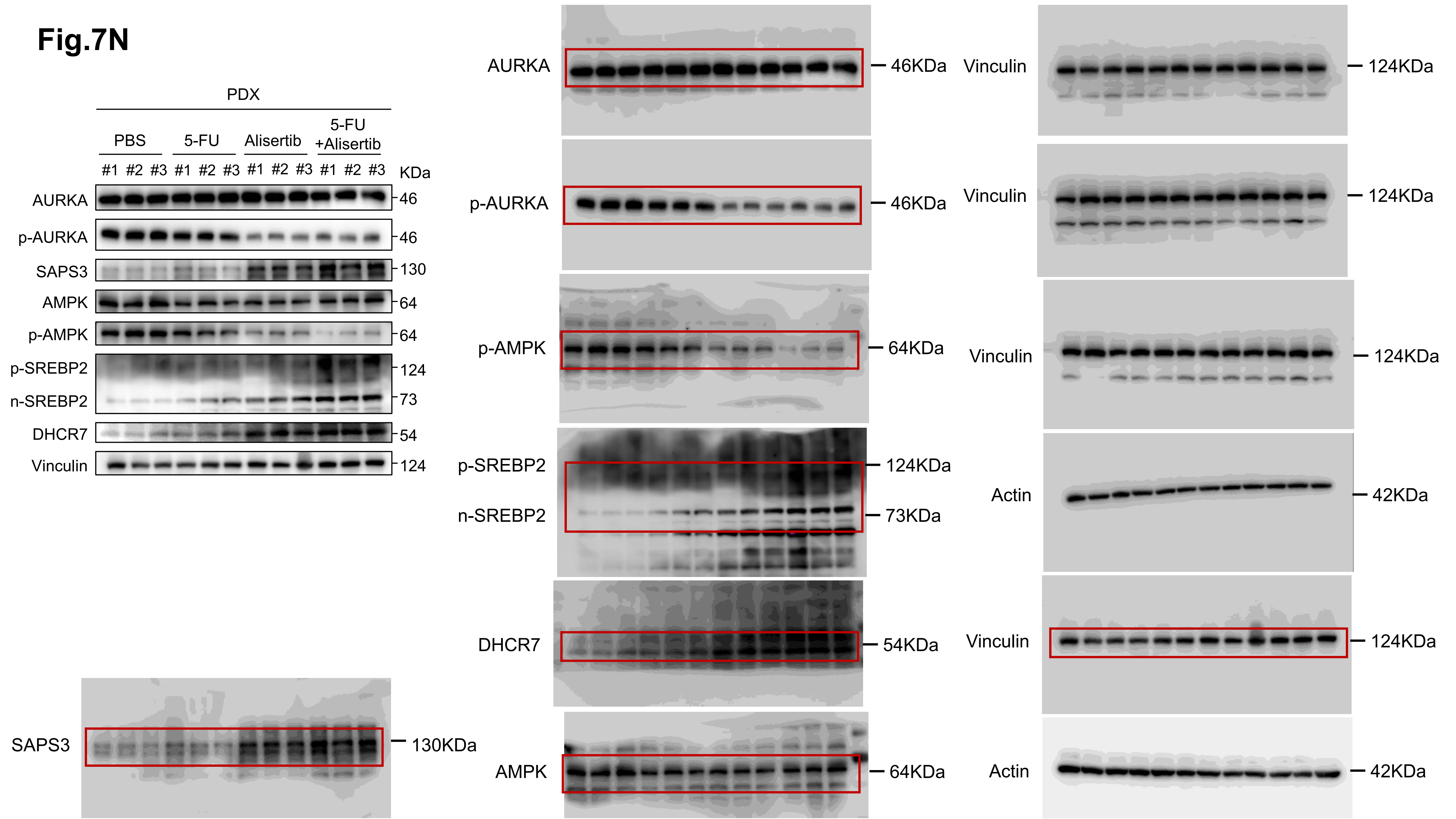
PDX
PBS
5-FU
Alisertib
5-FU
+Alisertib
#1 #2 #3 #1 #2 #3 #1 #2 #3 #1 #2 #3
KDa
AURKA
p-AURKA
SAPS3
AMPK
p-AMPK
p-SREBP2
n-SREBP2
DHCR7
Vinculin
46
46
130
64
64
124
73
54
124
AURKA
46KDa
p-AURKA
46KDa
p-AMPK
64KDa
p-SREBP2
124KDa
n-SREBP2
73KDa
DHCR7
54KDa
AMPK
64KDa
SAPS3
130KDa
Vinculin
124KDa
Vinculin
124KDa
Vinculin
124KDa
Actin
42KDa
Vinculin
124KDa
Actin
42KDa

SFig.2A

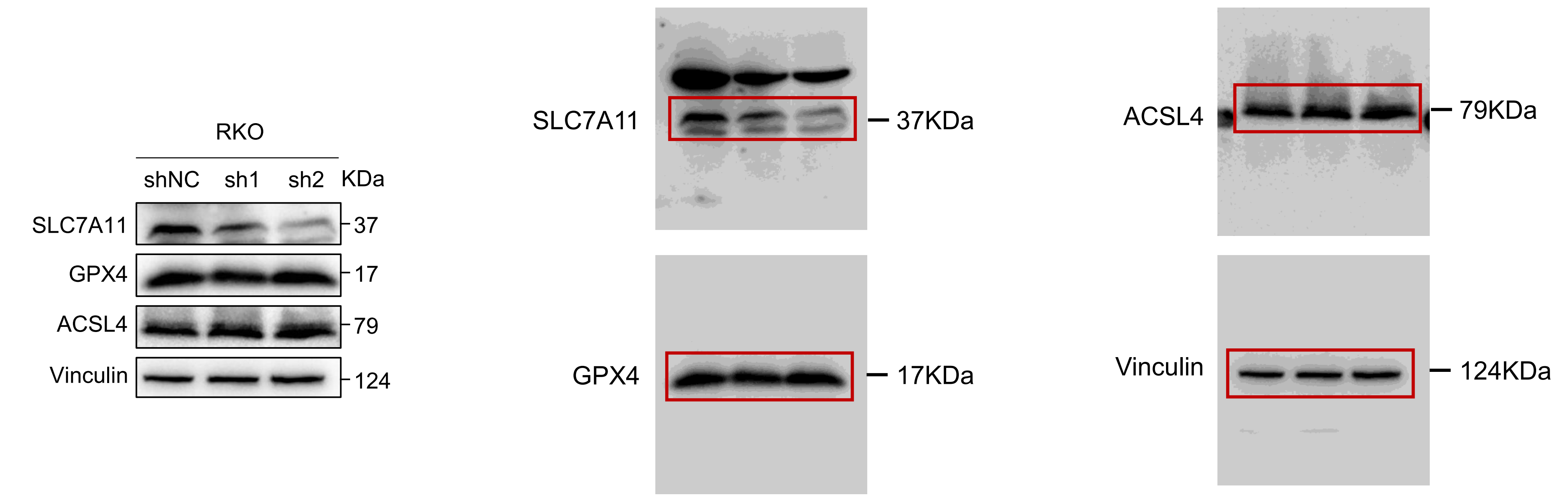

SFig.2C

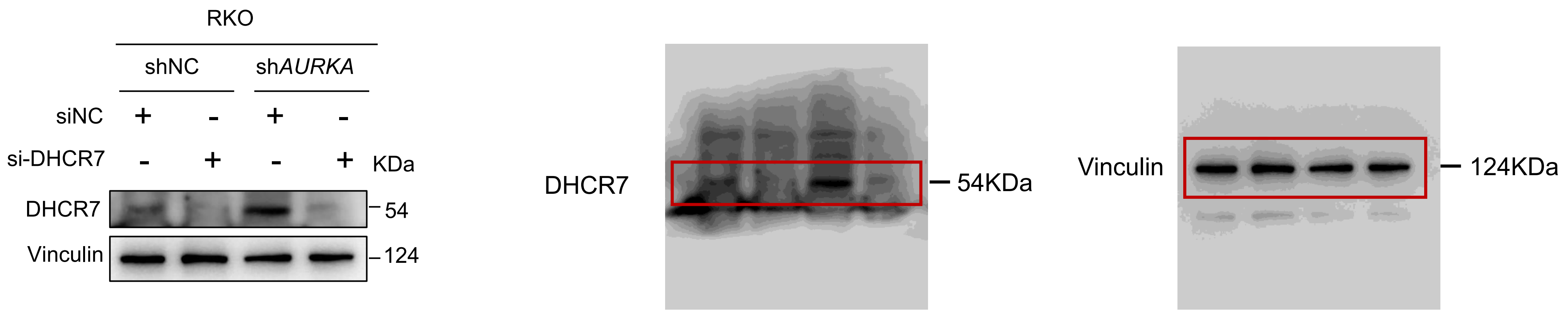

SFig.3B

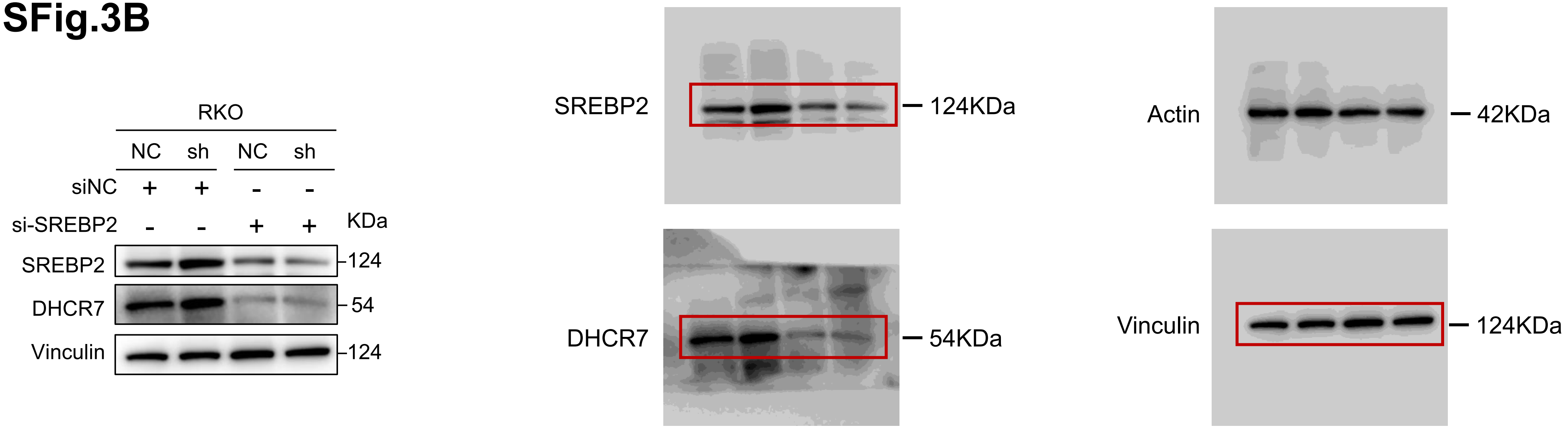

SFig.3F

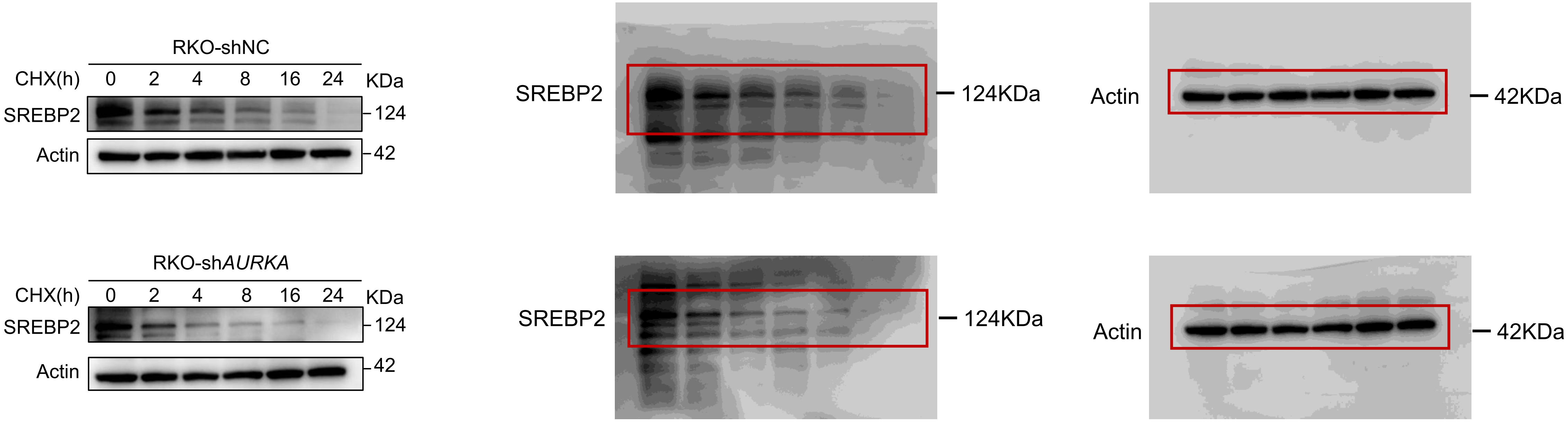

SFig.3H

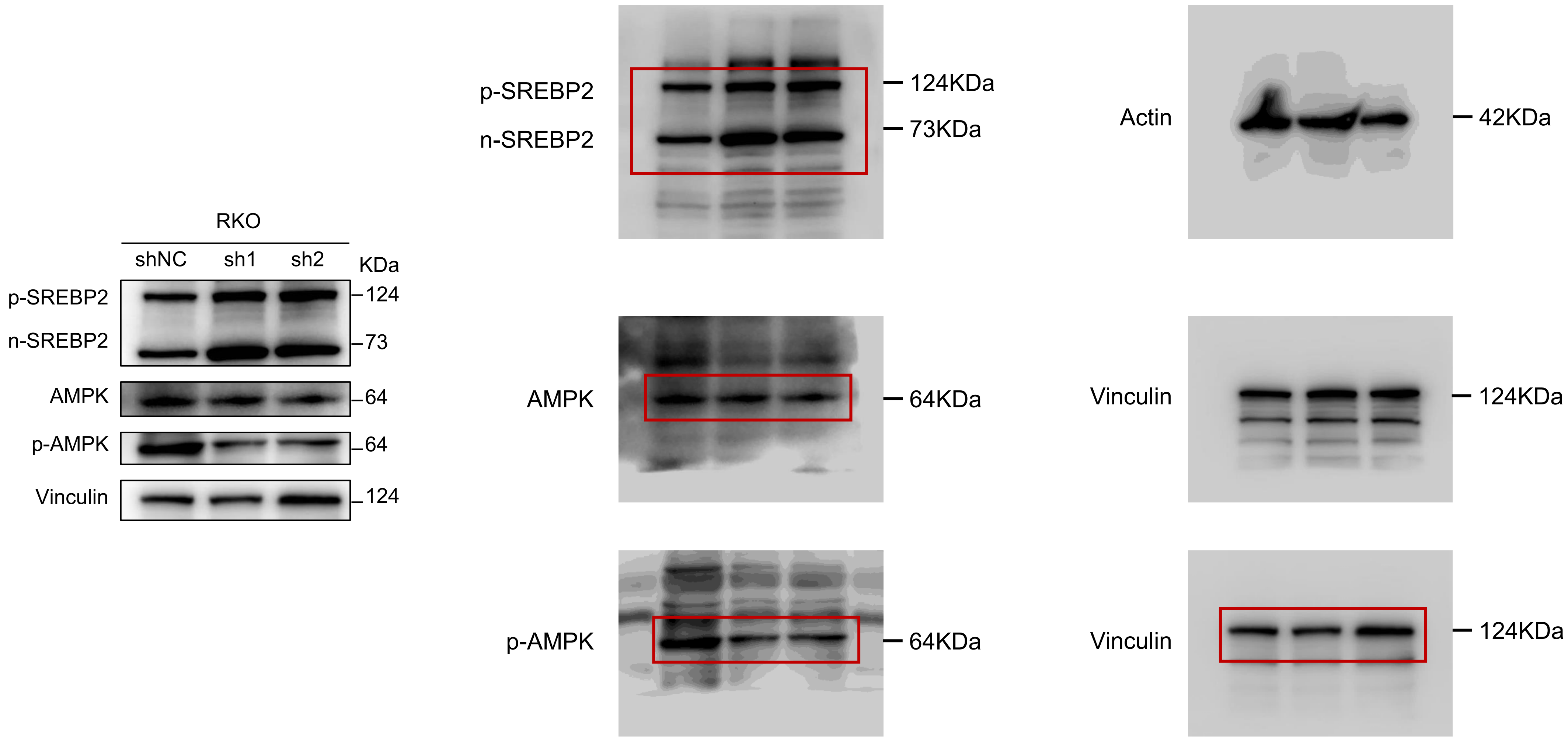
RKO
shNC
sh1
sh2
KDa
p-SREBP2
n-SREBP2
124
73
AMPK
64
p-AMPK
64
Vinculin
124
p-SREBP2
n-SREBP2
124KDa
73KDa
Actin
42KDa
AMPK
64KDa
Vinculin
124KDa
p-AMPK
64KDa
Vinculin
124KDa

SFig.3I

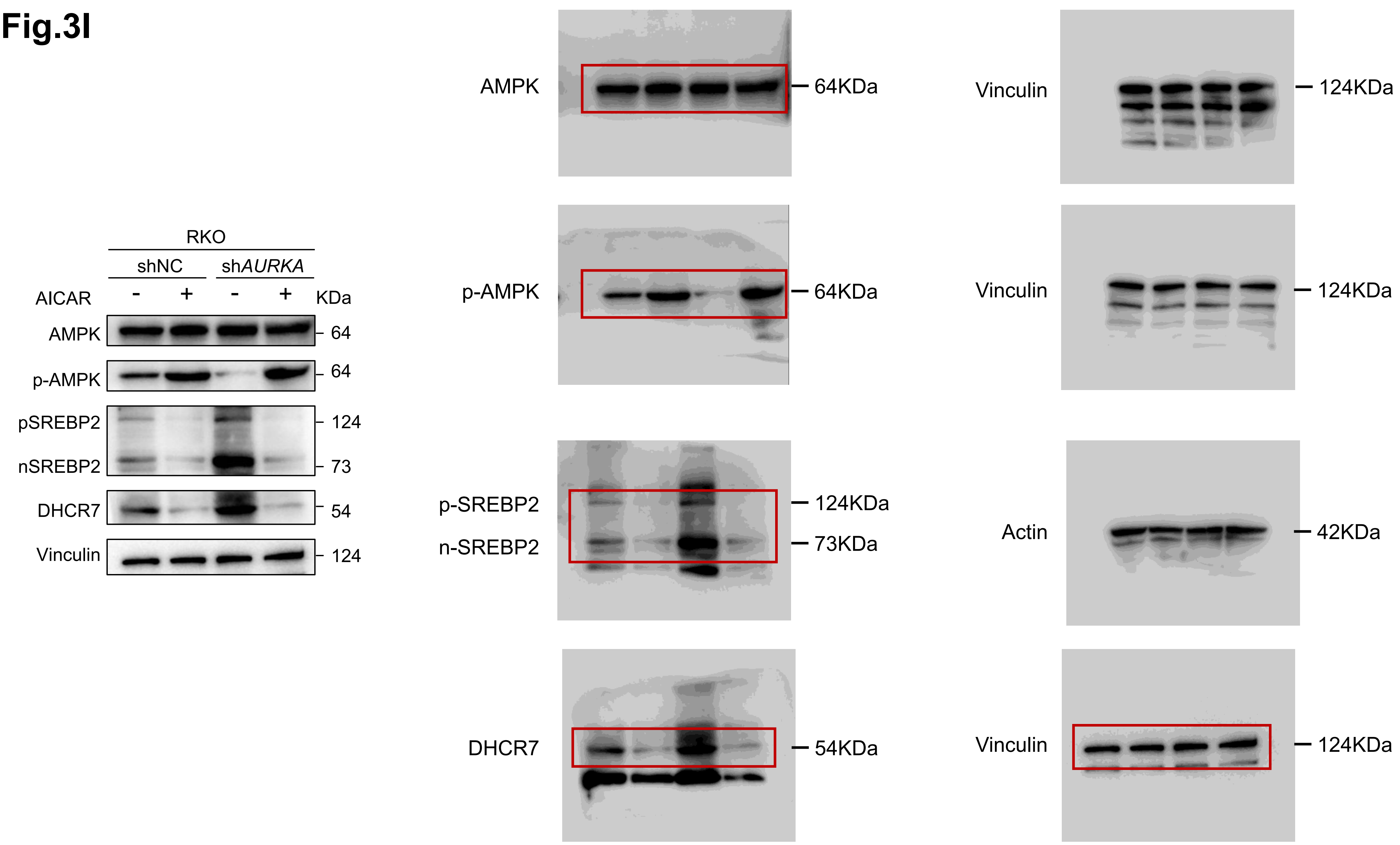
RKO
shNC
shAURKA
AICAR
-
+
-
+
KDa
AMPK
64
p-AMPK
64
pSREBP2
124
nSREBP2
73
DHCR7
54
Vinculin
124
AMPK
64KDa
p-AMPK
64KDa
p-SREBP2
n-SREBP2
124KDa
73KDa
DHCR7
54KDa
Vinculin
124KDa
Vinculin
124KDa
Actin
42KDa
Vinculin
124KDa

SFig.4A

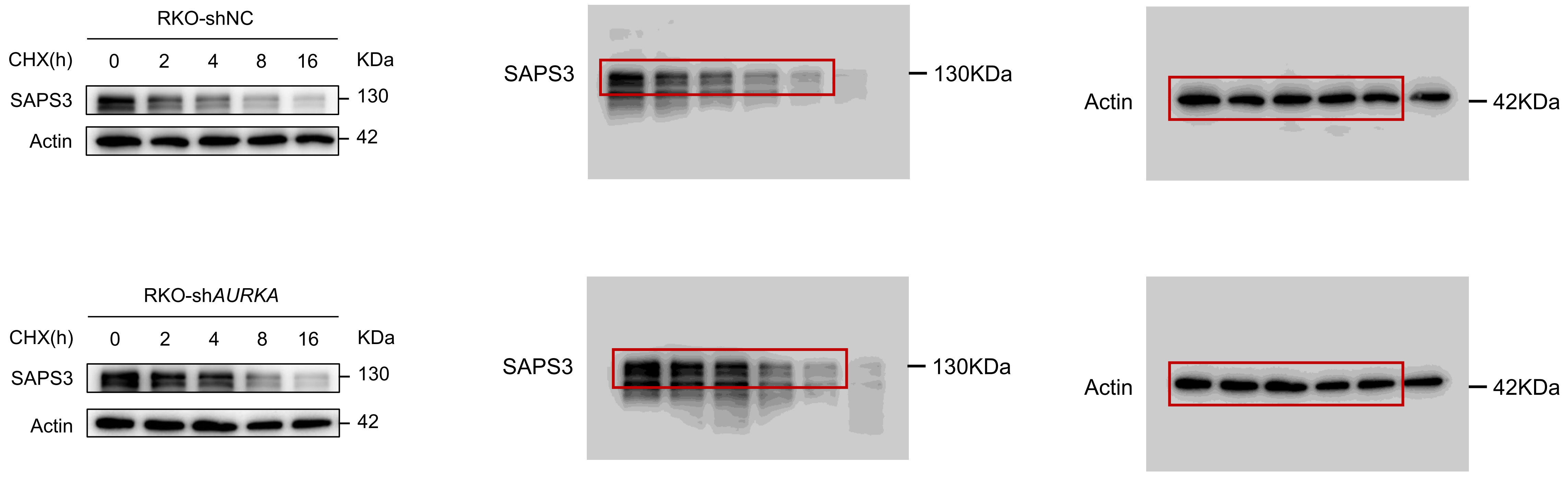
RKO-shNC
CHX(h) 0 2 4 8 16 KDa
SAPS3 130
Actin 42
RKO-sh*AURKA*
CHX(h) 0 2 4 8 16 KDa
SAPS3 130
Actin 42
SAPS3
130KDa
Actin
42KDa
SAPS3
130KDa
Actin
42KDa

SFig.4E

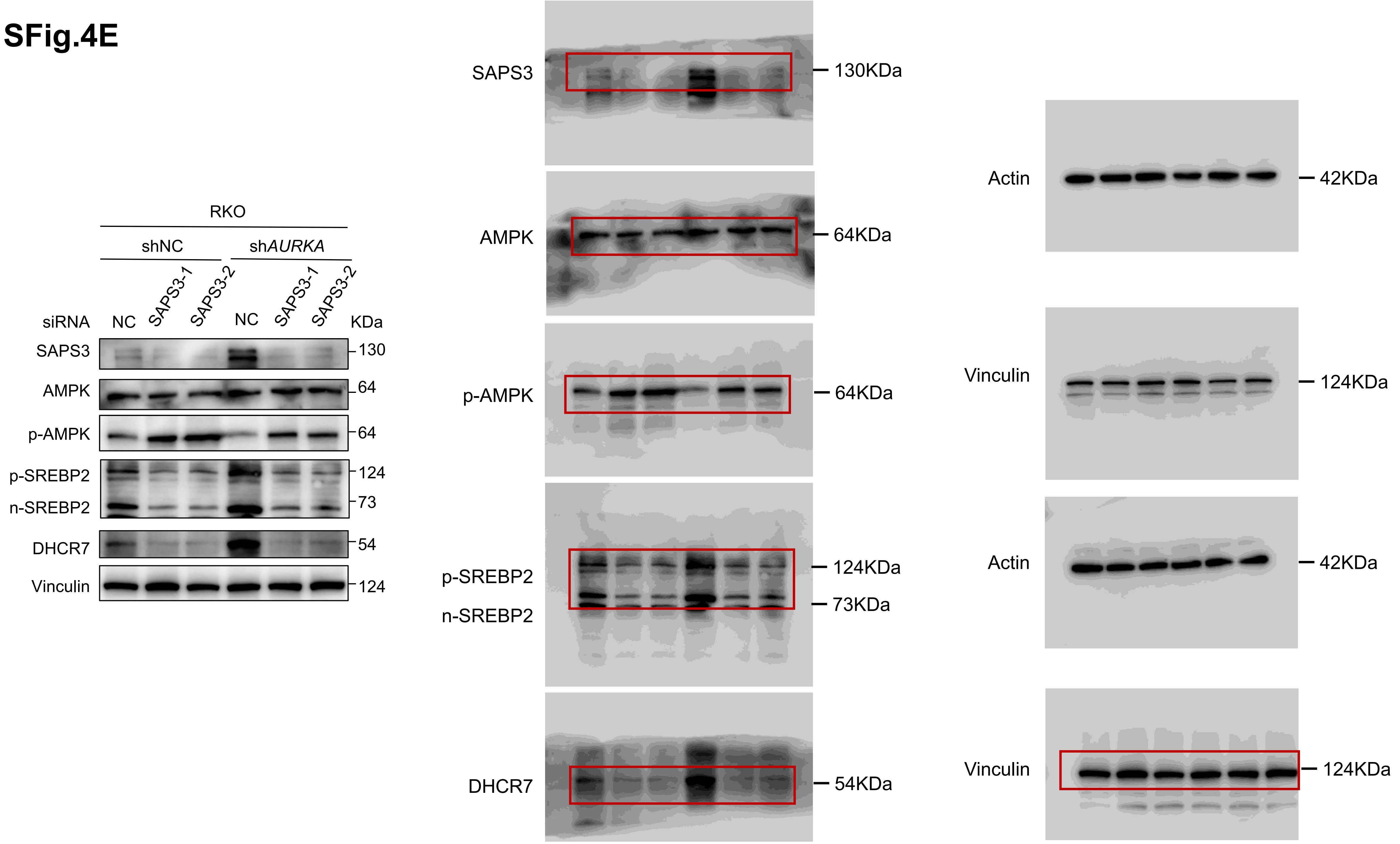
RKO
shNC
shAURKA
siRNA
NC
SAPS3-1
SAPS3-2
NC
SAPS3-1
SAPS3-2
KDa
SAPS3
130
AMPK
64
p-AMPK
64
p-SREBP2
124
n-SREBP2
73
DHCR7
54
Vinculin
124
SAPS3
130KDa
AMPK
64KDa
p-AMPK
64KDa
p-SREBP2
124KDa
n-SREBP2
73KDa
DHCR7
54KDa
Actin
42KDa
Vinculin
124KDa
Actin
42KDa
Vinculin
124KDa

SFig.5A

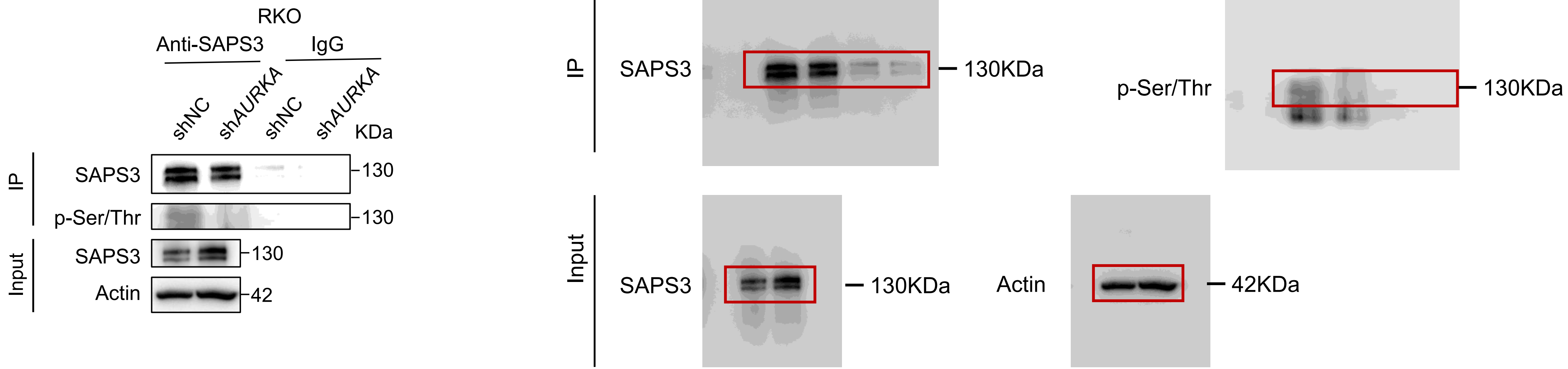

SFig.5B

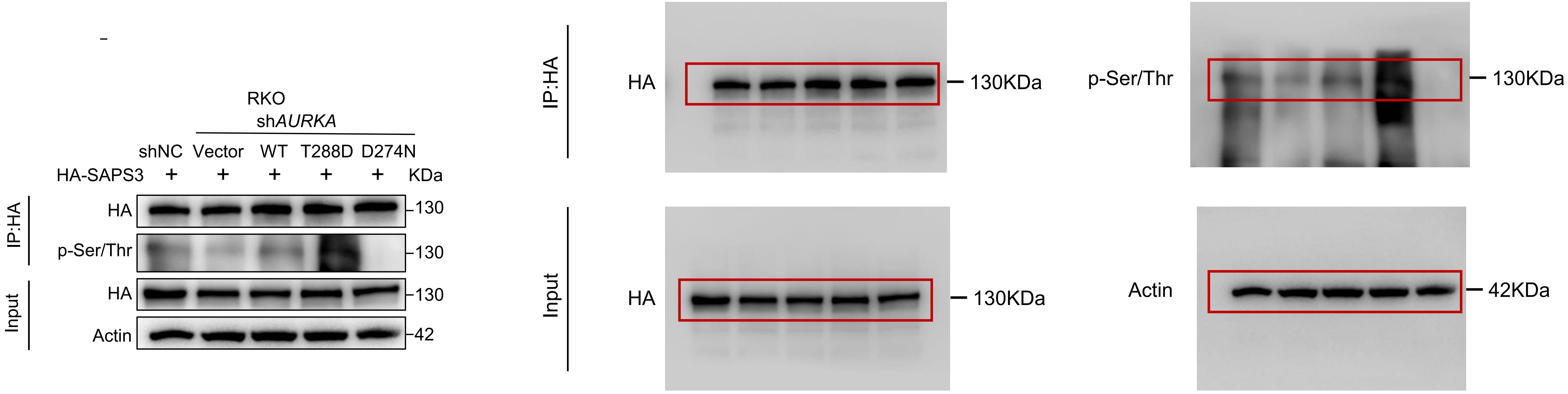

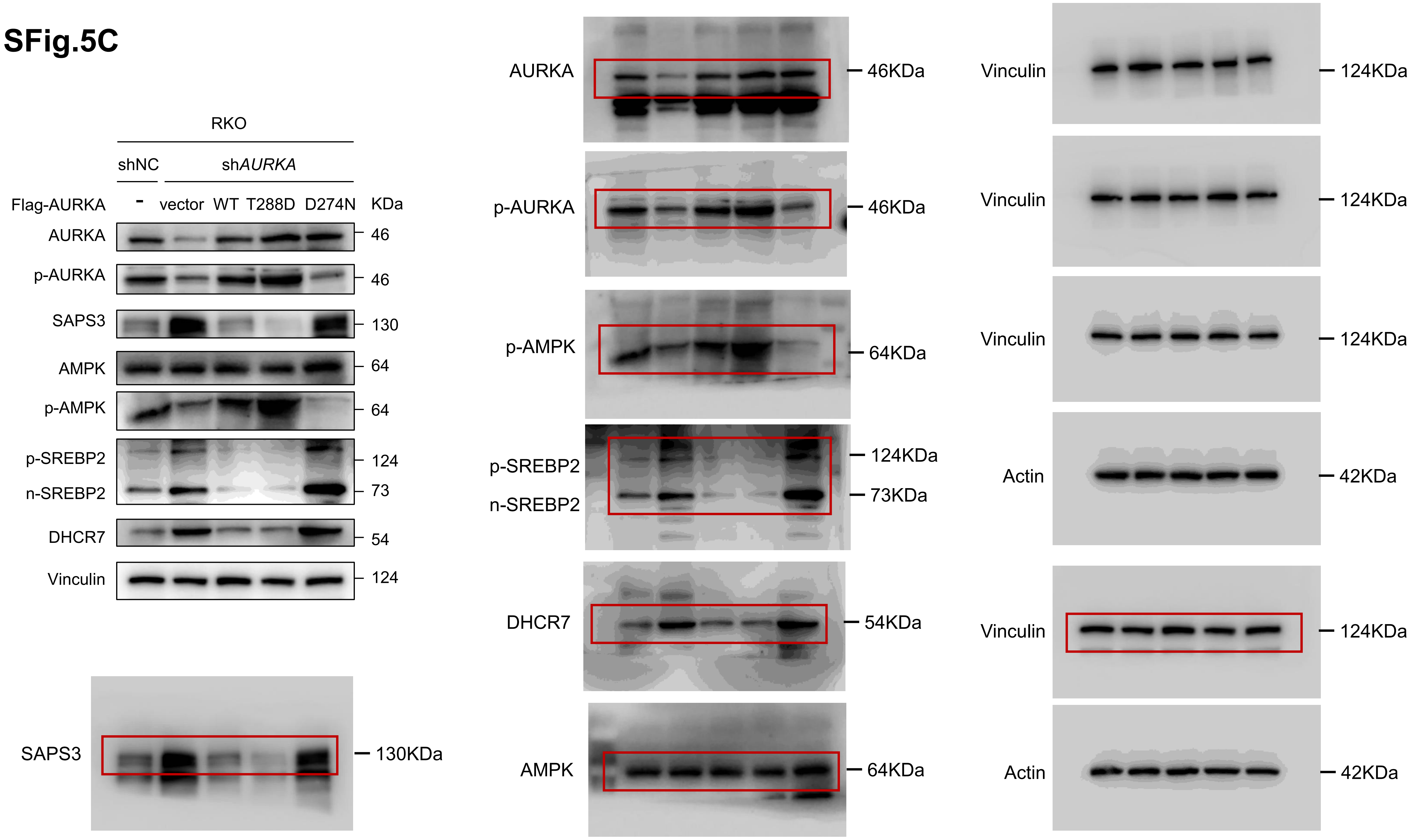

SFig.5C
RKO
shNC
shAURKA
Flag-AURKA
-
vector
WT
T288D
D274N
KDa
AURKA
46
p-AURKA
46
SAPS3
130
AMPK
64
p-AMPK
64
p-SREBP2
124
n-SREBP2
73
DHCR7
54
Vinculin
124
SAPS3
130KDa
AURKA
46KDa
p-AURKA
46KDa
p-AMPK
64KDa
p-SREBP2
124KDa
n-SREBP2
73KDa
DHCR7
54KDa
AMPK
64KDa
Vinculin
124KDa
Vinculin
124KDa
Vinculin
124KDa
Actin
42KDa
Vinculin
124KDa
Actin
42KDa

SFig.5F

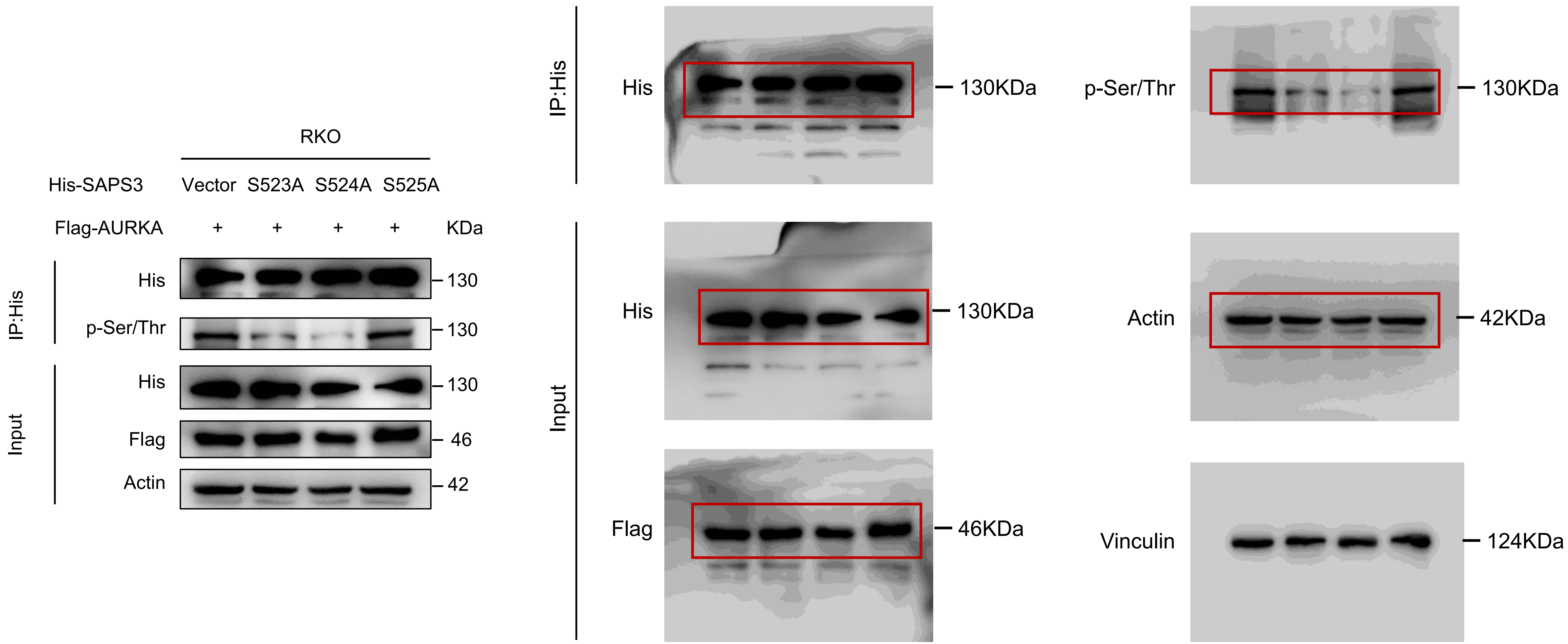
RKO
His-SAPS3
Vector
S523A
S524A
S525A
Flag-AURKA
+
+
+
+
KDa
IP:His
His
130
p-Ser/Thr
130
Input
His
130
Flag
46
Actin
42
IP:His
His
130KDa
p-Ser/Thr
130KDa
Input
His
130KDa
Actin
42KDa
Flag
46KDa
Vinculin
124KDa

SFig.5G

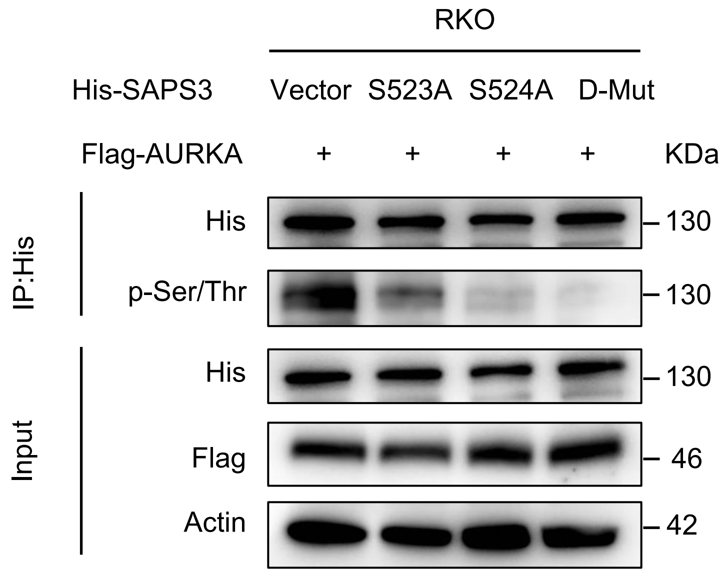

RKO
His-SAPS3
Vector
S523A
S524A
D-Mut
Flag-AURKA
+
+
+
+
KDa
IP:His
His
130
p-Ser/Thr
130
Input
His
130
Flag
46
Actin
42

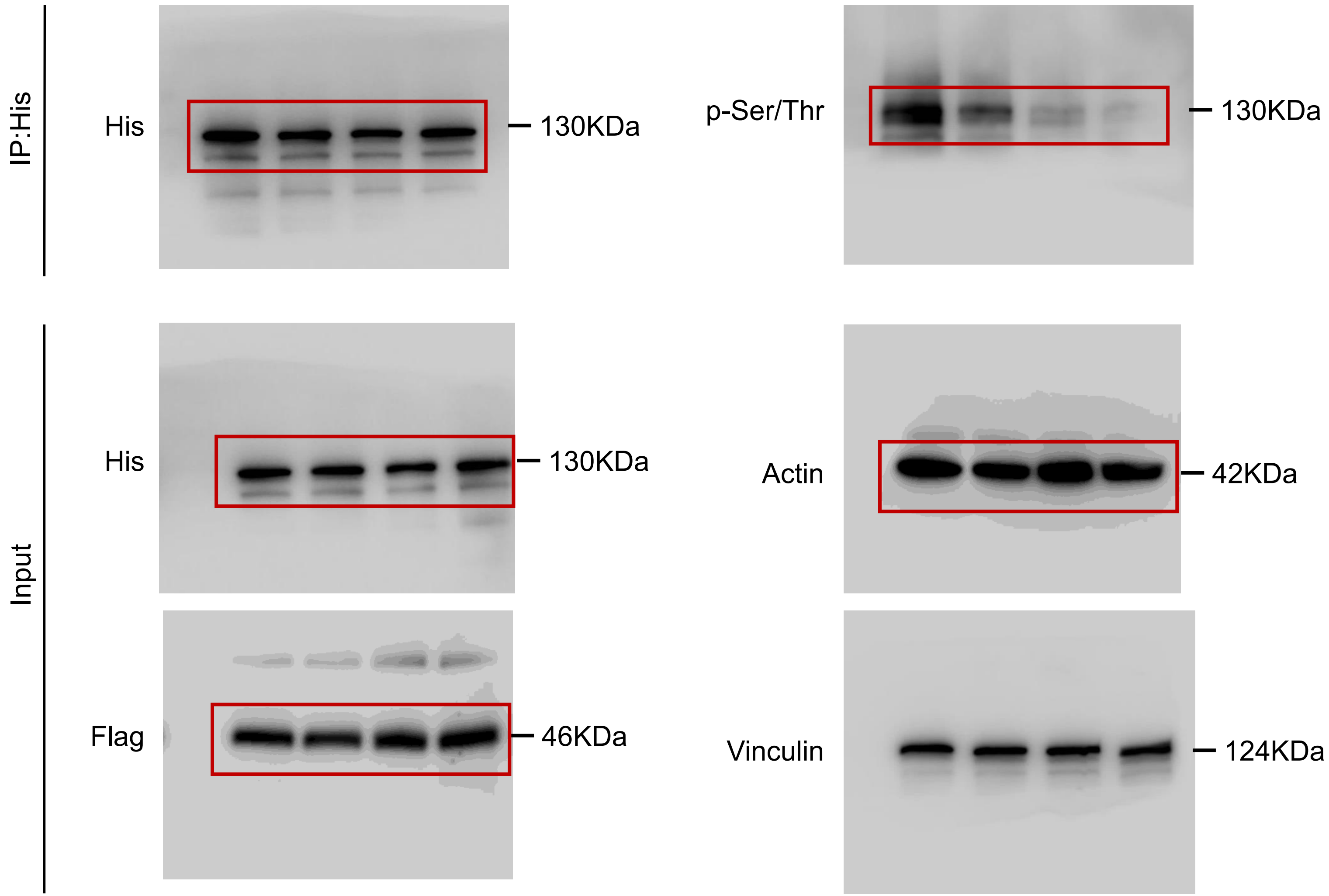

IP:His
His
130KDa
p-Ser/Thr
130KDa
Input
His
130KDa
Actin
42KDa
Flag
46KDa
Vinculin
124KDa

SFig.5H

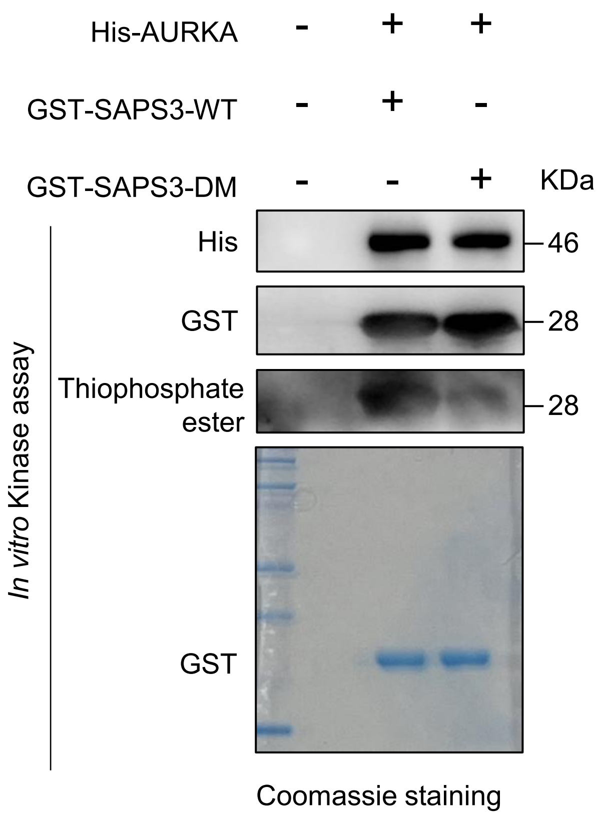

His-AURKA - + +
GST-SAPS3-WT - + -
GST-SAPS3-DM - - +
KDa
His
46
GST
28
Thiophosphate ester
28
GST
In vitro Kinase assay
Coomassie staining

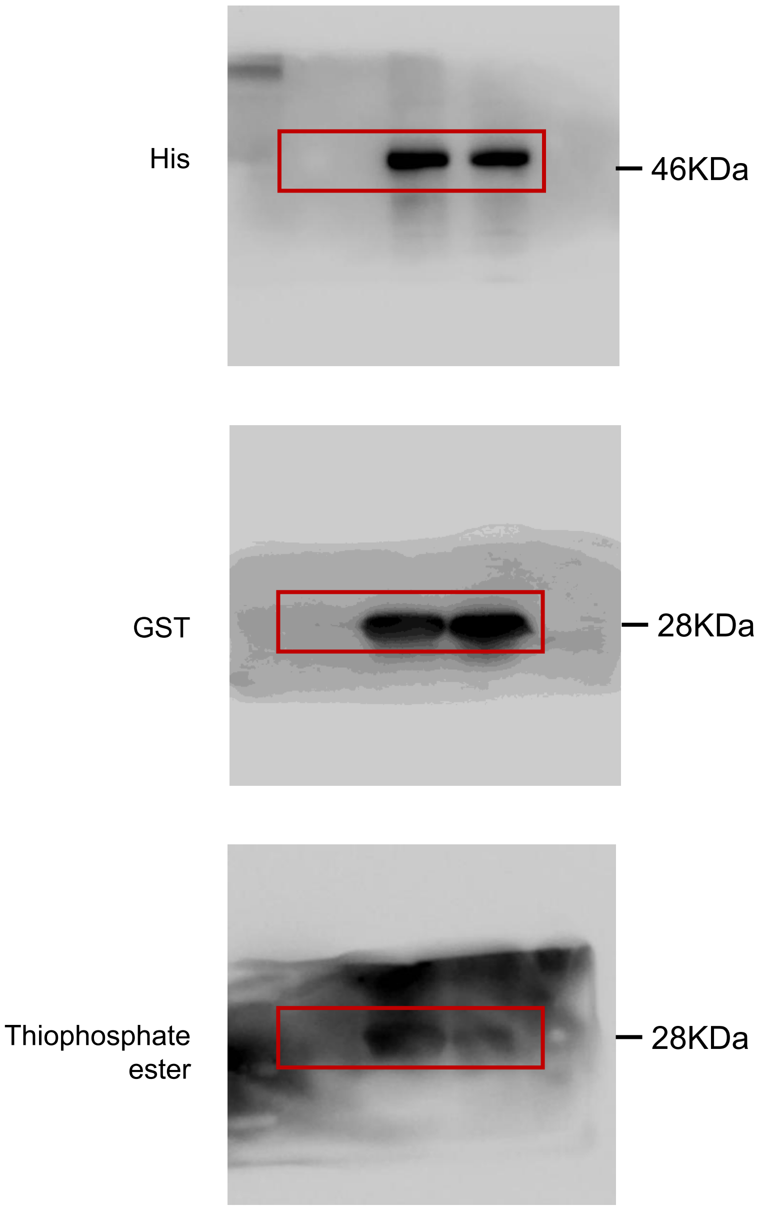

His
46KDa
GST
28KDa
Thiophosphate ester
28KDa

SFig.5l

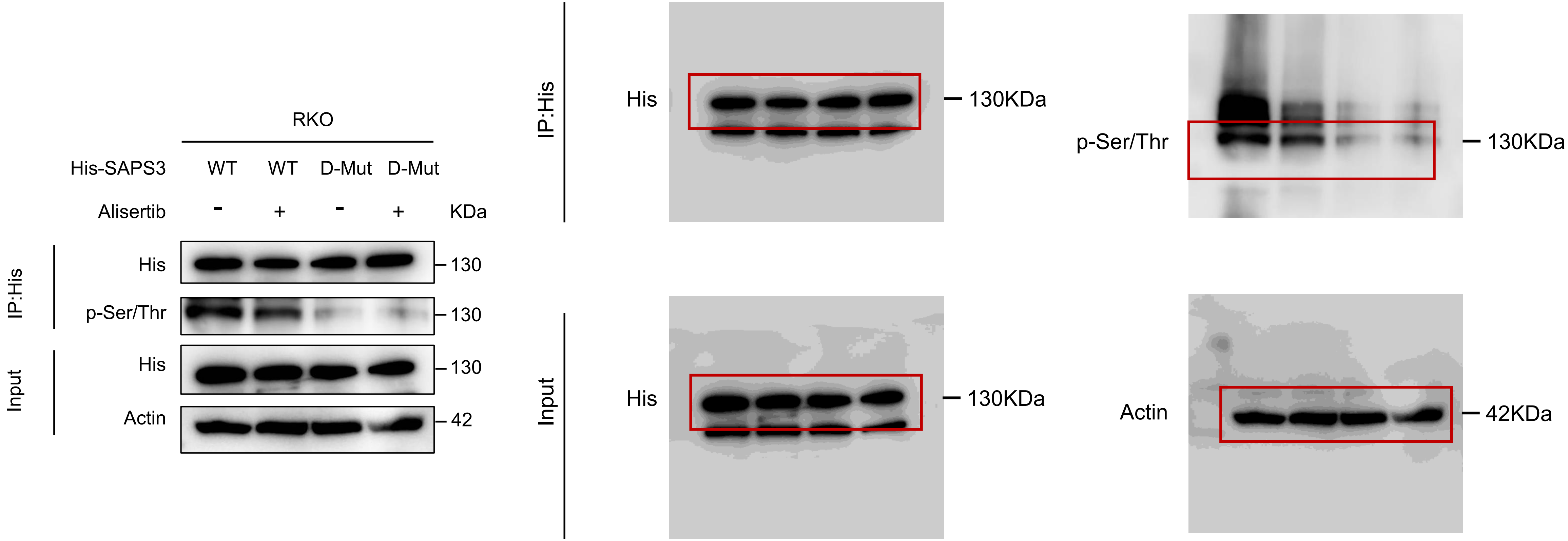
RKO
His-SAPS3
WT
WT
D-Mut
D-Mut
Alisertib
-
+
-
+
KDa
IP:His
His
130
p-Ser/Thr
130
Input
His
130
Actin
42
IP:His
His
130KDa
p-Ser/Thr
130KDa
Input
His
130KDa
Actin
42KDa

SFig.5J

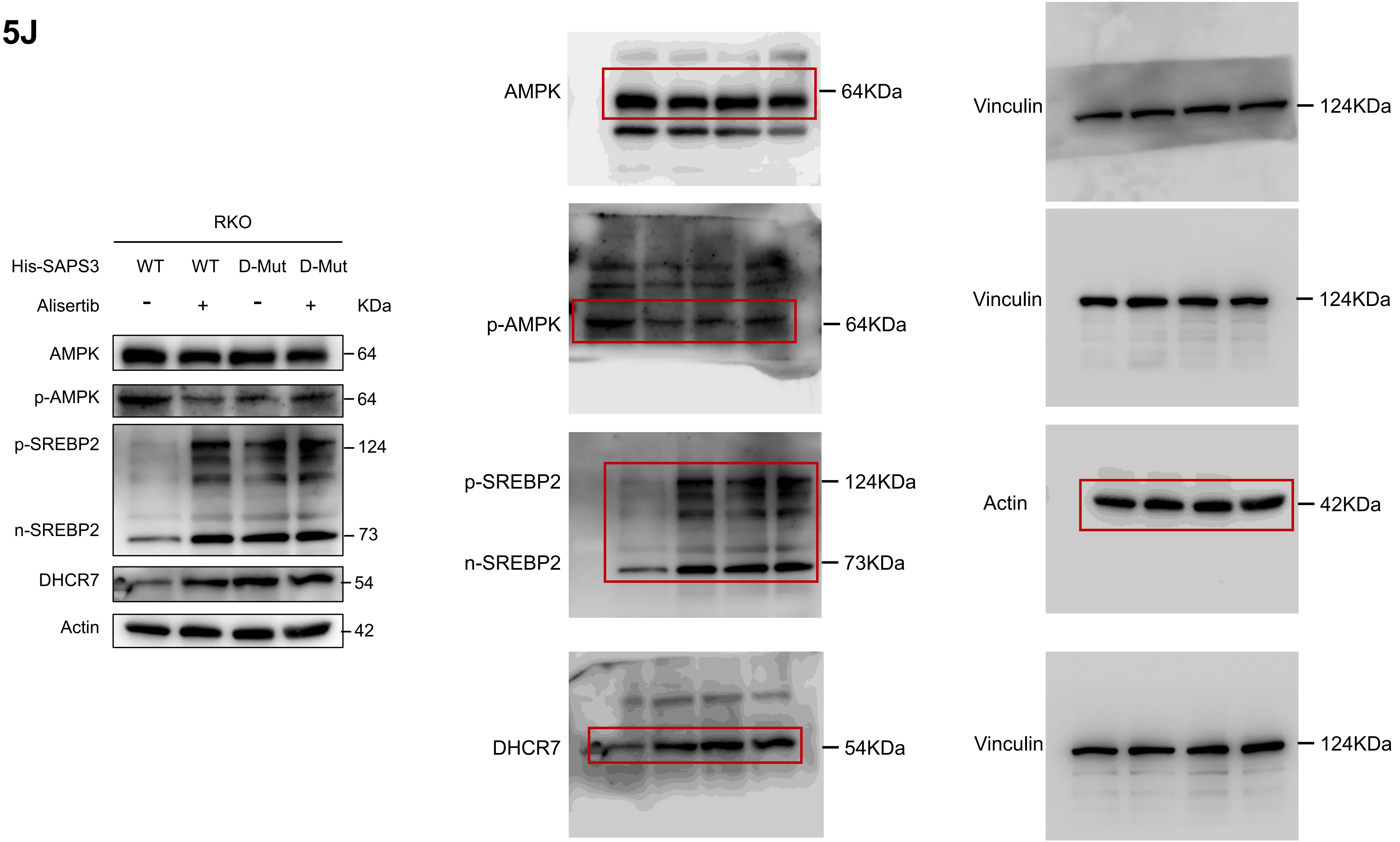
RKO
His-SAPS3
WT
WT
D-Mut
D-Mut
Alisertib
-
+
-
+
KDa
AMPK
64
p-AMPK
64
p-SREBP2
124
n-SREBP2
73
DHCR7
54
Actin
42
AMPK
64KDa
p-AMPK
64KDa
p-SREBP2
124KDa
n-SREBP2
73KDa
DHCR7
54KDa
Vinculin
124KDa
Vinculin
124KDa
Actin
42KDa
Vinculin
124KDa

SFig.5K

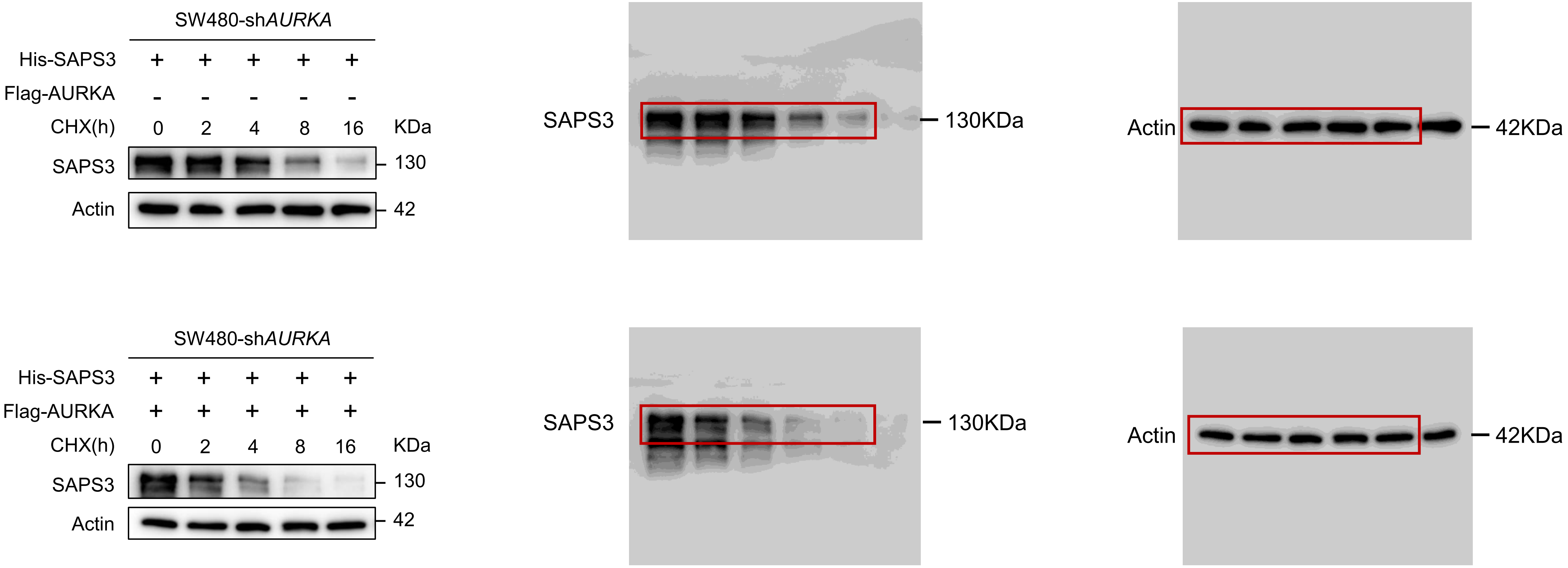
SW480-shAURKA
His-SAPS3 + + + + +
Flag-AURKA - - - - -
CHX(h) 0 2 4 8 16
KDa
SAPS3
130
Actin
42
SAPS3
130KDa
Actin
42KDa
SW480-shAURKA
His-SAPS3 + + + + +
Flag-AURKA + + + + +
CHX(h) 0 2 4 8 16
KDa
SAPS3
130
Actin
42
SAPS3
130KDa
Actin
42KDa

SFig.5M

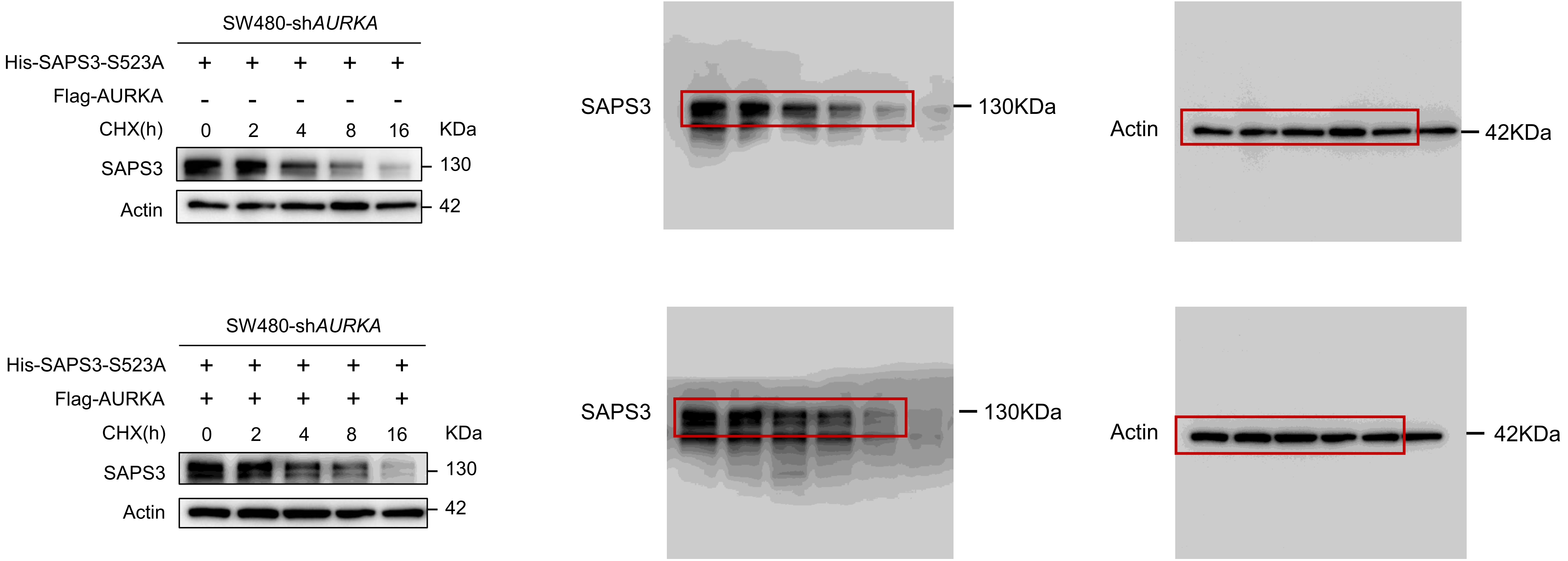

SW480-shAURKA
His-SAPS3-S523A + + + + +
Flag-AURKA - - - - -
CHX(h) 0 2 4 8 16
KDa
SAPS3
130
Actin
42
SAPS3
130KDa
Actin
42KDa
SW480-shAURKA
His-SAPS3-S523A + + + + +
Flag-AURKA + + + + +
CHX(h) 0 2 4 8 16
KDa
SAPS3
130
Actin
42
SAPS3
130KDa
Actin
42KDa

SFig.5O

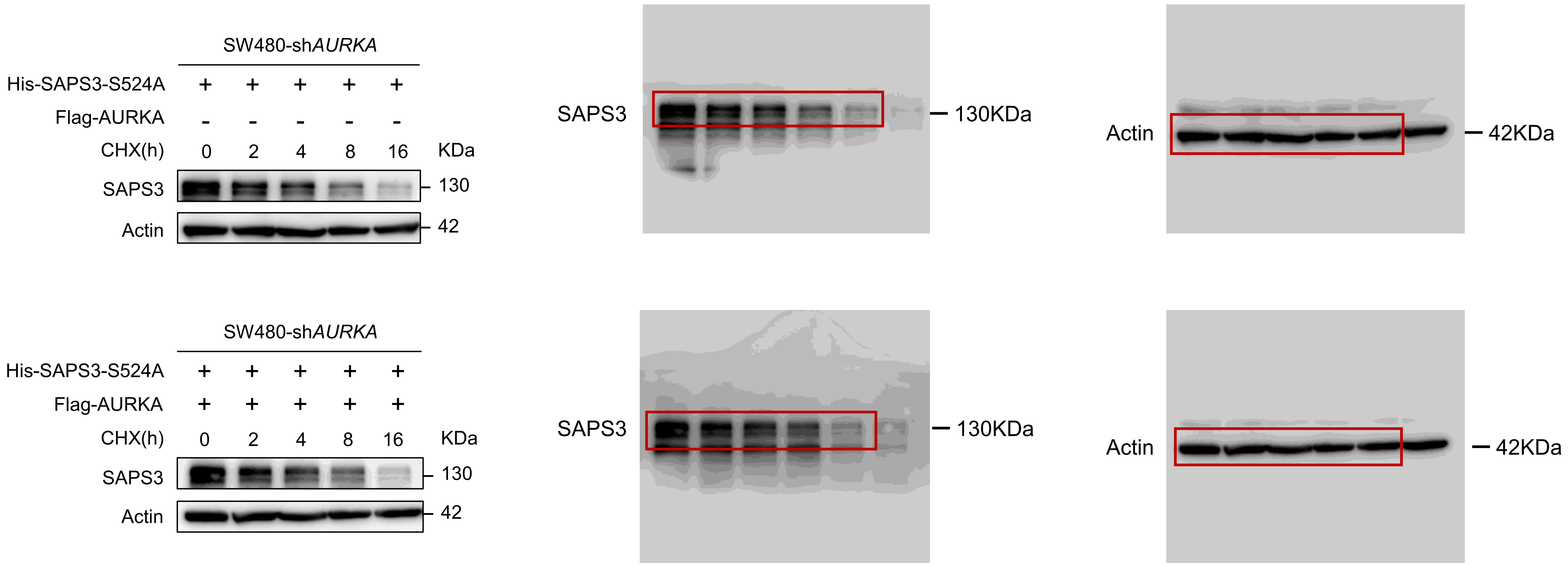
SW480-shAURKA
His-SAPS3-S524A + + + + +
Flag-AURKA - - - - -
CHX(h) 0 2 4 8 16
KDa
SAPS3
130
Actin
42
SAPS3
130KDa
Actin
42KDa
SW480-shAURKA
His-SAPS3-S524A + + + + +
Flag-AURKA + + + + +
CHX(h) 0 2 4 8 16
KDa
SAPS3
130
Actin
42
SAPS3
130KDa
Actin
42KDa

SFig.5Q

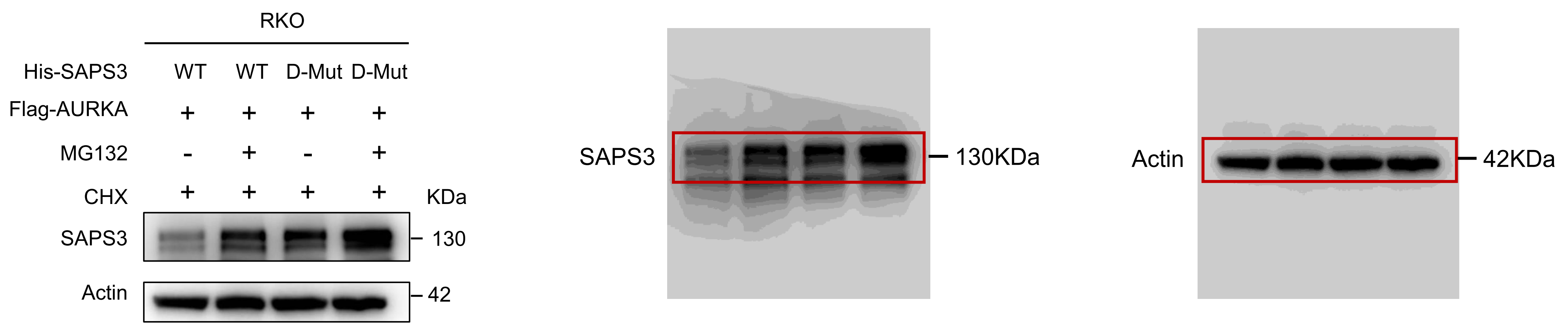

SFig.5R

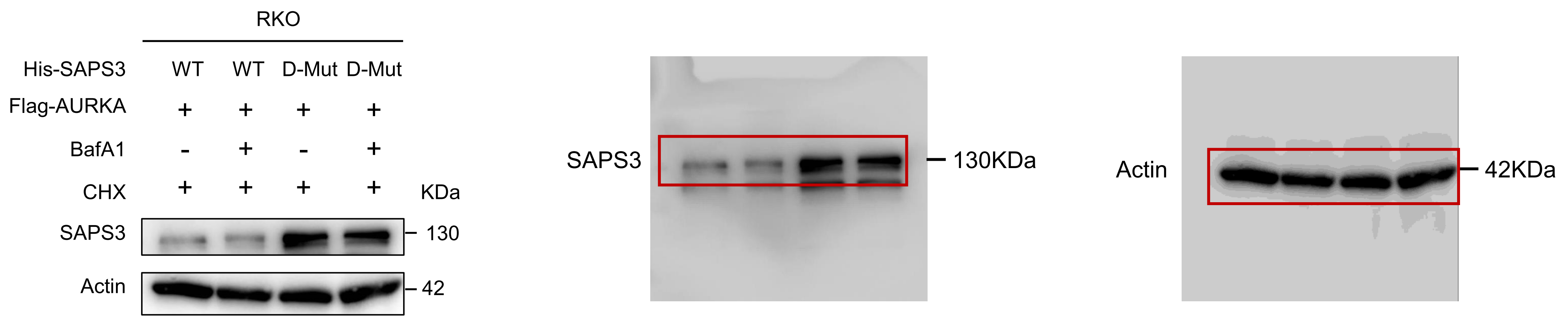

SFig.6D

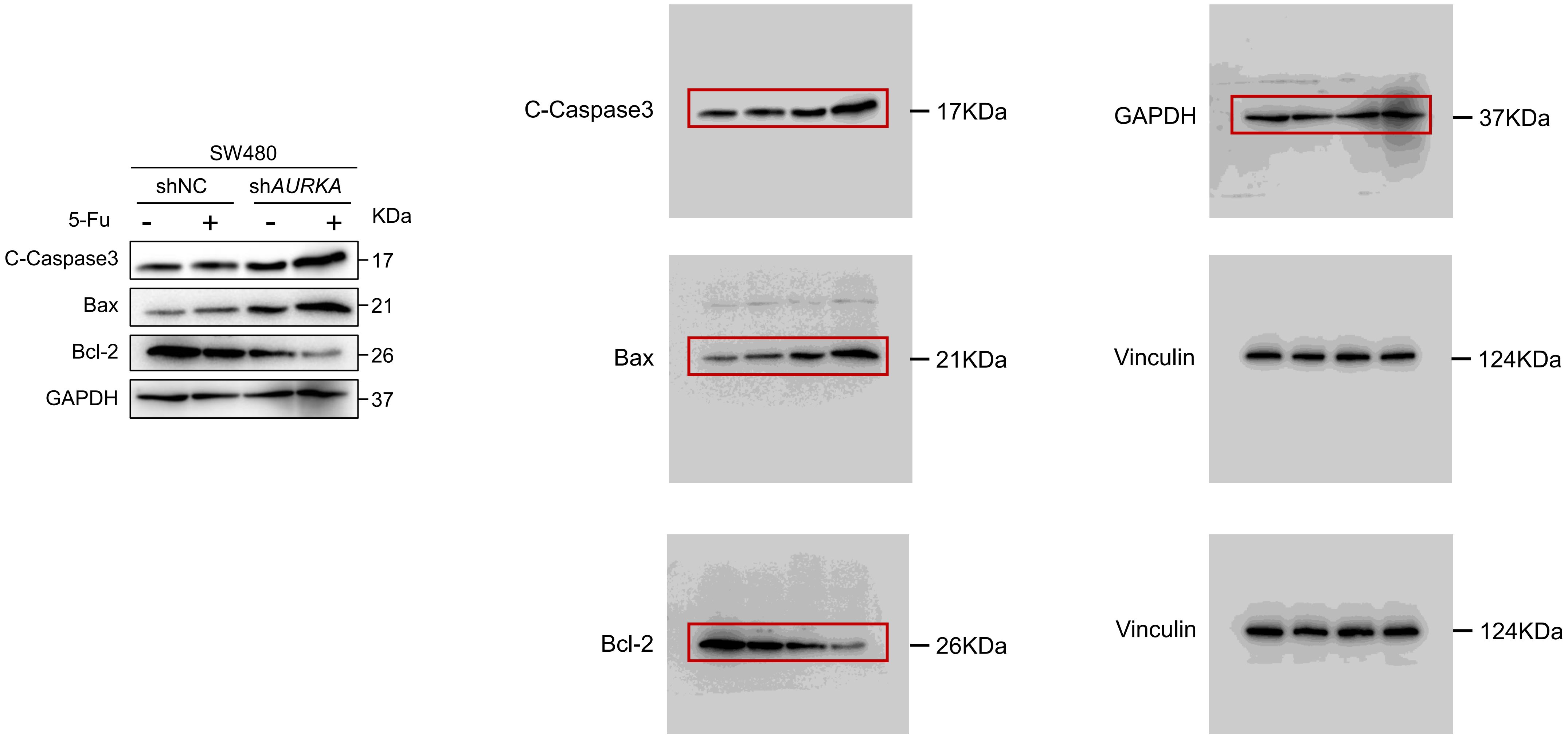
SW480
shNC
sh*AURKA*
5-Fu
-
+
-
+
KDa
C-Caspase3
17
Bax
21
Bcl-2
26
GAPDH
37
C-Caspase3
17KDa
GAPDH
37KDa
Bax
21KDa
Vinculin
124KDa
Bcl-2
26KDa
Vinculin
124KDa

SFig.6D

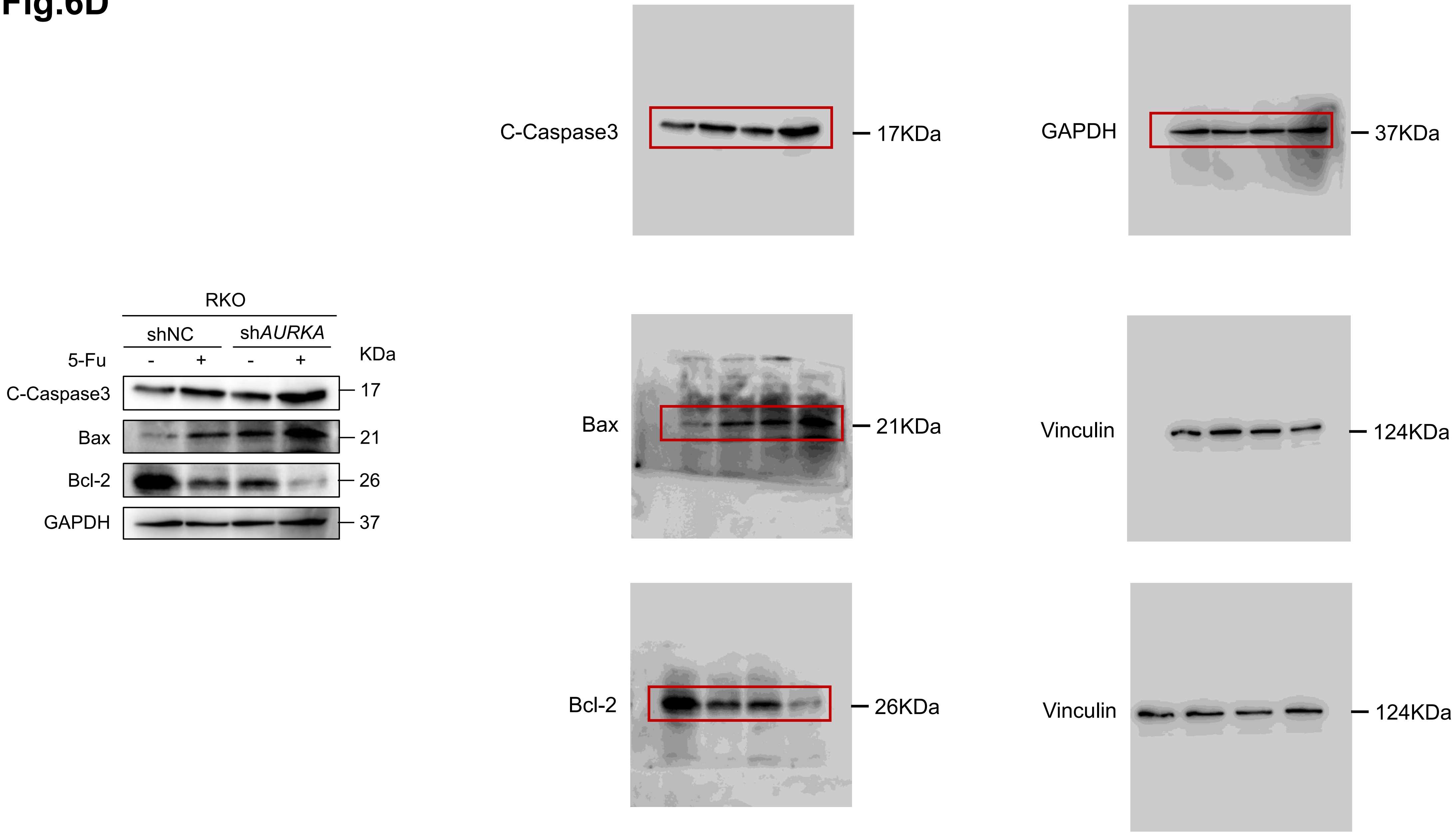
RKO
shNC
shAURKA
5-Fu
-
+
-
+
KDa
C-Caspase3
17
Bax
21
Bcl-2
26
GAPDH
37
C-Caspase3
17KDa
GAPDH
37KDa
Bax
21KDa
Vinculin
124KDa
Bcl-2
26KDa
Vinculin
124KDa

SFig.7A

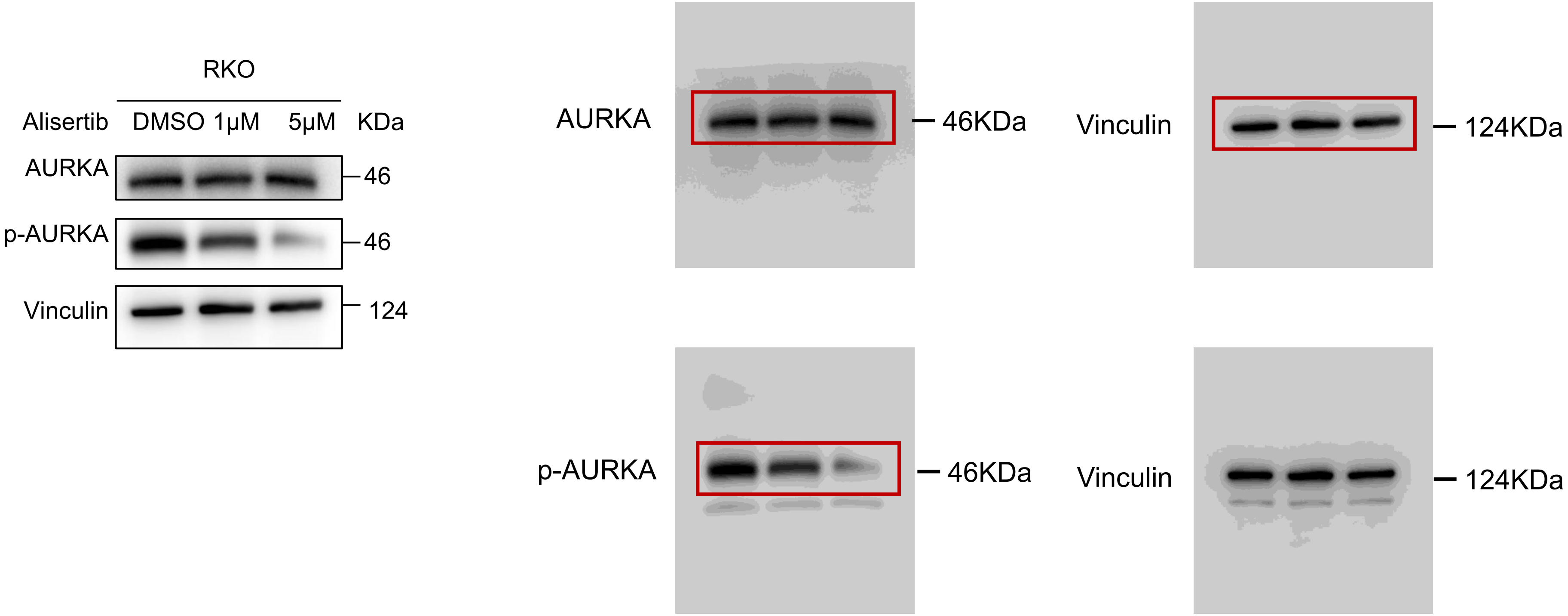
RKO
Alisertib
DMSO
1μM
5μM
KDa
AURKA
46
p-AURKA
46
Vinculin
124
AURKA
46KDa
Vinculin
124KDa
p-AURKA
46KDa
Vinculin
124KDa

SFig.7F

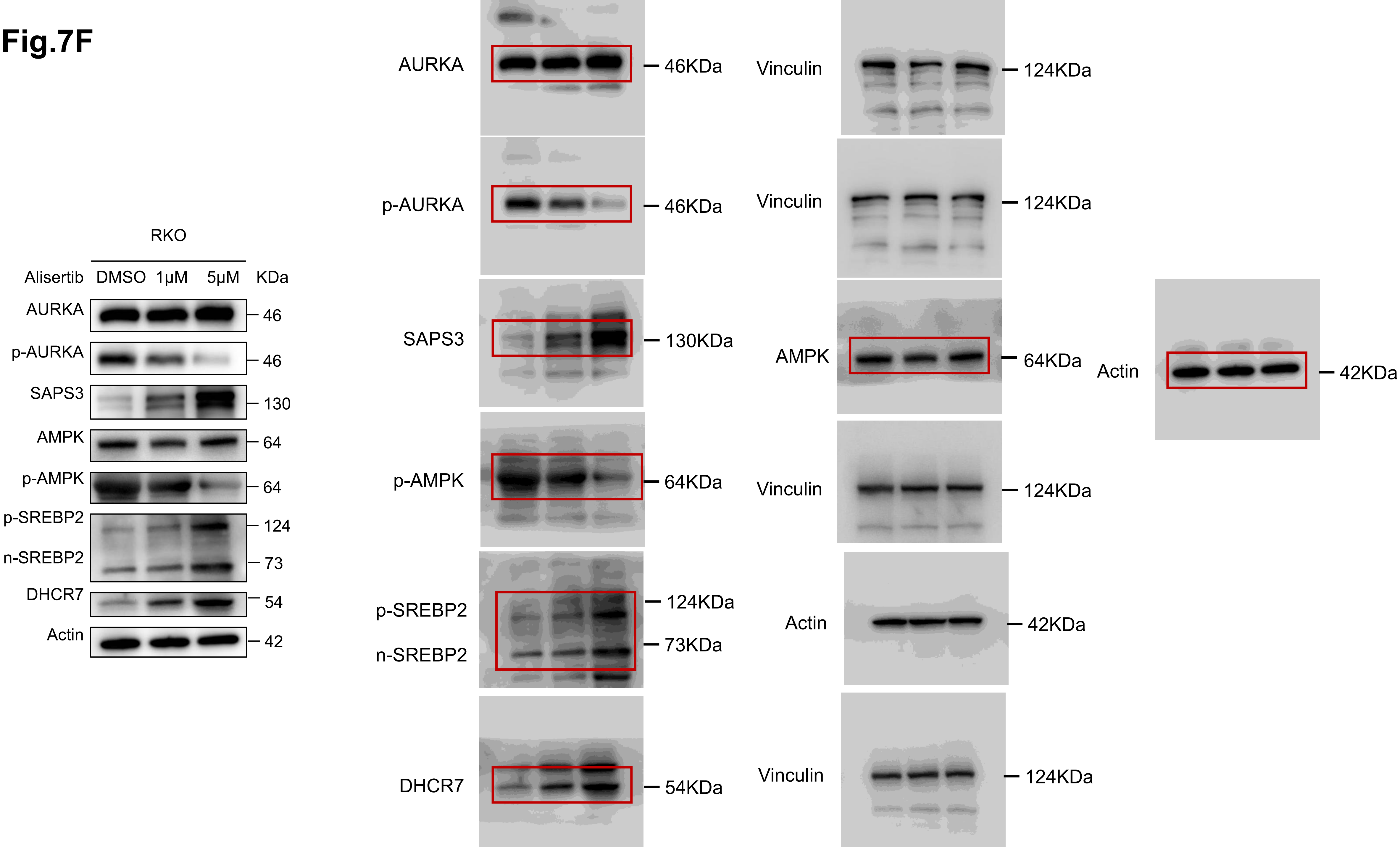
RKO
Alisertib DMSO 1μM 5μM KDa
AURKA 46
p-AURKA 46
SAPS3 130
AMPK 64
p-AMPK 64
p-SREBP2 124
n-SREBP2 73
DHCR7 54
Actin 42
AURKA 46KDa
Vinculin 124KDa
p-AURKA 46KDa
Vinculin 124KDa
SAPS3 130KDa
AMPK 64KDa
Actin 42KDa
p-AMPK 64KDa
Vinculin 124KDa
p-SREBP2 124KDa
n-SREBP2 73KDa
Actin 42KDa
DHCR7 54KDa
Vinculin 124KDa
